# In Situ Synthesis of Covalent Organic Frameworks Inside Cells for Triggering Ferroptosis and Immunotherapy

**DOI:** 10.1002/adma.202510663

**Published:** 2026-02-17

**Authors:** Qun Guan, Le‐Le Zhou, Zhiqing Yang, Yan‐An Li, Leyong Wang, Ruibing Wang

**Affiliations:** ^1^ State Key Laboratory of Mechanism and Quality of Chinese Medicine Institute of Chinese Medical Sciences University of Macau Taipa Macao China; ^2^ School of Chemistry and Pharmaceutical Engineering Medical Science and Technology Innovation Center Shandong First Medical University & Shandong Academy of Medical Sciences Jinan China; ^3^ College of Chemistry Chemical Engineering and Materials Science Collaborative Innovation Center of Functionalized Probes For Chemical Imaging in Universities of Shandong MoE Key Laboratory of Molecular and Nano Probes Shandong Normal University Jinan China; ^4^ Jiangsu Key Laboratory of Advanced Organic Materials College of Chemistry Nanjing University Nanjing China; ^5^ Department of Pharmaceutical Sciecnes and MoE Frontiers Science Center for Precision Oncology University of Macau Taipa Macao China

**Keywords:** cancer immunotherapy, covalent organic framework, ferroptosis, in situ synthesis, intracellular polymerization, lysosome, synthetic polymer

## Abstract

The synthesis of non‐natural polymers inside living systems has become an effective approach for controlling cellular functions and behaviors. However, in situ synthesis of crystalline polymers, such as covalent organic frameworks (COFs), inside cells using dynamic covalent chemistry faces challenges in aqueous environments, crystallization, and functionality. Herein, we present an intralysosomal crystalline polymerization strategy to synthesize COFs inside tumor cells through an acid‐catalyzed imine condensation reaction. The COFs synthesized intracellularly exhibit inherent fluorescence and well‐defined crystalline structures, allowing real‐time monitoring of the synthesis and product characterization. The formation of imine frameworks inside cell lysosomes induces lysosomal damage, activates lysosomal iron, and triggers immunogenic ferroptosis, thus enhancing antitumor immunotherapy. This strategy of intralysosomal crystalline polymerization facilitates the ambient synthesis of COFs, opening avenues for therapeutic polymerization reactions and providing insights into cellular responses to in situ polymer synthesis.

## Graphical abstract

We have developed an intracellular crystalline polymerization strategy involving acid‐catalyzed imine condensation to synthesize COFs in lysosomes. The generation of COFs in lysosomes damages their integrity, activates iron species, and induces immunogenic ferroptosis, thereby enhancing antitumor immunotherapy. This strategy facilitates ambient COF synthesis and provides insight into artificial chemical reactions within biological systems.

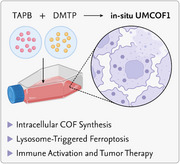

## 1 Introduction

Natural polymerization reactions are widespread in cells, and the resulting polysaccharides, proteins, and nucleic acids serve as the material basis for maintaining metabolism [1]. Artificial polymerization is a well‐established method for producing diverse and customizable functional polymers. It serves as a valuable model for replicating the structure and function of natural biological macromolecules, offering unique biophysical and biochemical properties not present in natural macromolecules through a bottom‐up approach [2]. Synthetic polymers have been utilized in various biological applications, including gene transfection, disease diagnosis, and cancer therapy [3].

Polymers synthesized inside living cells acquire unique shapes influenced by the cellular environment, allowing for precise and specific interactions with biological systems [4]. Introducing artificial polymerization reactions into cells offers unprecedented opportunities to study and control cellular metabolism [5, 6]. However, performing exogenous polymerization within living cells is challenging owing to the complexity of operating multiple monomers in aqueous, viscous, and dynamic compartments [7, 8]. Biologically active substances, including ionic species, proteins, and biothiols, prevalent in biological systems, can impede or even halt the polymerization process [9, 10, 11]. Furthermore, most polymerization reactions necessitate biohazardous stimuli, such as UV irradiation [12], metal ions [13, 14], and free radical initiators [15], and high monomer concentrations [16, 17], restricting intracellular polymerization to modulating cellular physiological behavior rather than functioning as a therapeutic tool [18, 19]. The resulting polymers typically lack crystallinity, making material characterization and defining structure–property relationships challenging [20, 21]. While some proof‐of‐concept covalent [22, 23, 24, 25, 26] and supramolecular [27, 28] polymerizations have been demonstrated within living cells, no evidence shows the intracellular synthesis of crystalline polymers or their role in cellular function or antitumor therapy.

Covalent organic frameworks (COFs) are porous crystalline polymer materials formed by the dynamic covalent polymerization of organic monomers [29, 30]. They have become part of a vast selection of biomedical materials for bioimaging [31, 32, 33], drug delivery [34, 35, 36], phototherapy [37, 38, 39, 40, 41], radiotherapy [42, 43, 44, 45, 46], and immunotherapy [47, 48, 49, 50], owing to their design flexibility, functionalization, and biocompatibility [51, 52]. Herein, we present an acid‐catalyzed crystalline polymerization reaction using biocompatible organic monomers for the in situ formation of imine‐linked COFs inside the lysosomes of living cells. The visible‐light fluorescence and well‐defined COF structures facilitate the tracing and characterization of intracellular polymerizations. Intralysosomal COF synthesis triggers immunogenic ferroptosis by activating lysosomal iron, thus enabling cancer immunotherapy in vivo. This approach sets a standard for the mild synthesis of COFs and offers a versatile platform for therapeutic polymerizations in living organisms.

## 2 Results

### 2.1 Aqueous‐Phase Synthesis of COFs

**FIGURE 1:**
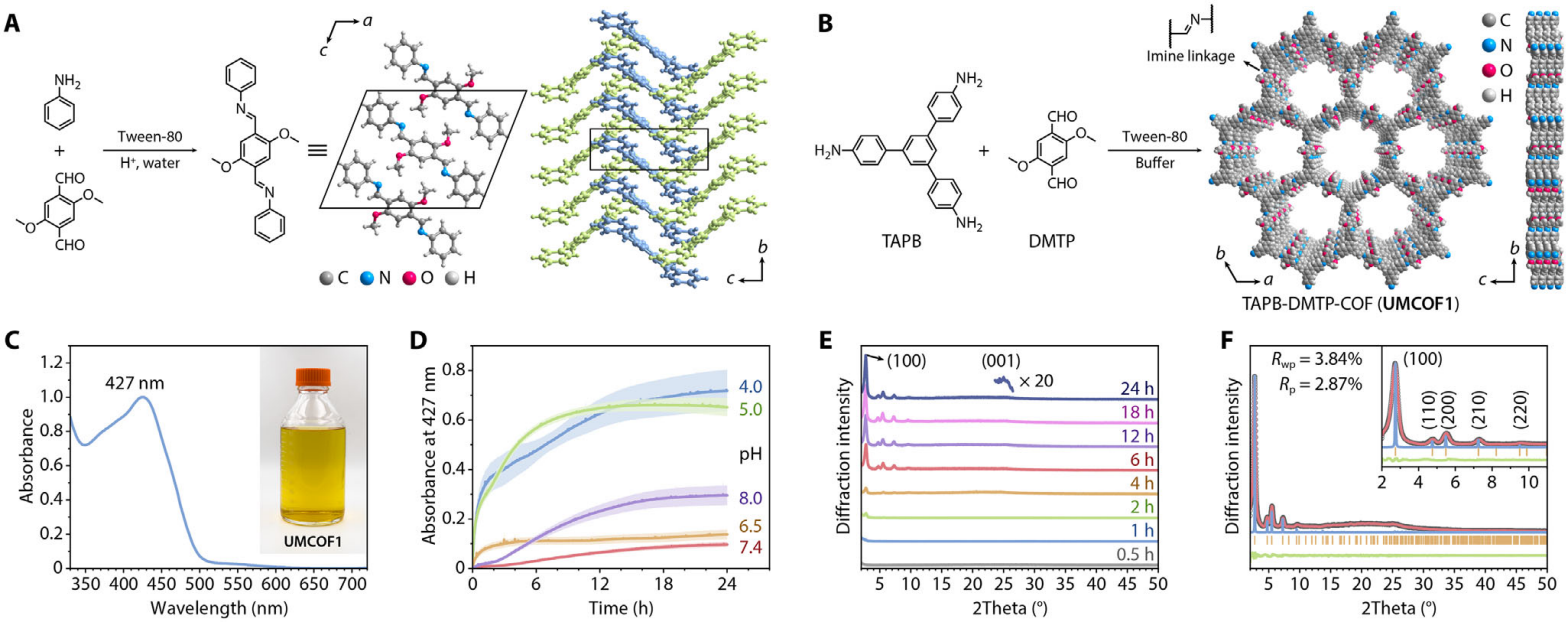
Synthesis of **UMCOF1** in a phosphate buffer. (A) Aqueous‐phase synthesis and crystal structure of the bisimine model compound (CDCC 2361348). (B) Synthesis and structure of **UMCOF1**. (C) UV–vis spectrum of **UMCOF1**. Inset: digital photographs of the reaction bottle charged with the phosphate buffer (10 mM, pH 5.0) containing TAPB (50 µM), DMTP (75 µM), and Tween‐80 (0.0213 vol. %) at 37°C for 24 h. (D) Time‐dependent absorbance of the reaction buffer solutions (pH = 4.0–8.0) at 427 nm. Data are expressed as mean ± SD; *n* = 4 independent experiments. (E) PXRD patterns of the products isolated at different reaction times. (F) Experimental (grey dots), Pawley‐refined (red), and simulated (blue) PXRD patterns; difference plot (green); and the Bragg positions (brown) of the **UMCOF1** powder. Inset: amplified curves in the range of 2*θ* = 2°–11°.

To verify the feasibility of the aqueous‐phase synthesis strategy, an imine model compound was synthesized. Reacting aniline with 2,5‐dimethoxyterephthalaldehyde (DMTP) in water, using acetic acid as a catalyst and Tween‐80 as a solubilizer, the desired binary imine was obtained in 45 % yield (Figure 1A; Figure S1). Single‐crystal X‐ray diffractometry revealed a parallel AA stacking arrangement among the molecules in the crystal (Table S1).

We selected TAPB‐DMTP‐COF, generated from 1,3,5‐tris(4‐aminophenyl)benzene (TAPB) and DMTP, to investigate imine‐linked COF synthesis in acidic buffer solutions (Figure 1B). This COF was first synthesized in 2015 by Jiang et al. [53] using a solvothermal reaction in a degassed Pyrex tube charged with 1,2‐dichlorobenzene and 1‐butanol at 120°C for 72 h. Here, TAPB and DMTP were mixed in a phosphate buffer (10 mM, pH 5.0, 37°C) with Tween‐80. The solution turned yellow, with a maximum absorption peak at 427 nm, indicating acid‐catalyzed imine polymerization (Figure 1C). Unlike the solvothermal synthesis, the mixture remained transparent and homogeneous, with no apparent precipitation. Kinetic tracing in buffer solutions with different pH values demonstrated that polymerization occurred rapidly at pH 4.0 and 5.0, reaching equilibrium within 6 h (Figure 1D). In contrast, polymerization was slow at pH 6.5 and 7.4. Because the base is also an effective catalyst for imine condensation, polymerization proceeded moderately at pH 8.0 [54]. Powder X‐ray diffraction (PXRD) patterns of the resulting product revealed a diffraction peak at 2*θ* = 2.74° at 6 h, gradually increasing with incubation (Figure 1E). This reaction involves early rapid polymerization and late slow crystallization, consistent with previously reported mechanisms [55].

To further isolate and analyze the product, triethylamine was added to the reaction mixture after 24 h of incubation at 37°C, precipitating the COF nanoparticles as an insoluble yellow powder (referred to as **UMCOF1**) with a 76 % yield. The crystal structure determined through PXRD in conjunction with computational simulations using the Forcite module in *BIOVIA Materials Studio 2018* closely matched the simulated AA stacking structure (**hcb** net; *P*3 space group, No. 143). A Pawley refinement on the PXRD pattern provided the cell parameters: *a* = *b* = 37.24 Å, *c* = 3.53 Å, *α* = *β* = 90°, and *γ* = 120°, with favorable agreement factors, *R*_wp_ = 3.84 % and *R*_p_ = 2.87 % (Figure 1F; Table S2). The permanent porosity, chemical structure, thermal stability, and microscopic morphology of **UMCOF1** were extensively analyzed and consistent with those of the simulated structures (Figure S2).

To demonstrate the versatility of this aqueous‐phase synthesis strategy, **UMCOF2** (from the reaction of TAPB and 2,5‐divinylterephthalaldehyde), **UMCOF3** (from the reaction of TAPB and benzene‐1,3,5‐tricarbaldehyde), **UMCOF4** (from the reaction of 4,4′,4″,4‴‐(porphyrin‐5,10,15,20‐tetrayl)tetraaniline and 2,5‐dihydroxyterephthalaldehyde), and **UMCOF5** (from the reaction of 4,4′,4″,4‴‐methanetetrayltetraaniline and terephthalaldehyde), were synthesized in phosphate or acetate buffer solutions in 27 %–66 % yields (Figure S3). The lattice parameters and atomic coordinates of the resulting COFs were determined through simulations based on the PXRD patterns (Table S3–S7). Similar to **UMCOF1**, the synthesized COFs demonstrated moderate‐to‐high crystallinity, permanent porosity, and well‐defined chemical structures (Figure S4).

### 2.2 Intracellular Synthesis of In Situ UMCOF1

**FIGURE 2:**
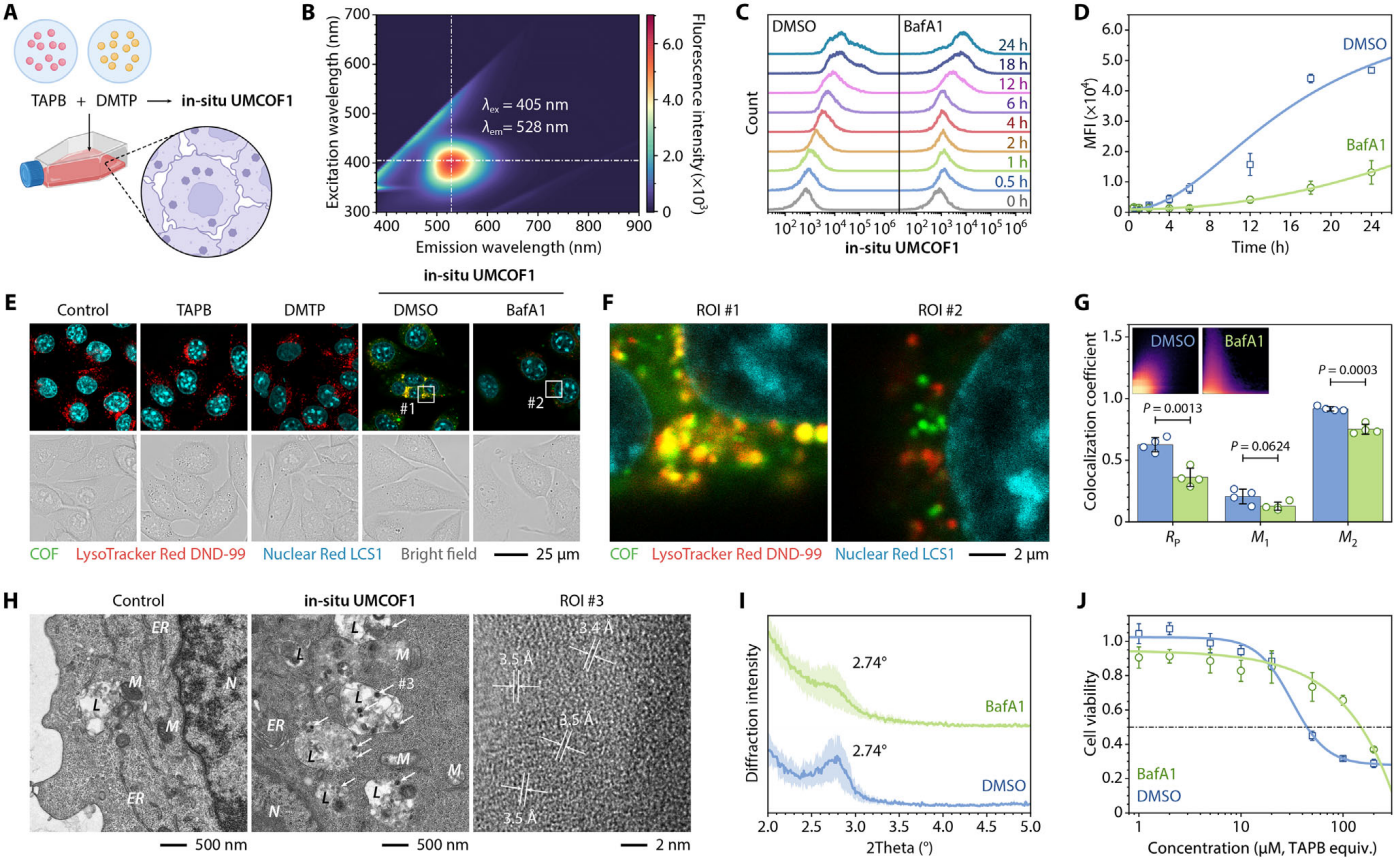
Synthesis of **in situ UMCOF1** inside 4T1 cells pretreated with DMSO (0.1 vol. %, 8 h) or BafA1 (100 nM, 8 h). (A) Schematic of the synthesis. (B) Excitation–emission matrix of **UMCOF1** in PBS. (C, D) Representative flow cytometric analysis (C) and cellular MFI (D) of 4T1 cells treated with TAPB (50 µM) and DMTP (75 µM) for varying incubation times. *n* = 3 biological replicates. (E–G) Representative confocal laser scanning fluorescence micrographs (E,F) and colocalization analysis between **in situ UMCOF1** and lysosomes (G) of 4T1 cells treated with TAPB (20 µM) and DMTP (30 µM) for 6 h. Inset: fluorescence intensity scatterplots obtained via pixel‐wise colocalization analysis (G). *n* = 4 biological replicates. (H) Bio‐TEM and lattice‐resolution TEM images of 4T1 cells treated with TAPB (50 µM) and DMTP (75 µM) for 24 h. The white arrows indicate **in situ UMCOF1** nanoparticles in lysosomes. Abbreviations: *N*, cell nucleus; *L*, lysosome; *M*, mitochondrion; *ER*, endoplasmic reticulum. (I) PXRD patterns of **in situ UMCOF1** isolated from the 4T1 cells treated with TAPB (50 µM) and DMTP (75 µM) for 24 h. *n* = 3 independent experiments. (J) CCK‐8 viability assays of 4T1 cells treated with TAPB (0–200 µM) and DMTP (0–300 µM) for 24 h. *n* = 4 technical replicates. Data are expressed as mean ± SD (D, G, I, J). Data are fitted with the logistic model (D, J). Statistical significance was calculated using the two‐tailed unpaired *t*‐test (G). Schematic created using BioRender.com (A).

The resulting **UMCOF1** nanoparticles exhibited a significant positive surface charge owing to the protonation of the imine linkages [56]. Lysosomal contents, with a pH of approximately 4.5–5.0 [57], are more acidic than the protonated **UMCOF1** (p*K*_a_ = 5.40 ± 0.09) (Figure S5). Hence, the potential self‐assembly of organic monomers inside living cells was investigated (Figure 2A). The excitation–emission matrix revealed that **UMCOF1** dispersed in phosphate‐buffered saline (PBS) could be excited by a 405 nm laser, producing a green fluorescence at 528 nm with a lifetime of 10.4 ns (Figure 2B; Figures S6 and S7). This enabled monitoring of intracellular COF formation.

4T1 cells incubated with TAPB and DMTP solubilized in Tween‐80 exhibited a progressive increase in intracellular fluorescence intensity over time, as shown by flow cytometry (Figure 2C). Tween‐80 not only acted as a solubilizer for organic monomers but also facilitated the cellular uptake of impermeable molecules (Figure S8). Premixing TAPB and DMTP in RPMI‐1640 medium and storing them for 1–7 days before incubation with 4T1 cells barely decreased intracellular fluorescence intensity, indicating predominant intracellular formation of **in situ UMCOF1** (Figure S9). Following an 8 h of pretreatment with Bafilomycin A1 (BafA1) to hinder lysosomal acidification, the fluorescence intensity of cells at 24 h decreased to approximately 28 % of that in unpretreated cells, suggesting a correlation between lysosomal acidity and COF formation (Figure 2D). Confocal laser scanning fluorescence microscopy revealed green punctate fluorescence within cells after 6 h of co‐incubation of the two monomers, co‐localizing with LysoTracker Red DND‐99‐labeled lysosomes with a Pearson correlation coefficient of *R*_P_ = 0.626 ± 0.058 (Figure 2E–G). BafA1 pretreatment not only decreased cellular fluorescence intensity but also reduced the colocalization level (*R*_P_ = 0.361 ± 0.074), confirming **in situ UMCOF1** formation in lysosomes. The Manders’ overlap coefficients, *M*_1_ and *M*_2_, showed similar results.

We demonstrated that the observed green fluorescence originated from the COF crystalline structure, not the amorphous polymer or intrinsic subcellular structures. Transmission electron microscopy (TEM) of the glutaraldehyde‐fixed cells showed high‐contrast spherical nanoparticles of diameter approximately 120 nm within the lysosomes (Figure 2H). Lattice‐resolution TEM revealed an interplanar spacing of 3.5 Å, consistent with the *π–π* stacking of **UMCOF1**. In addition, a pale yellow gel‐like material from the whole‐cell lysate exhibited the same fluorescence emission and a shortened fluorescence lifetime characteristic as **UMCOF1** (Figure S10). The gel‐like material applied to a silicon wafer for small‐angle PXRD showed a broad peak at 2.74°, confirming the crystallinity of **in situ UMCOF1** (Figure 2I). BafA1 pretreatment reduced its crystallinity and broadened the diffraction peak.

Real‐time live‐cell imaging demonstrated that **in situ UMCOF1** formation resulted in significant changes in cell morphology and proliferative capacity (Figure S11). Cell counting kit‐8 (CCK‐8) assays revealed that co‐incubation with TAPB and DMTP inhibited 4T1 cell proliferation and decreased cell viability, with an IC_50_ of approximately 44.3 µM (TAPB equiv.). BafA1 pretreatment reversed cell death, increasing the IC_50_ to approximately 149.9 µM (Figure 2J). Monomers alone and pre‐synthesized **UMCOF1** were non‐toxic to 4T1 cells (Figures S12 and S13). Interestingly, **in situ UMCOF1** synthesis in 4T1 multicellular spheroids caused positive propidium iodide (PI) staining, indicating cell death induced by intralysosomal COF synthesis (Figures S14 and S15). Similarly, it was synthesized in lysosomes of CT26, MCF7, HCT116, HT1080, and KYSE510 cell lines and the corresponding tumor spheroids, resulting in cell death with IC_50_ values of 19.3–47.4 µM (Figures S16 and S17). However, the intracellular COF synthesis was also toxic to HEEC‐ and MCF10A‐immortalised normal cell lines, showing no tumor‐selective toxicity in vitro (Figure S18).

### 2.3 Ferroptosis Induced by In Situ UMCOF1 Synthesis

**FIGURE 3:**
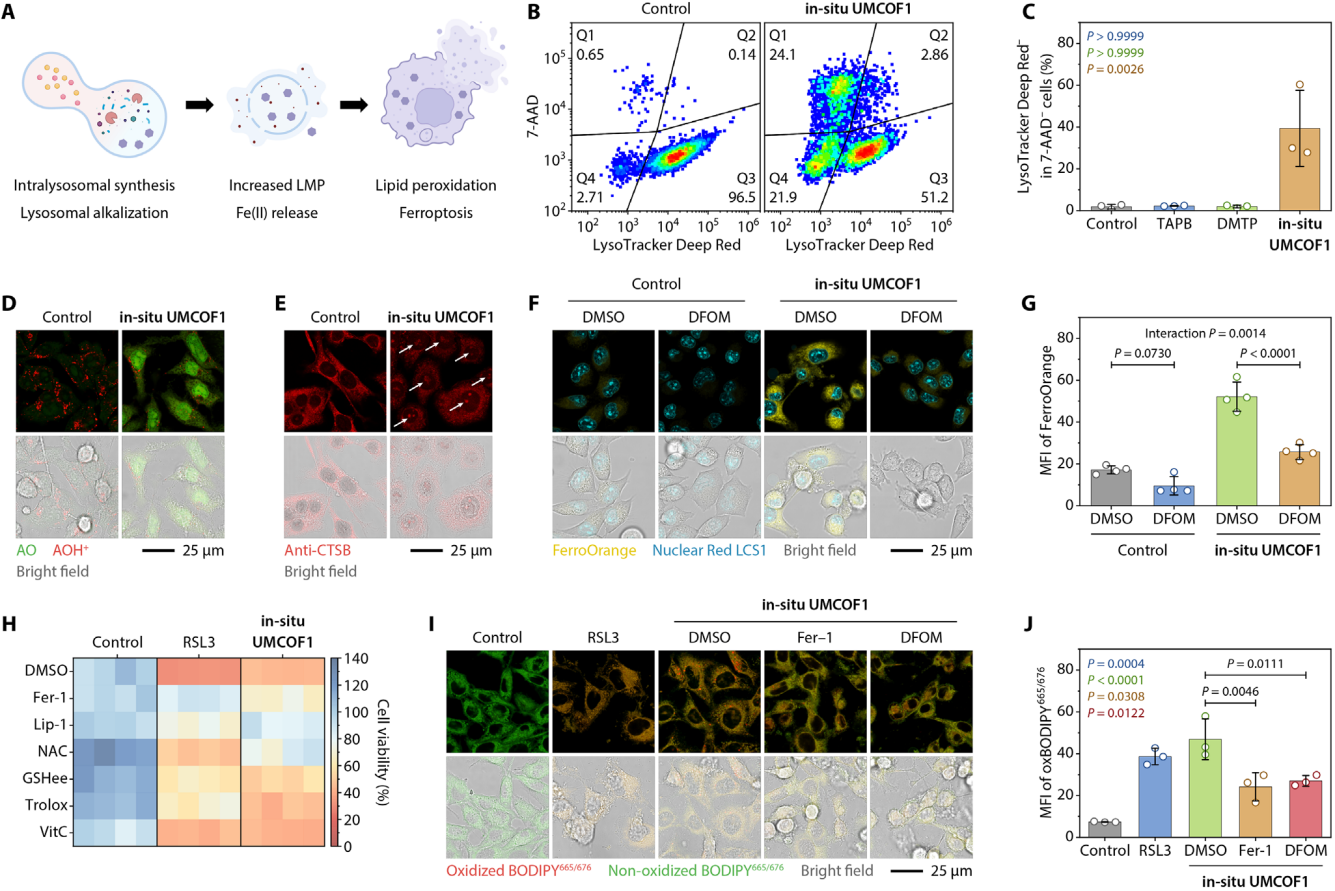
Lysosome‐mediated ferroptosis of 4T1 cells after treatment with TAPB (50 µM) and DMTP (75 µM) for 24 h. (A) Schematic of lysosome‐associated ferroptosis. (B,C) Representative flow cytometric analysis (B) and the quantification (C) revealing lysosomal alkalization in the treated 4T1 cells. *n* = 3 biological replicates. (D) Confocal laser scanning fluorescence micrographs of AO staining for assessing the lysosomal membrane permeability of the treated 4T1 cells. (E) Confocal laser scanning fluorescence micrographs of CTSB trafficking to the nucleus of the treated 4T1 cells. The white arrows indicate nucleus‐expressed CTSB. (F,G) Representative confocal laser scanning fluorescence images (F) and the corresponding quantification (G) of FerroOrange staining for detecting Fe(II) in the treated 4T1 cells in the absence or presence of DFOM (100 µM). *n* = 4 biological replicates. (H) Cell death rescue experiments of the treated 4T1 cells in the absence or presence of DMSO (0.1 vol. %), Fer‐1 (1.0 µM), Lip‐1 (0.5 µM), NAC (5.0 mM), GSHee (2.0 mM), Trolox (100 µM), and VitC (100 µM). *n* = 4 technical replicates. (I, J) Representative confocal laser scanning fluorescence images (I) and the corresponding quantification (J) of BODIPY^665/676^ staining for the lipid peroxidation analysis of the treated 4T1 cells in the absence or presence of DMSO (0.1 vol. %), Fer‐1 (1.0 µM), and DFOM (100 µM). *n* = 3 biological replicates. Cells treated with RSL3 (0.5 µM, 24 h) were used as the positive control (H–J) of ferroptosis. Data are expressed as mean ± SD (C, G, J). Statistical significance was calculated using two‐way ANOVA followed by Šídák's multiple comparison tests (G) and one‐way ANOVA followed by Tukey's multiple comparison tests (C,J). Schematic created using BioRender.com (A).

To explain the cytotoxicity observed during COF synthesis inside lysosomes, we hypothesized that the **in situ UMCOF1** formation and protonation induce lysosomal alkalinization, facilitate lysosomal membrane permeabilization (LMP), and stimulate lysosomal iron to increase the intracellular labile iron pool (LIP), leading to cellular lipid peroxidation and ferroptosis (Figure 3A) [58].

Co‐treatment with TAPB and DMTP reduced the intensity of LysoTracker Deep Red staining in 7‐aminoactinomycin D (7‐AAD)‐impermeable cells, indicating lysosomal alkalinization (Figure 3B,C). Additionally, acridine orange (AO) staining revealed a decrease in AO red fluorescence with the appearance of green fluorescence in the cytoplasm and nucleus, confirming effective LMP (Figure 3D). Immunofluorescence staining demonstrated that cathepsin B (CTSB), a lysosome‐derived cysteine protease [59, 60], was trafficked to the nucleus and even to the extracellular space of the co‐treated cells (Figure 3E). These results indicate that the **in situ UMCOF1** formation damages the lysosomal membrane.

LMP inevitably releases the lysosomal contents into the cytoplasm. Lysosomes are the main storage compartments for intracellular free Fe(II), playing a central role in iron homeostasis [61]. Co‐treatment with TAPB and DMTP increased the fluorescence intensity of FerroOrange, a cell‐permeable Fe(II) fluorescent probe, to three times that of the untreated cells, while the iron‐chelator deferoxamine mesylate (DFOM) reduced this fluorescence enhancement, confirming that **in situ UMCOF1** increased the intracellular LIP (Figure 3F,G). In addition to increasing Fe(II) levels, LMP also increased Ca(II) and Cu(II) levels (Figure S19). However, only DFOM restored cell viability impaired by **in situ UMCOF1** synthesis, whereas the Ca(II)‐chelator BAPTA‐AM and the Cu(II)‐chelator (NH_4_)_2_MoS_4_ were ineffective. This suggests that the increased LIP caused cell damage, consequently increasing Ca(II) and Cu(II) levels (Figure S20).

We assessed **in situ UMCOF1**‐induced ferroptosis. The Class‐II ferroptosis inducer RSL3, a glutathione peroxidase 4 (GPX4) inhibitor, served as a positive control for ferroptosis [62]. Cell rescue experiments demonstrated that ferroptosis inhibitors ferrostatin‐1 (Fer‐1) and liproxstatin‐1 (Lip‐1), the cysteine precursor *N*‐acetylcysteine (NAC), the cell‐permeable glutathione (GSH) precursor GSH ethyl ester (GSHee), and the lipophilic antioxidant Trolox prevented cell death caused by both RSL3 treatment and TAPB and DMTP co‐treatment (Figure 3H; Figure S21). Conversely, hydrophilic vitamin C (VitC) had a limited rescue effect. Necroptosis inhibitors necrostatin‐1s (Nec‐1s) and necrosulfonamide, along with pyroptosis inhibitors VX765 and Ac‐FLTD‐CMK, were ineffective in rescuing cell death (Figure S22). Furthermore, cell death occurred irrespective of caspase 3 activation (Figure S23). Co‐treatment with TAPB and DMTP led to phosphatidylserine externalization; however, inhibiting apoptosis with the pan‐caspase inhibitor Z‐VAD‐FMK (zVAD) did not affect phosphatidylserine externalization, short‐term cell viability, or long‐term clonogenic ability. This suggests that the apoptosis‐associated pathway was activated but not essential for cell death.

Lipid peroxidation was assessed to confirm ferroptotic cell death [63, 64]. BODIPY 665/676 staining and confocal laser scanning fluorescence imaging showed that both RSL3 treatment and TAPB and DMTP co‐treatment led to lipid peroxyl radical accumulation in 4T1 cells, visualized as intensified red fluorescence with different distribution patterns (Figure 3I,J). RSL3 triggered filamentous fluorescence distributed throughout the endoplasmic reticulum, nuclear membrane, and plasma membrane. This aligns with the findings of Stockwell et al. [65], suggesting that lipid peroxidation initially accumulates in the endoplasmic reticulum membrane before spreading to other membrane structures. However, **in situ UMCOF1** induced both similar filamentous fluorescence and punctate fluorescence distributed throughout the lysosomes, which was attenuated by Fer‐1 and DFOM. Therefore, **in situ UMCOF1**‐induced lipid peroxidation is closely associated with lysosomal iron, in contrast to conventional Class‐II ferroptosis inducers.

Additionally, we confirmed **in situ UMCOF1**‐induced ferroptosis through gene transcription, protein expression, and mitochondrial morphology and function (Figures S24–S27).

### 2.4 Immunogenicity of Ferroptotic Cells

**FIGURE 4:**
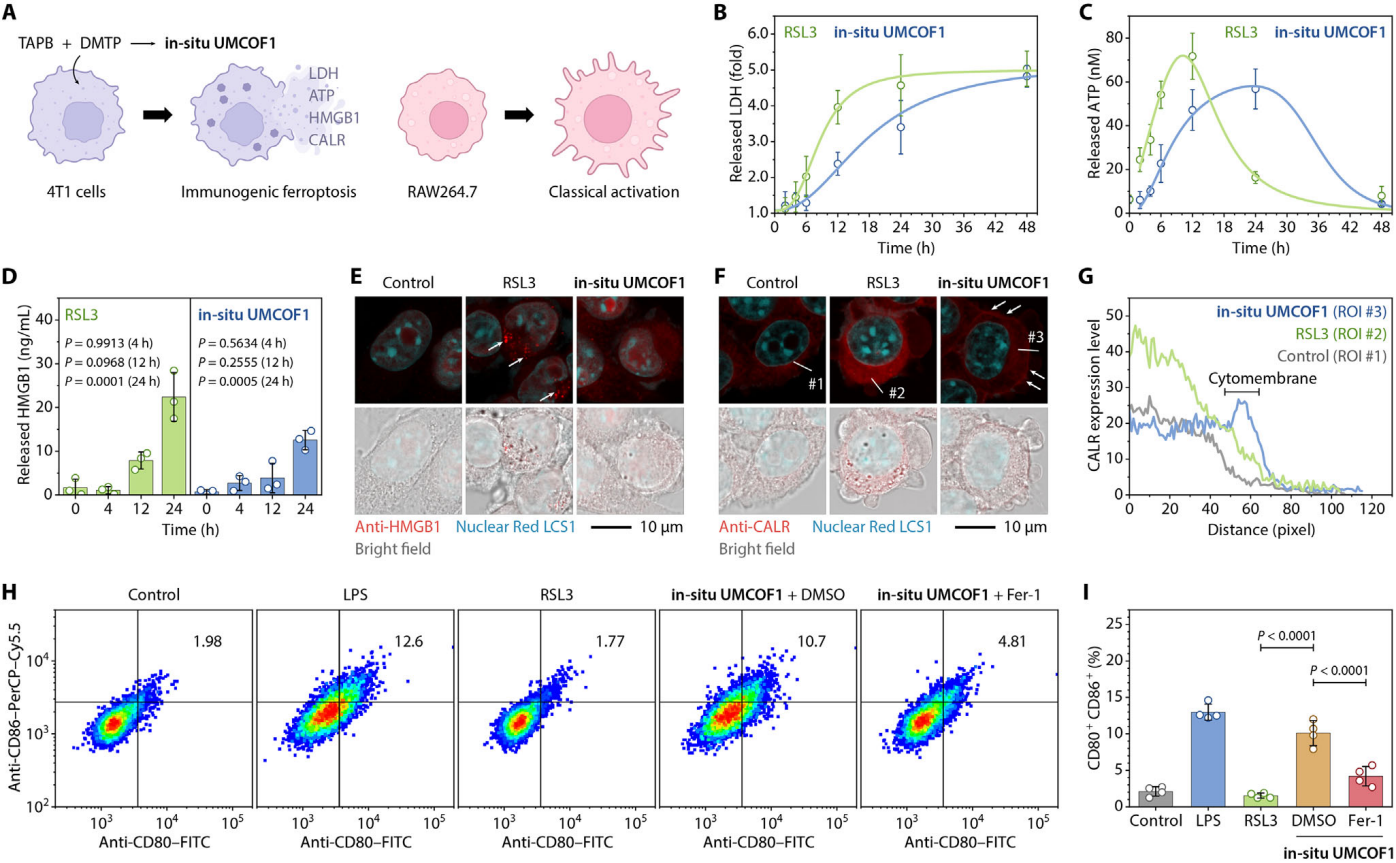
Immunogenicity of ferroptotic 4T1 cells. (A) Schematic of the DAMP releases and their subsequent effects on RAW264.7 macrophages. (B–D) Contents of LDH (B), ATP (C), and HMGB1 (D) released from the 4T1 cells after 0–24 h treatment with TAPB (50 µM) and DMTP (75 µM). *n* = 3 biological replicates. (E–G) Immunofluorescence staining of HMGB1 (E) and CALR (F) in the 4T1 cells treated with TAPB (50 µM) and DMTP (75 µM) for 24 h, with a graph of the pixel intensities (G) along the selected lines within the CALR staining (F). (H,I) Representative flow cytometric analysis (H) and the CD80^+^ CD86^+^ double‐positive cell percentages (I) of the RAW264.7 macrophages induced by the culture supernatant of the 4T1 cells after the treatment with TAPB (50 µM) and DMTP (75 µM) in the absence or presence of DMSO (0.1 vol. %), Fer‐1 (1.0 µM), and DFOM (100 µM) for 24 h. *n* = 4 biological replicates. The 4T1 cells treated with RSL3 (0.5 µM, 24 h) were used as the positive control (B–I) of ferroptosis. Data are expressed as mean ± SD (B, C, D, I). Data are fitted with the logistic (B) and BiHill (C) models. Statistical significance was calculated using one‐way ANOVA followed by Dunnett's (D) and Tukey's (I) multiple comparison tests. Schematics were created using BioRender.com (A).

Ferroptosis immunogenicity is significantly context‐dependent [66]. Given the varying sites of lipid peroxidation induced by RSL3 and **in situ UMCOF1**, we compared the immunogenicity and macrophage activation resulting from these two ferroptosis‐inducing agents (Figure 4A).

Lipid peroxidation‐induced membrane damage released damage‐associated molecular patterns (DAMPs) [67], including lactate dehydrogenase (LDH), adenosine triphosphate (ATP), and high mobility group box 1 (HMGB1), following RSL3 treatment and TAPB and DMTP co‐incubation (Figure 4B–D). However, the release kinetics demonstrated that the latter released DAMPs more slowly and continuously, with RSL3‐triggered ATP release occurring before plasma membrane permeabilization. Immunofluorescence staining demonstrated that RSL3 induced the translocation of HMGB1 from the nucleus to the cytoplasm through vesicle‐like structures, which were eventually released into the extracellular space (Figure 4E). TAPB and DMTP co‐incubation caused HMGB1 translocation, but without vesicles. TAPB or DMTP treatment alone did not induce ferroptosis‐related immunogenic responses (Figure S28). Furthermore, co‐incubation with TAPB and DMTP resulted in the upregulation of calreticulin (CALR) expression, both intracellularly and on the plasma membrane (Figure 4F,G). The CALR levels in RSL3‐treated cells were upregulated, but their expression was limited to the intracellular compartment, with no membrane expression detected. Flow cytometry confirmed CALR exposure outside the plasma membrane in the cells co‐incubated with TAPB and DMTP, hindered by Fer‐1 and DFOM (Figure S29). Conversely, minimal CALR exposure occurred in non‐permeabilized RSL3‐treated cells. These results indicate that while both treatments released DAMPs, ferroptosis induced by **in situ UMCOF1** synthesis in lysosomes was immunogenic, whereas RSL3‐triggered ferroptosis in the endoplasmic reticulum was not. This suggests that the ferroptosis immunogenicity may depend on the lipid peroxidation site and DAMP release kinetics.

The ferroptotic cell immunogenicity was assessed by collecting their culture supernatants and co‐culturing them with RAW264.7 macrophages (Figures S30–S32). Lipopolysaccharide served as a positive control for classical activation of RAW264.7 macrophages [68]. The supernatant from 4T1 cells treated with TAPB and DMTP, but not RSL3, released immune factors, including nitric oxide, interleukin 6 (IL6), and tumor necrosis factor α (TNFα), from RAW264.7 macrophages, confirming classical activation. This also increased intracellular reactive oxygen species, upregulated *Il6* and *Nos2* transcriptionally, and promoted membrane expression of CD80 and CD86 (Figure 4H,I), thereby improving phagocytosis for 4T1 cells and coumarin 6‐labeled polystyrene microspheres.

### 2.5 Intratumoral In Situ UMCOF1 Synthesis for Antitumor Therapy

**FIGURE 5:**
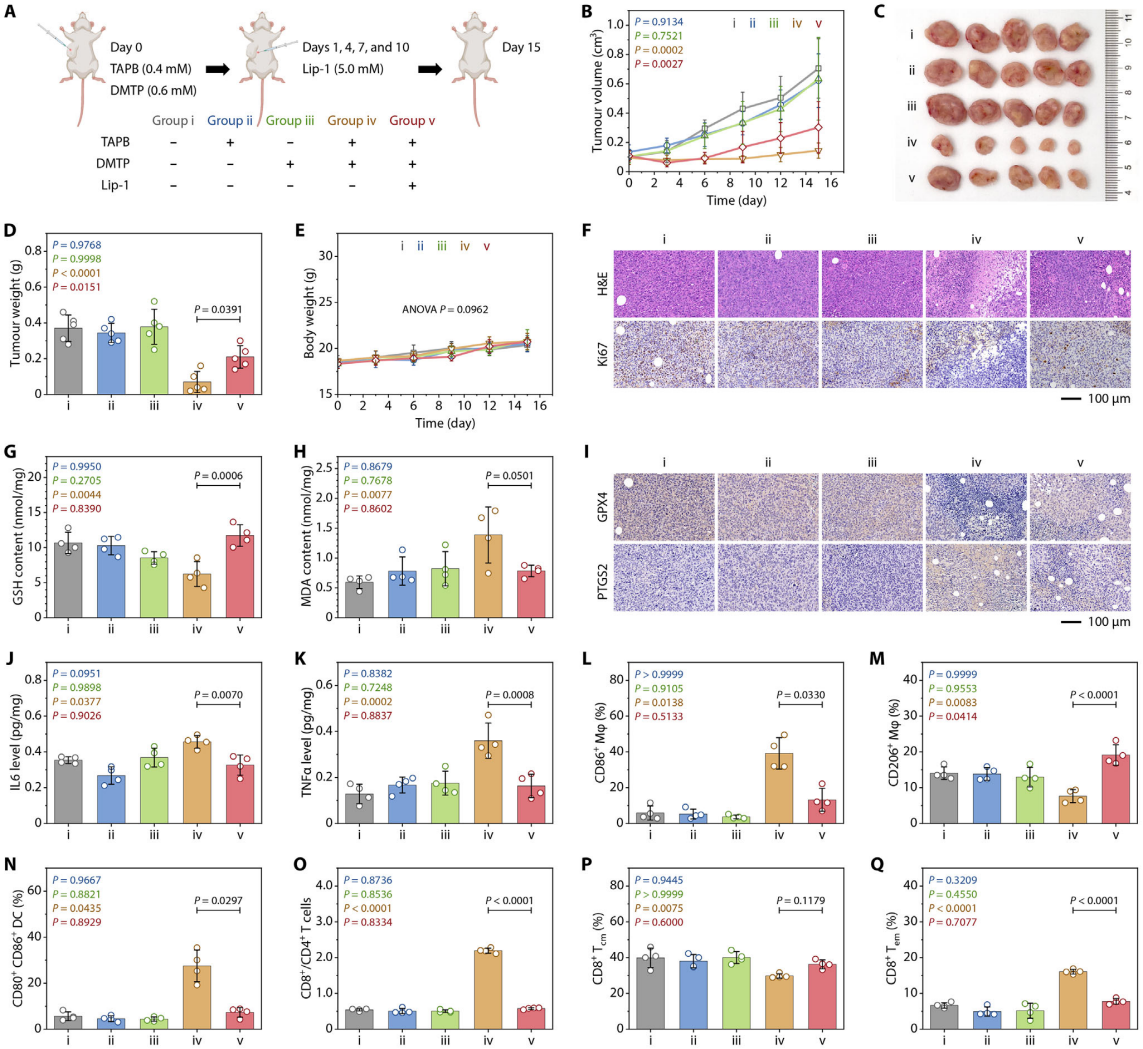
Antitumor therapy and immune response induced by intratumoral synthesis of **in situ UMCOF1** in 4T1 tumor‐bearing mice. (A) Therapeutic schedule involving a single intratumoral injection of a mixture (50 µL) of TAPB (0.4 mM) and DMTP (0.6 mM), as well as a peritumoral injection of Lip‐1 (5.0 mM, 50 µL). (B) Changes in the tumor volume with different treatments. (C, D) Photographs (C) and weights (D) of tumors excised from mice from various groups at the treatment endpoint. (E) Body weight changes of mice with different treatments. (F) Representative H&E and Ki67 immunohistochemical staining in the tumor at Day 15. (G, H) Intratumoral GSH (G) and MDA (H) levels of mice in various groups at Day 15. (I) Representative immunohistochemical staining of GPX4 and PTGS2 in the tumor at Day 15. (J, K) IL6 (J) and TNFα (K) levels in the tumors excised from mice in different treatment groups at Day 15. (L, M) Percentages of CD86^+^ (L) and CD206^+^ (M) cells in F4/80^+^ CD11b^+^ CD45^+^ spleen cells of mice in various groups at Day 15. (N) Percentages of CD80^+^ CD86^+^ double‐positive DCs in the CD11c^+^ CD45^+^ spleen cells of mice in various groups at Day 15. (O) Ratio of CD8^+^ T cells to CD4^+^ T cells in the spleen of mice in various groups at Day 15. (P, Q) Percentages of CD44^+^ CD62L^+^ (P) and CD44^+^ CD62L^−^ (Q) cells in the CD8^+^ CD3^+^ CD45^+^ spleen cells of mice in various groups at Day 15. Data are expressed as mean ± SD; *n* = 5 (B–E) or 4 (G, H, J–Q) mice per group. Statistical significance was determined using one‐way repeated measures ANOVA followed by Dunnett's multiple comparison test (B, E) and one‐way ANOVA followed by Tukey's multiple comparison test (D, G, H, J–Q). Schematic created using BioRender.com (A).

To evaluate the intratumoral **in situ UMCOF1** synthesis and its induced immunogenic ferroptosis, we constructed a unilateral 4T1 subcutaneous tumor model in BALB/c mice. Once the tumor volume reached 100 mm^3^, TAPB and DMTP dissolved in normal saline were intratumorally injected (Figure 5A). It is notable that at 24 h post‐injection, spherical particles with diameters ranging from 100 to 200 nm and a porous structure were observed in the tumor sections (Figure S33). These particles were distributed in the lysosomes of intact tumor cells and near the nuclei of damaged tumor cells, confirming intralysosomal **in situ UMCOF1** synthesis and its induced cell damage. After 15 days of TAPB and DMTP co‐injection, the tumor weights were merely 19 % of those in the saline‐injected group (Figure 5B–D). Conversely, TAPB or DMTP alone did not exhibit a significant therapeutic effect on 4T1 tumor‐bearing mice. Mouse body weight remained stable throughout the treatment period, liver and kidney functions were within normal ranges, and no signs of haemolysis were observed, indicating minimal cachexia and an acceptable level of systemic toxicity (Figure 5E; Figures S34 and S35).

Pathological analysis of the tumors aligned with the tumor growth curves (Figure 5F). Hematein–eosin (H&E) staining demonstrated that co‐injection with TAPB and DMTP caused ferroptosis and necrosis‐like injury, characterized by karyopyknosis, cytoplasmic vacuolization, and plasma membrane rupture. Immunohistochemical staining revealed a significantly lower percentage of Ki67‐positive cells in the co‐injection group than in the TAPB‐ or DMTP‐injected group, suggesting impaired proliferative potential. Furthermore, H&E staining of major organs revealed minor inflammation in the treatment group without any signs of tumor metastasis (Figure S36).

Peritumoral injection of the ferroptosis inhibitor Lip‐1 on 1, 4, 7, and 10 days after **in situ UMCOF1** treatment partially restored tumor progression. Injection of Lip‐1 alone had a negligible effect on tumor progression (Figure S37). In **in situ UMCOF1**‐treated tumor tissues, GSH content and GPX4 expression decreased, while malondialdehyde (MDA) and prostaglandin‐endoperoxide synthase 2 (PTGS2) expression increased (Figure 5G–I), confirming ferroptosis in vivo.

Enzyme‐linked immunosorbent assays revealed higher levels of IL6 and TNFα in tumor tissues following combined injections of TAPB and DMTP compared to untreated tumors (Figure 5J,K). Prostaglandin E2 (PGE2) and interferon γ (IFNγ) concentration changes were statistically insignificant (Figure S38). Flow cytometric immunophenotyping revealed that 39.2 % of splenic macrophages underwent classical activation, enhancing antitumor therapy by approximately 6.6 times compared to the untreated group, while alternatively activated macrophages decreased (Figure 5L,M; Figures S39 and S40). Mature dendritic cells (DCs) in the spleen increased by 4.9 fold post‐treatment, significantly boosting T cell activation (Figure 5N; Figure S41). T‐cell phenotyping revealed an increased and decreased percentage of cytotoxic T (Tc) and helper T (Th) cells, respectively, resulting in an elevated Tc to Th cell ratio, suggesting effective stimulation of cellular immunity (Figure 5O; Figure S42). Immunohistochemical staining showed that the treatment enhanced infiltration of Tc and Th cells in tumor tissues, signifying tumor microenvironment remodeling (Figure S43). Moreover, the treatment facilitated the Tc cell differentiation into effector memory T cells and activated central memory T cells, essential for long‐term immunity (Figure 5P,Q). In summary, these findings indicate that **in situ UMCOF1**‐induced immunogenic ferroptosis enhances macrophage activation, DC maturation, and tumor infiltration of T cells, thereby promoting immune activation and memory.

Further experiments showed that **in situ UMCOF1** could be synthesized inside B16F10 melanoma cells and tumors of C57BL/6N mice, inducing anti‐tumor ferroptotic immunotherapy (Figures S44 and S45). Although macrophage polarization was similar between the treated and untreated groups, we found that intracellular synthesis of **in situ UMCOF1** promoted DC maturation and increased the Tc to Th cell ratio (Figures S46–S49).

### 2.6 Abscopal Effects and Vaccine Efficacy of In Situ UMCOF1 Immunotherapy

**FIGURE 6:**
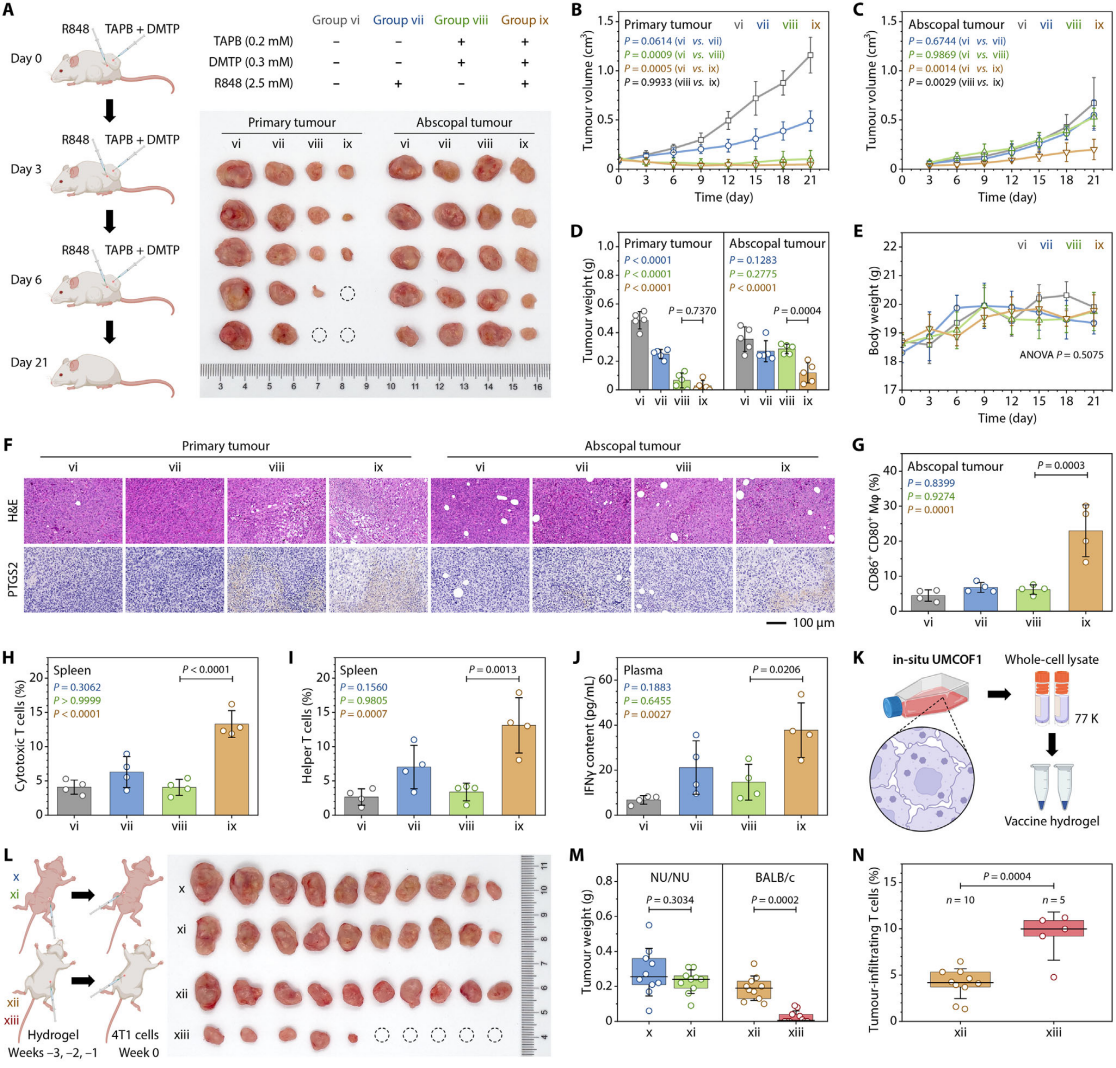
Abscopal effects and vaccine efficacy of **in situ UMCOF1**‐associated antitumor immunotherapy. (A–J) An intratumoral injection of TAPB (0.2 mM, 50 µL) and DMTP (0.3 mM, 50 µL) combined with a peritumoral injection of R848 (2.5 mM, 50 µL) for inducing abscopal effects in bilateral tumor‐bearing mice. (A) Therapeutic schedule and a photograph of the tumors excised from mice from various groups at the treatment endpoint. (B,C) Growth curves of primary tumors (B) and abscopal tumors (C). (D) Weight of the collected tumors at Day 21. (E) Changes in the body weight of mice with different treatments. (F) Representative H&E and PTGS2 immunohistochemical staining in the tumor at Day 21. (G) Percentages of CD80^+^ CD86^+^ double‐positive cells in the F4/80^+^ CD11b^+^ CD45^+^ cells from the abscopal tumors in various groups at Day 21. (H) CD3^+^ CD8^+^ double‐positive cells in the CD45^+^ spleen cells of mice in various groups at Day 21. (I) CD3^+^ CD4^+^ double‐positive cells in the CD45^+^ spleen cells of mice in various groups at Day 21. (J) IFNγ level in plasma obtained from the submandibular vein of mice in different treatment groups at Day 21. (K–N) Whole‐cell lysates of the 4T1 cells treated with TAPB (50 µM) and DMTP (75 µM) as tumor vaccines for protecting the BALB/c mice (but not the NU/NU nude mice) from the 4T1 tumor challenge. (K,L) Experimental scheme (K) and a photograph of the collected tumors (L) from the NU/NU nude mice (Groups x and xi) and BALB/c mice (Groups xii and xiii). Groups x and xii received a subcutaneous injection of control hydrogel (50 µL). Groups xi and xiii received a subcutaneous injection of the vaccine hydrogel (50 µL). (M) Weight of the collected tumors. Interaction *p* = 0.0469. (N) Percentages of the CD3^+^ cells in the CD45^+^ cells from the collected tumors. The middle line in the boxplot indicates the median, the whiskers represent SD, and the lower and upper hinges correspond to the first and third quartiles, respectively. Data are expressed as mean ± SD; *n* = 5 (A–E), 4 (G–J), and 10 (L,M) mice per group. Statistical significance was determined using one‐way repeated measures ANOVA followed by Tukey's multiple comparison test (B,C,E), two‐way ANOVA followed by Tukey's multiple comparison test (D), one‐way ANOVA followed by Tukey's multiple comparison test (G–J), two‐way ANOVA followed by Šídák's multiple comparison test (M), and two‐tailed unpaired t test (N). Schematics created using BioRender.com (A, K, L).

After demonstrating the antitumor effects and in vivo immunogenicity of **in situ UMCOF1**‐induced ferroptosis, we established a bilateral model of 4T1 tumors to combine intratumoral **in situ UMCOF1** synthesis with resiquimod (R848), a Toll‐like receptor 7/8 agonist [69], to extend local oncotherapy for systemic cancer management. The treatment plan involved intratumoral TAPB and DMTP injections and peritumoral R848 injection every 3 days for three doses (Figure 6A). Despite low‐dose injections, primary tumor growth was completely inhibited after three TAPB and DMTP injections, with or without R848 co‐treatment, consistent with the trend observed in the unilateral tumor model (Figure 6B). However, owing to limited immune infiltration in 4T1 tumors, neither TAPB and DMTP co‐injection nor R848 alone inhibited abscopal tumor growth, showing no significant difference from the control group (Figure 6C). Synergistic treatment with TAPB, DMTP, and R848 significantly delayed the growth of abscopal 4T1 tumors, exhibiting an abscopal effect (Figure 6D). No weight loss occurred in the treatment group, indicating no systemic toxicity at the current therapeutic dose (Figure 6E). Histological analysis revealed that TAPB and DMTP co‐injection damaged primary tumor tissues and PTGS2 expression, while synergistic **in situ UMCOF1** and R848 co‐treatment produced the most significant tissue damage and highest PTGS2 expression in abscopal tumors (Figure 6F).

The inhibition of abscopal tumors confirms the induction of a systemic antitumor immune response. Direct evidence showed that CD86^+^ CD80^+^ double‐positive macrophages in abscopal tumors increased only with the synergistic treatment, suggesting enhanced infiltration of classically activated macrophages in abscopal tumors (Figure 6G; Figure S50). Compared with the control group, the percentages of Tc and Th cells significantly increased (Figure 6H,I; Figure S51). The levels of IFNγ and TNFα in plasma increased, while IL10 levels slightly decreased, and no significant difference (one‐way ANOVA, *p* = 0.0536) in IL6 levels was observed (Figure 6J; Figure S52). These findings further support effective systemic immune activation and the resultant abscopal effect.

To further evaluate **in situ UMCOF1**‐associated immune activation and enhance tumor‐specific T‐cell responses, a tumor vaccine for mouse immunization was developed by dispersing whole‐cell lysates of 4T1 cells containing intracellularly synthesized **in situ UMCOF1** into hydrogels (Figure 6K). Hydrogels containing whole‐cell lysates from untreated 4T1 cells served as controls. NU/NU nude mice and BALB/c mice were vaccinated with the tumor vaccine once a week (Figure 6L). One week after the third vaccination, 4T1 cells were subcutaneously injected to evaluate vaccine efficacy. Analysis of the subcutaneously formed tumors after 5 weeks revealed that the tumor vaccine delayed the growth of 4T1 tumors in BALB/c mice, with 50 % of the mice showing no tumor formation (Figure 6M). Conversely, the tumor vaccine did not demonstrate immune efficacy in T cell‐deficient NU/NU nude mice, highlighting the essential role of T cell activation in preventing tumor formation. Additionally, inoculating BALB/c mice with the tumor vaccine hydrogel led to a 2.3 fold increase in T cells infiltrating the tumor compared to the control hydrogel group, further supporting the role of T cells in inhibiting tumor formation (Figure 6N; Figure S53). The tumor vaccine exhibited no systemic toxicity in either mouse strain, as indicated by stable body weight throughout the observation period (Figure S54).

## 3 Conclusion

We established an intracellular imine crystalline polymerization strategy for the in situ synthesis of COF nanoparticles. Owing to their fluorescent emission and crystalline structure, intracellular COFs can be directly tracked and characterized using fluorescence microscopy and TEM. The intracellular synthesis of COFs disrupts lysosomal homeostasis, activates lysosomal iron, and induces lipid peroxidation and ferroptotic cell death. This lysosomal membrane‐originated ferroptosis provides sustained release of DAMPs, representing an immunogenic form of cell death. Besides ferroptosis‐induced tumor regression, macrophages and DCs are activated to induce T cell differentiation, thereby enhancing antitumor immunotherapy. This intracellular crystalline polymerization strategy and its therapeutic applications provide a unique perspective for further research on cellular engineering and intracellular artificial chemical reactions.

COFs, as crystalline porous polymeric materials, have been commonly synthesized using solvothermal methods in organic solvents since their definition by Yaghi et al. in 2005 [70]. However, this approach requires relatively high temperatures, vacuum operation, and long reaction times, limiting morphological control and scaled synthesis [71, 72, 73, 74]. Recently, mild synthesis of COFs has been reported; however, these processes still rely on toxic organic solvents [75, 76, 77], bio‐unfriendly ionic surfactants [78, 79], and external energy sources [80, 81, 82]. A generalized method for synthesizing uniformly sized nanoscale COFs is currently lacking [83]. The present study demonstrates that various imine‐linked COF nanoparticles can be prepared in weakly acidic buffer solutions without the use of Pyrex ampoules, high temperatures, organic solvents, specialized catalysts, or strict oxygen‐free conditions. Using a non‐toxic non‐ionic surfactant, we achieved a mild and convenient synthesis of five different imine‐linked COFs in aqueous solution and extended the synthesis to cellular environments.

Ferroptosis, as defined by the Stockwell group in 2012 [84], is a non‐apoptotic cell death characterized by iron overload and uncontrolled lipid peroxidation. Its immunogenicity remains debatable [85, 86]. Various research groups have shown that ferroptosis induced by small‐molecule drugs [87, 88] and nanomaterials [89] can improve immune checkpoint blockade therapies. However, recent findings suggest that ferroptotic cells may inhibit DC maturation [90], and their signature metabolite PGE2 could facilitate immune evasion [91, 92]. Notably, immunogenic cell death depends not just on CALR exposure on the cell membrane and the release of DAMPs. The location and timing of lipid peroxidation play a crucial role in determining its immunogenicity. The exact mechanism of lysosomal ferroptosis and its impact on tumor immunogenicity require further elucidation [93, 94, 95, 96].

The present study still has limitations, as the proposed therapy lacks sufficient specificity for cancer cells. While intratumoral drug administration reduces the exposure of healthy cells to therapeutic concentrations — thereby somewhat mitigating systemic toxicity risks — we recognize that potential long‐term toxicity may arise from COF‐monomers escaping the tumor site and entering the systemic circulation. This represents a significant barrier to clinical translation. Future research directions could explore tumor‐specific targeting strategies, such as pH‐responsive nanocarriers or tumor‐selective ligands, to enhance specificity. Nevertheless, our approach of intracellular crystalline polymerization holds clinical promise through co‐injecting COF monomers rather than having to deliberately synthesize, isolate, and stabilize COF nanoparticles.

## 4 Experimental Section

### 4.1 Synthesis of **UMCOF1** in the Buffer Solution

DMTP (13.7 mg, 70.5 µmol) was added to a phosphate buffer (10 mM, pH 5.0, 20 mL) containing Tween‐80 (0.5 vol. %). TAPB (16.5 mg, 47.0 µmol) was also added to the phosphate buffer (10 mM, pH 5.0, 20 mL) containing Tween‐80 (0.5 vol. %). The two solutions were mixed with the phosphate buffer (10 mM, pH 5.0, 900 mL), followed by incubation at 37°C for 24 h. Triethylamine (10 mL) was subsequently introduced to the reaction mixture to quench the reaction. After 10 min, the precipitate was collected via centrifugation at 12 000 rpm for 30 min at 4°C and rinsed thrice with water and once with ethanol. Finally, the powder was dried using supercritical carbon dioxide to yield a solid yellow product. Yield: 18 mg (65 % based on C_78_H_60_N_6_O_6_).

### 4.2 Intracellular Synthesis of **In Situ UMCOF1**

TAPB and DMTP were dissolved in PBS containing Tween‐80 (0.5 vol. %) and prepared as stock solutions at concentrations of 4.0 and 6.0 mM, respectively. Prior to the experiments, the stock solutions were diluted to the desired concentrations in a culture medium and then incubated with the cells for the specified durations. The detailed protocols are provided in the Supporting Information.

### 4.3 Experimental Animals

BALB/c mice (4–5 weeks of age, female) and NU/NU nude mice (4–5 weeks of age, female) were acquired from Vital River (Beijing, China) and housed in a filter‐topped pathogen‐free facility. The mice were provided with ^60^Co‐irradiated food and autoclaved water and kept in a room with a 12:12 h light–dark cycle, a temperature between 20°C and 23°C, and 30 %–70 % relative humidity. All studies involving mice were approved by the Ethics Committee of Shandong Normal University (Jinan, China) under the approval number AEECSDNU2024001. Unilateral and bilateral subcutaneous 4T1 tumor models of the BALB/c mice were utilized to assess the direct antitumor and abscopal effects, respectively, resulting from the intratumoral synthesis of **in situ UMCOF1**. Furthermore, hydrogels containing whole‐cell lysates of TAPB‐ and DMTP‐treated 4T1 cells were subcutaneously injected once a week, three times into healthy BALB/c mice and NU/NU nude mice. Subsequently, the tumor cells were subcutaneously injected to evaluate the preventive effect of the tumor vaccine. Further details regarding the methods can be found in the Supporting Information.

### 4.4 Statistical Analysis

Data are presented as mean ± SD. The sample sizes were not predetermined using statistical methods but followed accepted conventions. No data were excluded from the analyses. The data distribution was assumed to be normal, but this was not formally tested. Variance alignment was verified using the Brown–Forsythe test. The experimental sample size and statistical analysis are described in the corresponding Figure legends. The researchers were not blinded to the experimental design or result assessments during most of the experiments since data analyses were based on objectively measurable data, but the immunophenotyping and histopathology experiments were performed in a blinded fashion.

## Funding

The authors acknowledge financial support from the Science and Technology Development Fund (FDCT) of Macau SAR (0047‐2023‐AMJ, 0070/2023/RIA2, and 0002/2025/NRP), University of Macau (MYRG‐CRG2024‐00039‐ICMS), the Innovation Support Program of Jiangsu Province (BZ2023055), and NJU International Fellowship Initiative. Q.G. and L.‐L.Z. are supported by the UM Macao Postdoctoral Fellowship program.

## Conflicts of Interest

The authors declare no conflicts of interest.

## Supporting information




**Supporting File 1**: adma72471‐sup‐0001‐SuppMat.pdf.

**Supporting File 2**: adma72471‐sup‐0002‐Data.zip.

## Data Availability

The data that support the findings of this study are available in the supplementary material of this article.
