# Supplementary material for: In Situ Synthesis of Covalent Organic Frameworks Inside Cells for Triggering Ferroptosis and Immunotherapy
Source: Adv Mater. 2026 Feb 17;38(16):e10663. doi: 10.1002/adma.202510663 (PMC12994315; doi:10.1002/adma.202510663)
Supplement: Supplementary file 1 — Supporting File 1: adma72471‐sup‐0001‐SuppMat.pdf. [file ADMA-38-e10663-s001.pdf]

1. Experimental materials

All chemicals were purchased from Macklin (Shanghai, China), TCI (Shanghai, China), Sinopharm (Beijing, China), and Sigma–Aldrich (Shanghai, China), unless other noted, and used without further purification. Small-molecule pharmacological inhibitors, unless other noted, were purchased from MedChemExpress (Shanghai, China).

2,5-Dimethoxyterephthalaldehyde (DMTP) and 2,5-dihydroxyterephthalaldehyde (DHTP) were purchased from LeYan (Shanghai, China). Benzene-1,3,5-tricarbaldehyde (TFB), 1,3,5-tris(4-aminophenyl)benzene (TAPB), 2,5-divinylterephthalaldehyde (DVTP), 4,4′,4″,4‴-methanetetrayltetraaniline (TAPM), and 4,4′,4″,4‴-(porphyrin-5,10,15,20-tetrayl)tetraaniline (TAPP) were purchased from Jilin Chinese Academy of Sciences – Yanshen Technology (Changchun, China). Highly purified TAPB is a white powder, and the grey or grey-black powder requires further purification.

2. Cell culture

HCT116 (human colon carcinoma), CT26 (mouse colon adenocarcinoma), and 4T1 (malignant neoplasms of the mouse mammary gland) cell lines were provided by the Cell Bank, Chinese Academy of Sciences (Shanghai, China). KYSE510 (human esophageal squamous cell carcinoma) and HEEC (human esophageal epithelia) cell lines were generous gifts from Dr. Wei Zhou at the Department of Radiation Oncology, Qilu Hospital of Shandong University (Jinan, China). HCT116, CT26, 4T1, KYSE510, HEEC cells were cultured in RPMI-1640 medium (Gibco, Cat# C22400500BT) supplemented with fetal bovine serum (10 vol%, Gibco, Cat# 10270106) and Normocin (100 μg/mL, Invivogen, Cat# ant-nr-2) in a water-jacketed incubator with CO_2 (5 vol%) at 37°C.

MCF7 (human invasive breast carcinoma) cell line was provided by Institute of Basic Medicine, Shandong Academy of Medical Sciences (Jinan, China), and cultured in DMEM (Gibco, Cat# C11995500BT) supplemented with HEPES (25 mM, pH 7.3, Vivacell, Cat# C3544-0100), fetal bovine serum (10 vol%, Gibco, Cat# 10270106), GlutaMAX (1×, Gibco, Cat# 35050061), human recombinant insulin (10 μg/mL, Sigma–Aldrich, Cat# I3536), and Normocin (100 μg/mL, Invivogen, Cat# ant-nr-2) in a water-jacketed incubator with CO_2 (5 vol%) at 37°C.

HT1080 (human fibrosarcoma) cell line was provided by the Cell Bank, Chinese Academy of Sciences (Shanghai, China), and cultured in MEM (Gibco, Cat# C11095500BT) supplemented with HEPES (25 mM, pH 7.3, Vivacell, Cat# C3544-0100), fetal bovine serum (10 vol%, Gibco, Cat# 10270106) and Normocin (100 μg/mL, Invivogen, Cat# ant-nr-2) in a water-jacketed incubator with CO_2 (5 vol%) at 37°C.

B16F10 (mouse melanoma) cell line was provided by the Cell Bank, Chinese Academy of Sciences (Shanghai, China). RAW264.7 (mouse macrophage cell derived from a tumour induced by Abelson murine leukemia virus) cell line was obtained from the American Type Culture Collection (Manassas, USA). B16F10 and RAW264.7 cells were cultured in DMEM (Gibco, Cat# C11995500BT) supplemented with HEPES (25 mM, pH 7.3, Vivacell, Cat# C3544-0100), fetal bovine serum (10 vol%, Gibco, Cat# 10270106), and Normocin (100 μg/mL, Invivogen, Cat# ant-nr-2) in a water-jacketed incubator with CO_2 (5 vol%) at 37°C.

MCF10A (human mammary epithelia) cell line was provided by the Stem Cell Bank, Chinese Academy of Sciences (Shanghai, China), and cultured using MEGM BulletKit (Lonza, Cat# CC-3150) supplemented with Normocin (100 μg/mL, Invivogen, Cat# ant-nr-2) in a water-jacketed incubator with CO_2 (5 vol%) at 37°C.

3. Experimental instrumentations

Single crystal X-ray diffractometry was performed on an Agilent SuperNova CCD-based diffractometer with Cu *K*α radiation (λ = 1.54184 Å).

Supercritical carbon dioxide drying was performed using a Tousimis Samdri-PVT-3D critical point dryer. Damp solids were contained in folded filter paper secured with a staple and dried with a 15 min purge time and 15 min equilibration time after heating.

Raman spectrum was obtained by a LabRAM HR Evolution Raman spectrometer with a 532 nm laser.

Powder X-ray diffraction (PXRD) patterns were obtained on a Rigaku SmartLab SE X-ray powder diffractometer with Cu $K\alpha$ line focused radiation (λ = 1.54184 Å) in the range of 2θ = 2.00°–50.00° (or 2.00°–5.00°) at a step size of 0.01° and a scanning rate of 5.00°/min (or 0.50°/min) in a continuous mode. Powder samples were placed directly on zero-background sample holders and flattened using glass microscope slides.

Inductively coupled plasma–mass spectrometry (ICP–MS) measurements were performed using a PerkinElmer NexION 300X ICP–MS.

Liquid-state ^{1}H and ^{13}C nuclear magnetic resonance (NMR) spectra were recorded on a Bruker Avance III 400 MHz NMR spectrometer. DMSO-d_6 (LeYan, Cat# C400002) was used as a solvent. Chemical shifts were reported as δ values relative to tetramethylsilane as an internal reference compound. The ^{1}H and ^{13}C NMR spectra averaged 16 and 1024 scans, respectively.

Cross-polarisation magic-angle spinning (MAS) solid-state ^{13}C NMR spectra were recorded on a Bruker Avance III HD 400 MHz NMR spectrometer with a 4 mm double-resonance MAS probe. Each spectrum represented an average of 1200 scans.

Fourier transform infrared (FTIR) spectra were obtained on a Thermo Scientific Nicolet iS50 FTIR spectrometer equipped with a diamond attenuated total reflection (ATR) module between 4000–400 cm^{-1}. Each spectrum represented an average of 128 scans.

Nitrogen adsorption–desorption isotherms were collected by a Micromeritics ASAP 2460 surface area and porosity analyzer at 77K. The samples were degassed at 120°C for 8 h using a VacPrep degassing station before measurement. The Brunauer–Emmett–Teller (BET) equation was used to calculate the specific surface areas. The pore size distribution was derived using the non-local density functional theory model.

3D fluorescence spectra were recorded using a Hitachi F-7000 fluorescence spectrophotometer equipped with a 10 mm quartz cuvette at a scan rate of 2400 nm/min. 2D fluorescence spectra were recorded using a Hitachi F-2710 fluorescence spectrophotometer equipped with a 10 mm quartz cuvette at a scan rate of 300 nm/min. The widths of the excitation and emission slits were both set to 2.5 nm.

Time-resolved photoluminescence spectra were measured using a Hamamatsu C11367 Quantaurus-Tau fluorescence lifetime spectrometer with a Hamamatsu M12977 time-to-digital converter unit.

High-resolution mass spectrum (HRMS) was reported as required on the four instruments described below: (i) Bruker maXis ultrahigh-resolution time-of-flight triple quadrupole mass spectrometer equipped with an electron spray ionisation (ESI) source; (ii) Agilent 7250 GC/Q-TOF quadrupole time-of-flight mass spectrometer equipped with a low-energy electron ionization (EI) source; (iii) Shimadzu LCMS-IT-TOF ion trap time-of-flight liquid chromatograph–mass spectrometer. (iv) AB SCIEX TripleTOF 5600+ system. HRMS was reported as mass per charge ratios (m/z).

Ultraviolet–visible (UV–vis) absorption spectra were recorded as required on the three instruments described below: (i) Shimadzu UV-2700 double-beam UV–vis spectrophotometer equipped with a 10 mm quartz cuvette; (ii) Thermo Scientific NanoDrop One microvolume UV–vis spectrophotometer; (iii) Molecular Devices SpectraMax M2 multimode microplate reader equipped with a temperature control system.

Thermogravimetric analysis (TGA) was performed with a Mettler Toledo TGA/DSC3+ thermogravimetric analyzer. Approximately 4.0 mg of dried sample was analysed from room temperature to 1000°C at a heating rate of 10°C/min under a N_2 atmosphere.

Microplate assays were conducted using Molecular Devices SpectraMax iD5 and M2 multimode microplate readers.

Western blot images were obtained using GE Healthcare Amersham Imager 600 and 680 devices.

Scanning electron microscopy (SEM) images were recorded using a Zeiss Supra 55 instrument. The SEM samples were prepared by depositing a diluted suspension onto silicon wafers (approximately 3 × 3 mm), followed by air drying and coating with a thin layer of Pt to increase the contrast.

Elemental analyses were performed on an Elementar Vario EL Cube CHNS elemental analyzer.

Transmission electron microscopy (TEM) images were recorded on a Hitachi HT7700 120 kV compact-digital instrument. High-resolution TEM images were recorded on an FEI Tecnai G2 F20 high-resolution scanning transmission electron microscope. The samples were prepared in methanol by sonicating the material for 5 min, followed by application on a copper TEM grid, and air-dried at room temperature.

Flow cytometric analysis was performed using the following two devices as appropriate: (i) BD FACSAria Fusion cell sorter equipped with 405, 488, 561, and 640 nm lasers; (ii) Beckman Coulter CytoFLEX flow cytometer equipped with 405, 488, 638 nm lasers. For live cell analysis, unless otherwise noted, cells were maintained in HBSS supplemented with HEPES (25 mM, pH 7.3, Vivacell, Cat# C3544-0100), GlutaMAX (1×, Gibco, Cat# 35050061), and fetal bovine serum (2.0 vol%, Gibco, Cat# 10270106). For fixed cell analysis, unless otherwise noted, cells were maintained in PBS. For immunophenotyping, cells were maintained in flow cytometry staining buffer (Invitrogen, Cat# 00-4222-26). BD FACSuite CS&T research beads (BD Biosciences, Cat# 650622) were used for performance (daily) and characterization (every 6 months) quality controls to ensure the BD instrument reliability.

Hydrodynamic particle sizes of nanoparticles were measured using a Malvern Zetasizer Nano ZS90 system.

Bright-field imaging was conducted using the following three devices as appropriate: (i) Leica DMi8 inverted microscope; (ii) Olympus IX73 inverted microscope; (iii) Sartorius Incucyte S3 live-cell imaging system placed in a water-jacketed CO_2 incubator with CO_2 (5 vol%) at 37°C.

The reverse transcription reaction and quantitative real-time polymerase chain reaction (RT-qPCR) were performed on a Bio-Rad CFX Connect system.

Laser scanning confocal fluorescence images of cells were captured with a Leica Stellaris 5 confocal laser scanning microscope equipped with 405 nm and supercontinuum white light lasers using the 10×, 20×, and 63× objective lens at 23°C. The imaging scan speed was 400 Hz, and transmitted light was used to find the areas of interest to reduce photodamage to the sample. Four-well glass-bottom dishes (Cellvis, Cat#D35C4-20-0-N) and eight-chamber slides (Cellvis, Cat#C8-1.5H-N) were used for cell culture in experiments. For live cell imaging, cells were maintained in FluoroBrite DMEM (Gibco, Cat# A1896701) supplemented with HEPES (25 mM, pH 7.3, Vivacell, Cat# C3544-0100). For fixed cell imaging, cells were maintained in PBS. Multiple controls were conducted as needed to avoid nonspecific staining and crosstalk between channels.

4. Synthesis of (1*E*,1'*E*)-1,1'-(2,5-dimethoxy-1,4-phenylene)bis(*N*-phenylmethanimine)

DMTP (582.6 mg, 3.0 mmol), aniline (1.1 mL, 12 mmol), and acetic acid (10 mL) were added in water (400 mL) containing Tween-80 (0.5 vol%). The mixture was refluxed for 72 h and filtered with Nylon membranes while hot. The filtrate was cooled to room temperature, extracted with dichloromethane, and recrystallised in ethanol to obtain yellow crystals. Yield: 469 mg (45%, based on $C_{22}H_{20}N_2O_2$). ^{1}H NMR (400 MHz, DMSO-d_6) δ 8.87 (s, 2H), 7.74 (s, 2H), 7.47–7.40 (m, 4H), 7.31–7.25 (m, 6H), 3.94 (s, 6H). ^{13}C NMR (101 MHz, DMSO-d_6) δ 155.41, 153.90, 152.21, 129.80, 127.98, 126.76, 121.50, 109.87, 56.70. HRMS (ESI) *m/z* calculated for $C_{22}H_{21}N_2O_2$ $[M+H]^+$: 345.15975; found: 345.15717. Elemental analysis calculated for $C_{22}H_{20}N_2O_2$: C 76.50, H 6.13, N 8.11; found: C 76.22, H 6.07, N 7.89. ATR–FTIR ν/cm^{-1} 3058 (w), 3026 (w), 2968 (w), 2927 (w), 2830 (w), 1615 (m), 1586 (m), 1481 (m), 1459 (m), 1446 (m), 1405 (m), 1369 (s), 1294 (m), 1202 (s), 1139 (m), 1072 (w), 1032 (s), 970 (m), 927 (w), 876 (s), 832 (m), 757 (s), 688 (s), 599 (m), 525 (s), 498 (m), 440 (s), 412 (w).

A suitable single crystal was selected and mounted onto thin glass fibers. Crystal data were collected on an Agilent SuperNova

CCD-based diffractometer (Cu *Kα* radiation, λ = 1.54184 Å) at 208 K. The structure was solved by a combination of direct methods with subsequent difference Fourier syntheses and refined by a full matrix least-squares technique based on F^2 using the SHELXL software package. All the non-hydrogen atoms were refined anisotropically. The hydrogen atoms were generated geometrically. Crystallographic data for the structure reported herein have been deposited with the Cambridge Crystallographic Data Centre (CCDC) with a reference number of CCDC 2361348.

5. Synthesis of UMCOF2–5 in buffer solution

For synthesis of **UMCOF2**, DVTP (11.2 mg, 0.06 mmol) was added in a phosphate buffer (10 mM, pH 5.0, 20 mL) containing Tween-80 (0.5 vol%). TAPB (14.1 mg, 0.04 mmol) was added in a phosphate buffer (10 mM, pH 5.0, 20 mL) containing Tween-80 (0.5 vol%). The two solutions were mixed with a phosphate buffer (10 mM, pH 5.0, 900 mL) followed by incubation at 37°C for 24 h. After that, triethylamine (10 mL) was added to the reaction system to quench the reaction. After 10 min, the precipitate was collected *via* centrifugation at 12000 rpm for 30 min at 4°C and washed thrice with water and once with ethanol. Finally, the powders were dried in supercritical carbon dioxide to obtain the yellow solid product. Yield: 11 mg (48%, based on $C_{84}H_{60}N_6$).

For synthesis of **UMCOF3**, TFB (13.0 mg, 0.08 mmol) was added in a phosphate buffer (10 mM, pH 5.0, 20 mL) containing Tween-80 (0.5 vol%). TAPB (28.1 mg, 0.08 mmol) was added in a phosphate buffer (10 mM, pH 5.0, 20 mL) containing Tween-80 (0.5 vol%). The two solutions were mixed with a phosphate buffer (10 mM, pH 5.0, 900 mL) followed by incubation at 37°C for 24 h. After that, triethylamine (10 mL) was added to the reaction system to quench the reaction. After 10 min, the precipitate was collected *via* centrifugation at 12000 rpm for 30 min at 4°C and washed thrice with water and once with ethanol. Finally, the powders were dried in supercritical carbon dioxide to obtain the milk white solid product. Yield: 22 mg (60%, based on $C_{33}H_{21}N_3$).

For synthesis of **UMCOF4**, DHTP (13.3 mg, 0.08 mmol) was added in a phosphate buffer (10 mM, pH 5.0, 20 mL) containing Tween-80 (0.5 vol%). TAPP (27.0 mg, 0.04 mmol) was added in a phosphate buffer (10 mM, pH 5.0, 20 mL) containing Tween-80 (0.5 vol%). The two solutions were mixed with a phosphate buffer (10 mM, pH 5.0, 900 mL) followed by stirring at 600 rpm and 37°C for 72 h. After that, the precipitate was collected *via* centrifugation at 12000 rpm for 30 min at 4°C and washed thrice with DMF and twice with ethanol. Finally, the powders were dried in supercritical carbon dioxide to obtain the deep purple solid product. Yield: 10 mg (27%, based on $C_{60}H_{38}N_8O_4$).

For synthesis of **UMCOF5**, PDA (32.2 mg, 0.24 mmol) was added in an acetate buffer (10 mM, pH 5.0, 20 mL) containing Tween-80 (0.5 vol%). TAPM (45.7 mg, 0.12 mmol) was added in an acetate buffer (10 mM, pH 5.0, 20 mL) containing Tween-80 (0.5 vol%). The two solutions were mixed with an acetate buffer (10 mM, pH 5.0, 900 mL) followed by stirring at 600 rpm and 60°C for 7 d. After that, the precipitate was collected *via* centrifugation at 12000 rpm for 30 min at 4°C and washed thrice with water and once with ethanol. Finally, the powders were dried in supercritical carbon dioxide to obtain the yellow solid product. Yield: 46 mg (66%, based on $C_{164}H_{112}N_{16}$).

6. Structural modeling and geometry optimisation

Molecular modeling and Pawley refinement were conducted using *BIOVIA Materials Studio 2018*. The initial lattice model was conceived from the native connectivity and geometry of the building blocks and subsequently geometrically optimised using the Forcite molecular dynamics module (Universal force fields, Ewald summations). The lattice parameters were further optimised by Pawley refinement using the Reflex package, in conjunction with the experimental PXRD patterns and optimised COF structures, until the residual precision (R_p) and the weighted residual precision (R_{wp}) values converged.

7. Cellular uptake of organic monomers

To evaluate the cellular uptake of TAPB and DMTP, 4T1 cells cultured in T225 flasks were treated with RPMI-1640 medium (40 mL) containing TAPB (50 μM) or DMTP (75 μM) for 24 h. The cells were collected with a cell scraper, washed three times with

normal saline, and collected by centrifugation at 1200 rpm for 10 min. The collected cells were homogenised in a mixture of methanol (800 μL, precooled at −80°C) and water (320 μL, precooled at 4°C). The lysates were then mixed with chloroform (800 μL, precooled at −20°C), sonicated for 5 min, and centrifuged at 13000 rpm for 15 min at 4°C. The bottom liquids were transferred to 1.5 mL centrifuge tubes, concentrated in a nitrogen flow, and stored at −20°C until analysis by HRMS.

To evaluate the membrane permeability of a highly charged fluorescent dye by confocal laser scanning fluorescence microscope, 4T1 cells cultured in 4-chamber glass-bottom dishes were pretreated with PBS (400 μL) or PFA (4 wt%, 400 μL) for 30 min and washed twice with PBS. Then, the cells were treated with TO-PRO-3 (1.0 μM, 400 μL, Invitrogen, Cat# T3605) supplemented with Tween-80 (0.02 vol%) for 30 min. The cells were washed twice with PBS, and confocal laser scanning fluorescence images were captured. The red images of TO-PRO-3 were recorded at an excitation wavelength of 633 nm and an emission wavelength range of 650–700 nm. Untreated cells were used as the control group.

8. Flow cytometric analysis of intracellularly synthesised COF nanoparticles

For assessing intracellular synthesis of **in-situ UMCOF1** by flow cytometry, 4T1 cells cultured in 6-well plates were pretreated with DMSO (0.1 vol%, 2.0 mL) or BafA1 (100 nM, 2.0 mL) for 8 h and washed twice with PBS. Then the cells were treated with RPMI-1640 medium (2.0 mL) containing TAPB (50 μM) and DMTP (75 μM) for 0–24 h. The cells were harvested using a trypsin–EDTA solution, strained through a 40 μm cell strainer, and analysed using a flow cytometer equipped with a 405 nm laser for excitation and a KO525 channel for collection.

For assessing the possibility of extracellular polymerisation reactions, RPMI-1640 medium containing TAPB (100 μM) and DMTP (150 μM) was incubated at 37°C for 0–7 days, followed by centrifugation at 12000 rpm for 30 min. Subsequently, the supernatant was diluted with RPMI-1640 medium and incubated with 4T1 cells cultured in 6-well plates for 6 h. The cells were harvested using a trypsin–EDTA solution, strained through a 40 μm cell strainer, and analysed using a flow cytometer equipped with a 405 nm laser for excitation and a KO525 channel for collection.

9. Confocal laser scanning fluorescence imaging of intracellularly synthesised COF nanoparticles

For confocal laser scanning fluorescence imaging of intracellularly synthesised **in-situ UMCOF1** nanoparticles, 4T1 cells cultured in 4-chamber glass-bottom dishes were pretreated with DMSO (0.1 vol%, 400 μL) or BafA1 (100 nM, 400 μL) for 8 h and washed twice with PBS. Then the cells were treated with RPMI-1640 medium (400 μL) containing TAPB (20 μM) and DMTP (30 μM) for 6 h. The cells were washed twice with PBS, incubated with LysoTracker Red DND-99 (50 nM, 200 μL, Invitrogen, Cat# L7528) for 30 min in a CO_2 incubator. The cells were washed twice with PBS, incubated with Nuclear Red LCS1 (2.0 μM, 200 μL, AAT Bioquest, Cat# 17542) for 10 min in a CO_2 incubator. The cells were washed twice with PBS, and confocal laser scanning fluorescence images were captured. The green images of **in-situ UMCOF1** were recorded at an excitation wavelength of 405 nm and an emission wavelength range of 500–540 nm. The red images of LysoTracker Red DND-99 were recorded at an excitation wavelength of 561 nm and an emission wavelength range of 580–620 nm. The cyan images of Nuclear Red LCS1 were recorded at an excitation wavelength of 633 nm and an emission wavelength range of 650–700 nm. Untreated cells were used as the control group.

In addition, a similar procedure was performed to assess **in-situ UMCOF1** synthesis in CT26, MCF7, HCT116, HT1080, KYSE510, B16F10, MCF10A, and HEEC cells, except that the RPMI-1640 medium was replaced by the appropriate medium containing TAPB (20 μM for HCT116, HT1080, and B16F10 cells; 50 μM for CT26, MCF7, KYSE510, and HEEC cells; 100 μM for MCF10A cells) and DMTP (30 μM for HCT116, HT1080, and B16F10 cells; 75 μM for CT26, MCF7, KYSE510, and HEEC cells; 150 μM for MCF10A cells).

10. Bio-TEM imaging of intracellularly synthesised COF nanoparticles

For bio-TEM imaging of intracellularly synthesised **in-situ UMCOF1** nanoparticles, 4T1 cells cultured in T75 flasks were treated with RPMI-1640 medium (10 mL) containing TAPB (50 μM) and DMTP (75 μM) for 24 h. The cells were carefully washed once

with PBS, fixed in glutaraldehyde (2.5 vol%, 10 mL) for 5 min, and collected with a cell scraper. The cells were collected *via* centrifugation at 1000 rpm for 10 min at 4°C, resuspended in glutaraldehyde (2.5 vol%, 1.0 mL), and stored at 4°C. On the analysis day, the cells were collected by centrifugation at 1000 rpm for 10 min at 4°C, dehydrated in a graded ethanol series, and embedded in an epoxy resin. All samples were cut into 50–80 nm thick sections for bio-TEM examination.

11. PXRD and fluorescence analyses of intracellularly synthesised COF nanoparticles

To isolate **in-situ UMCOF1** from 4T1 cells, 4T1 cells cultured in T225 flasks were pretreated with DMSO (0.1 vol%, 30 mL) or BafA1 (100 nM, 30 mL) for 8 h and washed twice with PBS. Then, the cells were treated with RPMI-1640 medium (30 mL) containing TAPB (50 μM) and DMTP (75 μM) for 24 h. The cells were washed once with PBS and harvested using a trypsin–EDTA solution. The cells were washed once with PBS and then lysed in a lysis buffer containing NaCl (150 mM), Triton X-100 (1.0 vol%), and HEPES (25 mM, pH 7.3, Vivacell, Cat# C3544-0100) under the assistance of ultrasound at 4°C. The lysates were centrifuged at 13000 rpm for 15 min at 4°C, and the supernatants were isolated to exclude cell debris and gelatinous substances. The supernatants were mixed with an equal volume of dichloromethane, sonicated for 5 min, and centrifuged at 13000 rpm for 15 min at 4°C to remove precipitated proteins. Subsequently, 1/5 volume of triethylamine was added to the resulting supernatants, followed by incubation at 40°C for 2 h. The resulting precipitates were washed once with water and twice with ethanol. To prepare the PXRD samples, the precipitates were redispersed in ethanol, deposited onto silicon wafers (approximately 3 × 3 mm), and air-dried. To measure the fluorescence spectra, the precipitates were redispersed in PBS.

12. Cell viability assays

For lysosomal acidification-related cell viability assays and their rescue experiments, 4T1 cells cultured in 96-well plates were pretreated with DMSO (0.1 vol%, 100 μL) or BafA1 (100 nM, 100 μL) for 8 h and washed twice with PBS. Then, the cells were treated with RPMI-1640 medium (100 μL) containing TAPB (0–200 μM) and DMTP (0–300 μM) for 24 h. Subsequently, the cells were washed three times with PBS. RPMI-1640 medium (90 μL) and CCK-8 solution (10 μL, Dojindo, Cat# CK04) were added to each well, and the plate was incubated in a CO_2 incubator for approximately 1 h. The absorbance at 450 nm was measured using a multimode microplate detection system. Untreated cells were used as the control group while methanol-treated cells were used as the blank. Furthermore, a similar procedure was conducted to assess cell viabilities of CT26, MCF7, HCT116, HT1080, KYSE510, B16F10, MCF10A, and HEEC cells, only replacing the RPMI-1640 medium with the appropriate medium.

For ferroptosis-related cell viability assays and their rescue experiments, 4T1 cells cultured in 96-well plates were treated with RPMI-1640 medium (100 μL) containing TAPB (50 μM) and DMTP (75 μM) in the absence or presence of DMSO (0.1 vol%), Fer-1 (1.0 μM), Lip-1 (0.5 μM), NAC (5.0 mM), GSHee (2.0 mM), Trolox (100 μM), VitC (100 μM), BAPTA-AM (1.0 μM), DFOM (100 μM), $(NH_4)_2MoS_4$ (10 μM), Nec-1s (5.0 μM), necrosulfonamide (1.0 μM), VX765 (20 μM), Ac-FLTD-CMK (10 μM), and zVAD (25 μM) for 24 h. Subsequently, the cells were washed three times with PBS, and their cell viabilities were detected according to the above method. Cells treated with RSL3 (0.5 μM) for 24 h were used as the positive control of ferroptosis. Cells treated with CPT (2.0 μM) for 24 h were used as the positive control of apoptosis.

For cell viability assays of TAPB, DMTP, and pre-synthesised **UMCOF1** nanoparticles, 4T1 cells cultured in 96-well plates were treated with RPMI-1640 medium (100 μL) containing TAPB (0–500 μM), DMTP (0–500 μM), or pre-synthesised **UMCOF1** (1.0 mg/mL) for 24 h. Subsequently, the cells were washed three times with PBS. RPMI-1640 medium (90 μL) and CCK-8 solution (10 μL, Dojindo, Cat# CK04) were added to each well, and the plate was incubated in a CO_2 incubator for approximately 1 h. The absorbance at 450 nm was measured using a multimode microplate detection system. Untreated cells were used as the control group while methanol-treated cells were used as the blank.

The cell viability data were fitted to a logistic equation and iterated to convergence using the orthogonal distance regression algorithm.

13. Cytotoxicity assays

For assessing cytotoxicity of TAPB, DMTP, and pre-synthesised **UMCOF1** nanoparticles by flow cytometry, 4T1 cells cultured in 12-well plates were treated with RPMI-1640 medium (1.0 mL) containing TAPB (50 μM), DMTP (75 μM), or pre-synthesised **UMCOF1** (200 μg/mL) for 24 h. The cells were harvested using a trypsin–EDTA solution, mixed with 7-AAD (5.0 μM, 400 μL), and analysed using a flow cytometer equipped with a 488 nm laser for excitation and a PC5.5 channel for collection. Cells treated with PFA (4 wt%,1.0 mL) for 10 min at 25°C were used as the positive control. Untreated cells were used as the negative control.

For assessing cytotoxicity of TAPB, DMTP, and pre-synthesised **UMCOF1** nanoparticles by confocal laser scanning fluorescence microscopy, 4T1 cells cultured in 4-chamber glass-bottom dishes were treated with RPMI-1640 medium (400 μL) containing TAPB (50 μM), DMTP (75 μM), or pre-synthesised **UMCOF1** (200 μg/mL) for 24 h. The cells were stained with RPMI-1640 medium (200 μL) containing Calcein Deep Red AM ester (5.0 μM, AAT Bioquest, Cat# 22011) and PI (5.0 μM) for 30 min in a CO_2 incubator. The cells were washed twice with PBS for capturing confocal laser scanning fluorescence images. The green images of Calcein Deep Red AM ester were recorded at an excitation wavelength of 633 nm and an emission wavelength range of 640–680 nm. The red images of PI were recorded at an excitation wavelength of 561 nm and an emission wavelength range of 570–610 nm. Cells treated with PFA (4 wt%) for 24 h at 4°C were used as the positive control. Untreated cells were used as the negative control.

14. Synthesis of in-situ UMCOF1 in 3D multicellular tumour spheroids

For synthesizing and imaging **in-situ UMCOF1** in 4T1 multicellular tumour spheroids, multicellular tumour spheroids with a size of approximately 600 μm were treated with RPMI-1640 medium (200 μL) containing TAPB (20 μM), DMTP (30 μM), and Nuclear Red LCS1 (1.0 μM, AAT Bioquest, Cat# 17542) for 4 h in a CO_2 incubator. The spheroids were washed twice with PBS and carefully transferred to a glass-bottom dish for confocal laser scanning tomographic fluorescence imaging with a slice thickness of 10 μm. The red images of Nuclear Red LCS1 were recorded with an excitation wavelength of 633 nm and an emission wavelength range of 650–700 nm. The green images of **in-situ UMCOF1** were recorded at an excitation wavelength of 405 nm and an emission wavelength range of 500–540 nm.

For imaging cell death in 4T1 multicellular tumour spheroids, multicellular tumour spheroids with a size of approximately 600 μm were treated with RPMI-1640 medium (200 μL) containing TAPB (100 μM), DMTP (150 μM), and PI (5.0 μM) for 0–72 h in a CO_2 incubator. Red fluorescent and bright-field images were captured at different times.

For analyzing cell death using flow cytometry in 4T1 multicellular tumour spheroids, multicellular tumour spheroids with a size of approximately 600 μm were treated with RPMI-1640 medium (200 μL) containing TAPB (100 μM) and DMTP (150 μM) for 24 h in a CO_2 incubator. The spheroids were harvested using a trypsin–EDTA solution, washed twice with PBS, and resuspended in Calcein Deep Red AM ester (5.0 μM, 400 μL, AAT Bioquest, Cat# 22011). After 30 min of incubation in a CO_2 incubator, the cells were washed twice with PBS, and mixed with PI (5.0 μM, 400 μL) for flow cytometric analysis. The signals of PI and Calcein Deep Red AM ester were excited by 561 nm and 640 nm lasers, and collected in PI and APC channels, respectively.

Additionally, synthesis of **in-situ UMCOF1** was performed in CT26, MCF7, HCT116, HT1080, and KYSE510 multicellular tumour spheroids using similar protocols. For confocal laser scanning fluorescence imaging, the focal plane was placed at the maximum diameter of the spheroids. For flow cytometric analysis, the dissociated spheroids were mixed with 7-AAD (5.0 μM, 400 μL) to stain dead cells. The signals of 7-AAD were excited by a 488 nm laser and collected in a PerCP-Cy5.5 channel.

15. Clonogenic assays

4T1 cells were cultured at a density of 500 cells/well in 12-well plates for 24 h, treated with RPMI-1640 medium (100 μL) containing TAPB (50 μM) and DMTP (75 μM) in the absence or presence of DMSO (0.1 vol%), BafA1 (50 nM), Fer-1 (1.0 μM), Lip-

1 (0.5 μM), NAC (5.0 mM), GSHee (2.0 mM), Trolox (100 μM), VitC (100 μM), BAPTA-AM (1.0 μM), DFOM (100 μM), $(NH_4)_2MoS_4$ (10 μM), Nec-1s (5.0 μM), necrosulfonamide (1.0 μM), VX765 (20 μM), Ac-FLTD-CMK (10 μM), and zVAD (25 μM) for 24 h. After 5 days of incubation, the cells were fixed with PFA (4 wt%, 2.0 mL) for 24 h at 4°C and stained with fresh Giemsa staining solution for 12 h at room temperature. The plates were washed with water, air-dried, and photographed with a digital camera. The untreated well was used as the negative control group. Cells treated with CPT (2.0 μM) for 24 h were used as the positive control of apoptosis.

CT26, MCF7, HCT116, HT1080, and KYSE510 cells were cultured at a density of 10^3 cells/well in 6-well plates for 24 h, treated with RPMI-1640 medium (100 μL) containing TAPB (100 μM) and DMTP (150 μM) for 24 h. After 5 days of incubation, the cells were fixed with PFA (4 wt%, 2.0 mL) for 24 h at 4°C and stained with fresh Giemsa staining solution for 12 h at room temperature. The plates were washed with water, air-dried, and photographed with a digital camera. The untreated well was used as the control group.

16. Lysosomal alkalinisation and membrane permeability assays

For assessing lysosomal alkalisation of 4T1 cells by flow cytometry, 4T1 cells cultured in 12-well plates were treated with RPMI-1640 medium (1.0 mL) containing TAPB (50 μM) and DMTP (75 μM) for 24 h. The cells were harvested using a trypsin–EDTA solution, washed twice with PBS, and resuspended in LysoTracker Deep Red (50 nM, 1.0 mL, Invitrogen, Cat# L12492). After 30 min of incubation in a CO_2 incubator, the cells were washed twice with PBS, and mixed with 7-AAD (5.0 μM, 400 μL) for flow cytometric analysis. The signals of 7-AAD and LysoTracker Deep Red were excited by 488 nm and 638 nm lasers, and collected in PC5.5 and APC channels, respectively.

For assessing lysosomal membrane permeability of 4T1 cells by confocal laser scanning fluorescence microscopy, 4T1 cells cultured in 4-chamber glass-bottom dishes were treated with RPMI-1640 medium (400 μL) containing TAPB (50 μM) and DMTP (75 μM) for 24 h. The cells were washed twice with PBS and incubated with acridine orange hydrochloride (20 μM, 400 μL) for 10 min in a CO_2 incubator. The cells were washed twice with PBS for capturing confocal laser scanning fluorescence images. The green images of unprotonated acridine orange were recorded at an excitation wavelength of 488 nm and an emission wavelength range of 510–550 nm. The red images of protonated acridine orange were recorded at an excitation wavelength of 488 nm and an emission wavelength range of 620–660 nm. Untreated cells were used as the control group.

17. Intracellular metal ion assays

For detecting Fe^{2+} in 4T1 cells by confocal laser scanning fluorescence microscopy, 4T1 cells cultured in 4-chamber glass-bottom dishes were treated with RPMI-1640 medium (400 μL) containing TAPB (50 μM) or/and DMTP (75 μM) in the absence or presence of DFOM (100 μM) for 24 h. The cells were washed twice with PBS, incubated with Nuclear Red LCS1 (2.0 μM, 200 μL, AAT Bioquest, Cat# 17542) for 10 min in a CO_2 incubator. The cells were washed twice with PBS, incubated with FerroOrange (1.0 μM, 200 μL, Dojindo, Cat# F374) for 30 min in a CO_2 incubator. Confocal laser scanning fluorescence microscopy was performed on the unwashed cells. The yellow images of FerroOrange were recorded at an excitation wavelength of 543 nm and an emission wavelength range of 560–600 nm. The cyan images of Nuclear Red LCS1 were recorded at an excitation wavelength of 633 nm and an emission wavelength range of 650–700 nm. Untreated cells were used as the control group.

For detecting Cu in 4T1 cells by ICP–MS, 4T1 cells cultured in T225 flasks were treated with RPMI-1640 medium (30 mL) containing TAPB (50 μM) and DMTP (75 μM) for 24 h. The cells were washed twice with PBS, collected with a cell scraper, and digested in aqua regia at approximately 50°C. The mixture was diluted with nitric acid (1.0 wt%) for detecting Cu contents by ICP–MS. Untreated cells were used as the control group.

For detecting Ca^{2+} in 4T1 cells by flow cytometry, 4T1 cells cultured in 12-well plates were treated with RPMI-1640 medium (1.0 mL) containing TAPB (50 μM) and DMTP (75 μM) for 24 h. The cells were harvested using a trypsin–EDTA solution, washed

once with PBS, and incubated with Calbryte 630 AM (5.0 μM, 1.0 mL, AAT Bioquest, Cat# 20720) for 1 h in a CO_2 incubator. The cells were washed once with PBS, strained through a 40 μm cell strainer, and analysed using a flow cytometer equipped with a 638 nm laser for excitation and an APC channel for collection.

18. Lipid peroxidation and total ROS detections

For detecting lipid peroxidation of 4T1 cells by confocal laser scanning fluorescence microscopy, 4T1 cells cultured in 4-chamber glass-bottom dishes were treated with RPMI-1640 medium (400 μL) containing TAPB (50 μM) and DMTP (75 μM) in the absence or presence of DMSO (0.1 vol%), Fer-1 (1.0 μM), and DFOM (100 μM) for 24 h. The cells were washed twice with PBS, incubated with BODIPY$^{665/676}$ (5.0 μM, 200 μL, Invitrogen, Cat# B3932) for 30 min in a CO_2 incubator and washed twice with PBS. Laser scanning confocal fluorescence images were captured. The green images of non-oxidised BODIPY$^{665/676}$ were recorded at an excitation wavelength of 633 nm and an emission wavelength range of 660–700 nm. The red images of oxidised BODIPY$^{665/676}$ were recorded at an excitation wavelength of 561 nm and an emission wavelength range of 575–625 nm. Untreated cells were used as the negative control group. Cells treated with RSL3 (0.5 μM) for 24 h were used as the positive control.

For detecting lipid peroxidation of B16F10 cells by flow cytometry, B16F10 cells cultured in 6-well plates were treated with DMEM (2.0 mL) containing TAPB (50 μM) and DMTP (75 μM) in the absence of DMSO (0.1 vol%) or Lip-1 (0.5 μM) for 24 h. The cells were harvested using a trypsin–EDTA solution, washed once with PBS, and incubated with Liperfluo (1.0 μM, 1.0 mL, Dojindo, Cat# L248) for 30 min in a CO_2 incubator. The cells were washed twice with PBS, strained through a 70 μm cell strainer, and analysed using a flow cytometer equipped with a 488 nm laser for excitation and an FITC channel for collection.

For detecting intracellular total ROS in 4T1 cells by flow cytometry, 4T1 cells cultured in 12-well plates were treated with RPMI-1640 medium (1.0 mL) containing TAPB (50 μM) and DMTP (75 μM) for 24 h. The cells were harvested using a trypsin–EDTA solution, washed once with PBS, and incubated with CellROX Deep Red (5.0 μM, 500 μL, Invitrogen, Cat# C10422) for 30 min in a CO_2 incubator. The cells were washed once with PBS and analysed using a flow cytometer equipped with a 638 nm laser for excitation and an APC channel for collection. Untreated cells were used as the negative control group. Cells treated with RSL3 (0.5 μM) for 24 h were used as the positive control.

For detecting intracellular total ROS in RAW264.7 macrophages by flow cytometry, 4T1 cells cultured in 6-well plates were pretreated with DMSO (0.1 vol%, 2.0 mL) or Fer-1 (1.0 μM, 2.0 mL) for 24 h and treated with RSL3 (0.5 μM, 2.0 mL) or RPMI-1640 medium (2.0 mL) containing TAPB (50 μM) and DMTP (75 μM) for 4 h. The cells were washed twice with PBS and mixed with fresh RPMI-1640 medium. After an additional 20 h of incubation, the medium was mixed with an equal volume of DMEM for incubating RAW264.7 macrophages cultured in 12-well plates. After 24 h of incubation, the macrophages were collected by centrifugation at 1500 rpm for 10 min at 4°C, washed once with PBS, and loaded with DCFH-DA (20 μM, 500 μL, MedChemExpress, Cat# HY-D0940) for 30 min in a CO_2 incubator. The cells were washed once with PBS and analysed using a flow cytometer equipped with a 488 nm laser for excitation and an FITC channel for collection. RAW264.7 macrophages treated with LPS (100 ng/mL, 24 h, Sigma–Aldrich, Cat# L4391) were used as the positive control of classical activation. RAW264.7 macrophages treated with the medium of untreated 4T1 cells were used as the negative control group.

19. Intracellular GSH, MDA, and PGE2 assays

4T1 cells cultured in 10 cm culture dishes were treated with RPMI-1640 medium (10 mL) containing TAPB (50 μM) and DMTP (75 μM) in the absence or presence of DMSO (0.1 vol%), Fer-1 (1.0 μM), and DFOM (100 μM) for 24 h. The cells were carefully rinsed with PBS and used for measuring GSH, MDA, and PGE2 levels using the GSH assay kit (Nanjing Jiancheng Bioengineering Institute, Cat# A006-2-1), MDA assay kit (Nanjing Jiancheng Bioengineering Institute, Cat# A003-2-2), and PGE2 ELISA kit (Cayman, Cat# 514010) following the manufacturer's guidelines, respectively. The GSH, MDA, and PGE2 contents were normalised to the total protein amount quantified using a BCA protein assay kit (Thermo Scientific, Cat# 23227) or a Bradford assay kit (Epizyme, Cat# ZJ104). Untreated cells were used as the negative control. Cells treated with RSL3 (0.5

μM) for 24 h were used as the positive control.

20. Extracellular LDH, ATP, and HMGB1 assays

4T1 cells cultured in 6-well plates were treated with RPMI-1640 medium (2.0 mL) containing TAPB (50 μM) and DMTP (75 μM) for 0–24 h. After that, the medium from each well was used for LDH, ATP, and HMGB1 determinations using the LDH assay kit (Dojindo, Cat# CK12), CellTiter-Glo 2.0 assay kit (Promega, Cat# G9242), and mouse HMGB1 ELISA kit (Invitrogen, Cat# EEL102), respectively, according to the manufacturer's guidelines. Cells treated with RSL3 (0.5 μM) for 24 h were used as the positive control of ferroptosis.

21. Extracellular NO, IL6, TNFα, and IL10 assays

4T1 cells cultured in 6-well plates were pretreated with DMSO (0.1 vol%, 2.0 mL) or Fer-1 (1.0 μM, 2.0 mL) for 24 h and treated with RSL3 (0.5 μM, 2.0 mL) or RPMI-1640 medium (2.0 mL) containing TAPB (50 μM) and DMTP (75 μM) for 4 h. The cells were washed twice with PBS and mixed with fresh RPMI-1640 medium. After an additional 20 h of incubation, the medium was mixed with an equal volume of DMEM for incubating RAW264.7 macrophages cultured in 24-well plates. After 24 h of incubation, the medium from each well was used for NO, IL6, TNFα, and IL10 determinations using NO assay kit (Beyotime, Cat# S0021S), mouse IL6 ELISA kit (Proteintech, Cat# KE10007), mouse TNFα ELISA kit (Proteintech, Cat# KE10002), mouse IL10 ELISA kit (Abcam, Cat# ab255729), respectively, according to the manufacturer's guidelines. RAW264.7 macrophages treated with LPS (100 ng/mL, 24 h, Sigma–Aldrich, Cat# L4391) were used as the positive control of classical activation. RAW264.7 macrophages treated with IL4 (10 ng/mL, 12 h, MedChemExpress, Cat# HY-P7080) were used as the positive control of alternative activation. RAW264.7 macrophages treated with the medium of untreated 4T1 cells were used as the negative control group.

22. Western blotting

4T1 cells cultured in 6-well plates were treated with RPMI-1640 medium (2.0 mL) containing TAPB (50 μM) and DMTP (75 μM) in the absence or presence of DMSO (0.1 vol%) and Fer-1 (1.0 μM) for 24 h. The cells were washed once with cold PBS and lysed in RIPA lysis buffer (APExBIO, Cat# K1120) supplemented with protease inhibitor cocktail (MedChemExpress, Cat# HY-K0010) and phosphatase inhibitor cocktail (MedChemExpress, Cat# HY-K0022) at 4°C. The lysates were centrifuged at 13000 rpm for 15 min at 4°C, and the supernatant was isolated to exclude debris. The protein concentration was quantified using a BCA protein assay kit (Thermo Scientific, Cat# 23227). The samples were prepared with SDS–PAGE sample loading buffer (Beyotime, Cat# P0015) at 99°C for 10 min, and equal amounts of protein per sample were loaded onto PAGE gels (Epizyme, Cat# PG112) for electrophoresis to separate the target protein. After transferring the protein to a polyvinylidene difluoride membrane and blocking for 1 h using TBST supplemented with nonfat powdered milk (5 wt%), the membranes were incubated with a primary antibody at 4°C overnight. The primary antibodies used here were rabbit anti-ACSL4 (1:10000 dilution, Abcam, Cat# ab155282), rabbit anti-GPX4 (1:1000 dilution, Abcam, Cat# ab125066), and rabbit anti-β-tubulin (1:1000 dilution, Cell Signaling Technology, Cat# 2128). Subsequently, the membranes were washed three times for 10 min in TBST and incubated with HRP-conjugated goat anti-rabbit IgG (H+L) secondary antibody (1:10000 dilution, Proteintech, Cat# SA00001-2) for 1 h at 25°C. The protein of interest was observed using a Western chemiluminescent HRP substrate (Millipore, Cat# WBKLS0100). A western blot stripping buffer (Cowin Biotech, Cat# CW0056M) was used to dissociate and strip primary and secondary antibodies from Western blots to detect other protein target. GoldBand Plus 3-color regular range protein marker (Yeasen, Cat# 20350ES72) was used for monitoring protein migration.

23. RNA isolation and RT-qPCR

4T1 cells cultured in 6-well plates were treated with RPMI-1640 medium (2.0 mL) containing TAPB (50 μM) and DMTP (75 μM) in the absence or presence of DMSO (0.1 vol%) and Fer-1 (1.0 μM) for 24 h. The cells were carefully rinsed twice with PBS for RNA extraction using RNAfast200 kit (Fastagen, Cat# 220010). The extracted RNA was dissolved in water and the concentration

was determined by measuring absorbance at 260 nm. First-strand complementary DNA was synthesised from 0.5–1.0 μg of total RNA using SuperScript II reverse transcriptase (Invitrogen, Cat# 18064022) following the manufacturer's protocol. PowerUp SYBR Green master mix (Applied Biosystems, Cat# A25742) was used for quantitation according to the manufacturer's protocol. The threshold cycle (*Ct*) values for each gene were normalised to those of *Actb*, and the $2^{-\Delta\Delta Ct}$ method was used for quantitative analysis. Results are represented as fold change from untreated controls.

4T1 cells cultured in 6-well plates were pretreated with DMSO (0.1 vol%, 2.0 mL) or Fer-1 (1.0 μM, 2.0 mL) for 24 h and treated with RSL3 (0.5 μM, 2.0 mL) or RPMI-1640 medium (2.0 mL) containing TAPB (50 μM) and DMTP (75 μM) for 4 h. The cells were washed twice with PBS and mixed with fresh RPMI-1640 medium. After an additional 20 h of incubation, the medium was mixed with an equal volume of DMEM for incubating RAW264.7 macrophages cultured in 6-well plates. After 24 h of incubation, the macrophages were collected for RNA isolation and RT-qPCR according to the above methods.

The following primers were used here:

Actb, forward 5′-CATTGCTGACAGGATGCAGAAGG-3′ and reverse 5′-TGCTGGAAGGTGGACAGTGAGG-3′;

Gpx4, forward 5′-CCTCTGCTGCAAGAGCCTCCC-3′ and reverse 5′-CTTATCCAGGCAGACCATGTGC-3′;

Ptgs2, forward 5′-GCGACATACTCAAGCAGGAGCA-3′ and reverse 5′-AGTGGTAACCGCTCAGGTGTTG-3′;

Acsl4, forward 5′-CCTTTGGCTCATGTGCTGGAAC-3′ and reverse 5′-GCCATAAGTGTGGGTTTCAGTAC-3′;

Il6, forward 5′-TACCACTTCACAAGTCGGAGGC-3′ and reverse 5′-CTGCAAGTGCATCATCGTTGTTC-3′;

Nos2, forward 5′-GAGACAGGGAAGTCTGAAGCAC-3′ and reverse 5′-CCAGCAGTAGTTGCTCCTCTTC-3′.

24. Mitochondrial morphology

For imaging mitochondria of 4T1 cells by confocal laser scanning fluorescence microscopy, 4T1 cells cultured in 4-chamber glass-bottom dishes were treated with RPMI-1640 medium (400 μL) containing TAPB (50 μM) and DMTP (75 μM) in the absence or presence of DMSO (0.1 vol%) and Fer-1 (1.0 μM) for 24 h. The cells were washed twice with PBS, incubated with MitoBright LT Deep Red (100 nM, 200 μL, Dojindo, Cat# MT12) for 30 min in a CO_2 incubator and washed twice with PBS. Laser scanning confocal fluorescence images were captured. The red images of MitoBright LT Deep Red were recorded at an excitation wavelength of 633 nm and an emission wavelength range of 650–700 nm. Untreated cells were used as the negative control group.

25. Mitochondrial membrane potential assays

For assessing mitochondrial membrane potential of 4T1 cells by flow cytometry, 4T1 cells cultured in 12-well plates were treated with RPMI-1640 medium (1.0 mL) containing TAPB (50 μM) and DMTP (75 μM) for 24 h. The cells were harvested using a trypsin–EDTA solution, washed twice with PBS, and incubated with TMRM (50 nM, 1.0 mL, MedChemExpress, Cat# HY-D0984A) for 30 min in a CO_2 incubator. The cells were washed twice with PBS, strained through a 40 μm cell strainer, and analysed using a flow cytometer equipped with a 561 nm laser for excitation and a PE channel for collection.

26. Caspase 3 activation assays

To detect caspase 3 activation of 4T1 cells by a colorimetric assay, 4T1 cells cultured in 6-well plates were treated with RPMI-1640 medium (2.0 mL) containing TAPB (50 μM) and DMTP (75 μM) for 24 h. The cells were carefully rinsed with PBS and used for detecting caspase 3 activity using a caspase 3 colorimetric assay kit (Dojindo, Cat# C551) according to the manufacturer's guidelines. The absorbance at 405 nm was measured every 30 s for a total of 100 min, and the increase rate of the absorbance reflects caspase 3 activity. Untreated cells were used as the negative control group. Cells treated with CPT (2.0 μM) for 24 h were used as the positive control of apoptosis.

27. Annexin V staining for apoptosis detection

For assessing apoptosis of 4T1 cells by flow cytometry, 4T1 cells cultured in 6-well plates were treated with RPMI-1640 medium (2.0 mL) containing TAPB (50 μM) and DMTP (75 μM) in the absence or presence of DMSO (0.1 vol%), Fer-1 (1.0 μM), zVAD (25 μM) for 24 h. The cells were harvested using a trypsin solution, resuspended in PBS, and incubated for 30 min in a CO_2 incubator. Subsequently, the cell apoptosis was analysed using the annexin V apoptosis detection kit (Yeasen, Cat# 40304ES60) according to the kit supplier's guidelines. Finally, the cells were filtered with a 40 μm cell strainer and analysed using a flow cytometer equipped with 488 nm and 638 nm lasers for excitation, and PE and APC channels for collection, respectively. Untreated cells were used as the negative control group. Cells treated with CPT (2.0 μM) for 24 h were used as the positive control of apoptosis.

28. Immunofluorescence staining

For detecting CTSB in 4T1 cells by confocal laser scanning fluorescence microscopy, 4T1 cells cultured in 4-chamber glass-bottom dishes were treated with RPMI-1640 medium (400 μL) containing TAPB (50 μM) or/and DMTP (75 μM) for 24 h. The cells were fixed in methanol (400 μL, precooled at −20°C) for 10 min and washed twice with PBS containing Tween-20 (0.1 vol%). The cells were blocked with PBS (400 μL) containing normal goat serum (10 vol%) and Tween-20 (0.1 vol%) for 1 h at room temperature. The cells were incubated with rabbit anti-CTSB (200 μL, 1:250 dilution, Abcam, Cat# ab214428) for 24 h at 4°C. The cells were washed three times with PBS containing normal goat serum (10 vol%) and Tween-20 (0.1 vol%) and incubated with Alexa Fluor 633-conjugated goat anti-rabbit IgG (H+L) cross-adsorbed secondary antibody (200 μL, 1:500 dilution, Invitrogen, Cat# A-21070) for 2 h at room temperature. The cell nuclei were labelled with PI (20 μM, 200 μL) for 1 h. Finally, the cell was washed twice with PBS for confocal laser scanning fluorescence imaging. The red signals of Alexa Fluor 633 were recorded at an excitation wavelength of 633 nm and an emission wavelength range of 650–700 nm. The signal of PI was not collected to clearly observe the Alexa Fluor 633 fluorescence in the nucleus. High concentrations of PI resulted in elevated contrast of the nucleus in the bright field. The untreated cells were used as the negative control group.

For detecting cleaved caspase 3, PTGS2, HMGB1, and CALR in 4T1 cells by confocal laser scanning fluorescence microscopy, 4T1 cells cultured in 4-chamber glass-bottom dishes were treated with RPMI-1640 medium (400 μL) containing TAPB (50 μM) or/and DMTP (75 μM) in the absence or presence of DMSO (0.1 vol%) and Fer-1 (1.0 μM) for 24 h. The cells were fixed in PFA (4 wt%, 400 μL) for 12 h at 4°C and washed three times with PBS. The cells were permeabilised with Triton X-100 (0.5 vol%, 400 μL) for 5 min and washed twice with PBS. The cells were then blocked with PBS (400 μL) containing normal goat serum (10 vol%) and Tween-20 (0.1 vol%) for 1 h at room temperature. The cells were incubated with primary antibodies (100 μL) for 24 h at 4°C. The primary antibodies used here were rabbit anti-cleaved caspase 3 (1:2000 dilution, Cell Signaling Technology, Cat# 9579T), rabbit anti-PTGS2 (1:400 dilution, Cell Signaling Technology, Cat# 12282S), rabbit anti-HMGB1 (1:250 dilution, Abcam, Cat# ab79823), and rabbit anti-CALR (1:400 dilution, Cell Signaling Technology, Cat# 12238S). The cells were washed three times with PBS containing normal goat serum (10 vol%) and Tween-20 (0.1 vol%) and incubated with Alexa Fluor 594-conjugated goat anti-rabbit IgG (H+L) secondary antibody (200 μL, 1:500 dilution, Abcam, Cat# ab150080) for 1 h at room temperature. Finally, the cell nuclei were counterstained with Nuclear Red LCS1 (2.0 μM, 200 μL, AAT Bioquest, Cat# 17542) for 10 min and washed twice with PBS. Confocal laser scanning fluorescence images were captured. The cyan signals of Nuclear Red LCS1 were recorded at an excitation wavelength of 633 nm and an emission wavelength range of 650–700 nm. The red signals of Alexa Fluor 594 were recorded at an excitation wavelength of 561 nm and an emission wavelength range of 580–620 nm. The untreated well was used as the negative control group. Cells treated with CPT (2.0 μM) for 24 h were used as the positive control of apoptosis. Cells treated with RSL3 (0.5 μM) for 24 h were used as the positive control of ferroptosis.

For detecting membrane-expressed CALR of 4T1 cells by flow cytometry, 4T1 cells cultured in 12-well plates were treated with RPMI-1640 medium (1.0 mL) containing TAPB (50 μM) and DMTP (75 μM) for 24 h. The cells were harvested with a trypsin–EDTA solution and washed twice with PBS. The cells were then blocked with PBS (400 μL) containing normal goat serum (10 vol%) and Tween-20 (0.1 vol%) for 1 h at room temperature. The cells were incubated with rabbit anti-CALR (100 μL, 1:200

dilution, Cell Signaling Technology, Cat# 12238S) for 1 h at 4°C. The cells were washed three times with PBS containing normal goat serum (10 vol%) and Tween-20 (0.1 vol%) and incubated with Alexa Fluor 647-conjugated goat anti-rabbit IgG (H+L) secondary antibody (100 μL, 1:2000 dilution, Abcam, Cat# ab150079) for 1 h at room temperature. The cells were mixed with 7-AAD (5.0 μM, 500 μL) and analysed using a flow cytometer equipped with 488 nm and 638 nm lasers for excitation, and PC5.5 and APC channels for collection, respectively. Untreated cells were used as the negative control group. Cells treated with RSL3 (0.5 μM) for 24 h were used as the positive control.

For detecting CD80 and CD86 expressions of RAW264.7 macrophages by flow cytometry, 4T1 cells cultured in 6-well plates were pretreated with DMSO (0.1 vol%, 2.0 mL) or Fer-1 (1.0 μM, 2.0 mL) for 24 h. The cells were washed twice with PBS and then treated with RSL3 (0.5 μM, 2.0 mL) or RPMI-1640 medium (2.0 mL) containing TAPB (50 μM) and DMTP (75 μM) for 4 h. The cells were washed twice with PBS and mixed with fresh RPMI-1640 medium. After an additional 20 h of incubation, the medium was mixed with an equal volume of DMEM and used for incubating RAW264.7 macrophages cultured in 6-well plates. After 24 h of incubation, the macrophages were collected by centrifugation at 1500 rpm for 10 min at 4°C and washed once with PBS. The macrophages were incubated with primary antibodies (100 μL) for 2 h at 4°C. The primary antibodies used here were FITC-conjugated anti-mouse CD80 (1:100 dilution, BioLegend, Cat# 104706), and PerCP-Cy5.5-conjugated anti-mouse CD86 (1:100 dilution, BioLegend, Cat# 105028). The macrophages were washed with PBS and analysed using a flow cytometer equipped with a 488 nm laser for excitation. RAW264.7 macrophages treated with LPS (100 ng/mL, 24 h, Sigma–Aldrich, Cat# L4391) were used as the positive control of classical activation. RAW264.7 macrophages treated with the medium of untreated 4T1 cells were used as the negative control group.

For detecting CD206 expression of RAW264.7 macrophages by flow cytometry, 4T1 cells cultured in 6-well plates were treated with RPMI-1640 medium (2.0 mL) containing TAPB (50 μM) and DMTP (75 μM) for 4 h. The cells were washed twice with PBS and mixed with fresh RPMI-1640 medium. After an additional 20 h of incubation, the medium was mixed with an equal volume of DMEM and used for incubating RAW264.7 macrophages cultured in 6-well plates. After 24 h of incubation, the macrophages were collected by centrifugation at 1500 rpm for 10 min at 4°C and washed once with PBS. The macrophages were fixed in an intracellular fixation buffer (200 μL, 1:1 dilution, Invitrogen, Cat# 00-8222-49) for 1 h at 4°C. The macrophages were washed and permeabilized using a permeabilization buffer (200 μL, 1:9 dilution, Invitrogen, Cat# 00-8333-56) for 1 h at 4°C. The macrophages were incubated with FITC-conjugated anti-mouse CD206 (1:800 dilution, BioLegend, Cat# 141703) diluted in a permeabilization buffer (200 μL, 1:9 dilution, Invitrogen, Cat# 00-8333-56) for 2 h at 4°C, and washed twice carefully. The macrophages were analyzed using a flow cytometer equipped with a 488 nm laser for excitation and an FITC channel for collection. RAW264.7 macrophages treated with IL4 (10 ng/mL, 12 h, MedChemExpress, Cat# HY-P7080) were used as the positive control of alternative activation. RAW264.7 macrophages treated with the medium of untreated 4T1 cells were used as the negative control group.

29. Phagocytosis assays

4T1 cells cultured in 6-well plates were treated with RSL3 (0.5 μM, 2.0 mL) or RPMI-1640 medium (2.0 mL) containing TAPB (50 μM) and DMTP (75 μM) for 4 h in a CO_2 incubator. The cells were washed twice with PBS and resuspended in fresh RPMI-1640 medium. After an additional 20 h of incubation, the medium was mixed with an equal volume of DMEM for incubation of RAW264.7 macrophages cultured in 12-well plates. After 24 h of incubation, the macrophages were collected by centrifugation at 1500 rpm for 10 min at 4°C and washed once with PBS for further experiments.

To evaluate the interaction between macrophages and 4T1 cells, the collected macrophages and 4T1 cells were covalently labeled with CellTrace CFSE (5.0 μM, 1.0 mL, Invitrogen, Cat# C34554) and CellTracker Red CMTPX (2.0 μM, 1.0 mL, Invitrogen, Cat# C34552), respectively, for 30 min in a CO_2 incubator and washed three times with DMEM. Subsequently, the green fluorescently labeled macrophages (2×10^4 or 4×10^4 cells) were mixed with the red fluorescently labeled 4T1 cells (5×10^3 cells) in DMEM (200 μL). After 24 h of incubation in a CO_2 incubator, red and green fluorescence images were collected.

To quantify the phagocytic capacity of macrophages on polystyrene microspheres, the collected macrophages were incubated with coumarin 6-labeled polystyrene microspheres (50 µg/mL, 500 µL, 2 µm in diameter) in a CO_2 incubator for 3 h. The macrophages were washed twice with cold PBS and analysed using a flow cytometer with a 488 nm laser for excitation and an FITC channel for detection.

30. Haemolytic tests

TAPB (0–0.8 mM, 500 µL) or DMTP (0–1.2 mM, 500 µL) was mixed with red blood cells (500 µL, approximately 3×10^5 cells/µL), followed by incubation at 37°C for 1 h. Subsequently, the red blood cells were separated by centrifugation at 12000 rpm for 90 s. The absorbance at 545 nm of the supernatants was measured using a multimode microplate detection system. Triton X-100 (0.5 vol%) was used as a positive control with a haemolytic rate defined as 100%, and PBS was used as a negative control with a haemolytic rate defined as 0%.

31. Antitumour treatment for a unilateral 4T1 tumour model of BALB/c mice

4T1 cells (approximately 5×10^6 cells) suspended in HBSS (50 µL) were subcutaneously injected into the flank of each BALB/c mouse (aged 4–5 weeks, female) to establish the unilateral 4T1 tumour model.

When the tumour size reached approximately 100 mm^3, the mice were randomly divided into 5 groups. Groups i, ii, iii, and iv were intratumourally injected with Tween-80 (0.05 vol%) in normal saline (50 µL), TAPB (0.4 mM, 50 µL), DMTP (0.6 mM, 50 µL), and a mixture (50 µL) of TAPB (0.4 mM) and DMTP (0.6 mM) on Day 0, respectively. Group v was peritumourally injected with Lip-1 (5.0 mM, 50 µL) on Days 1, 4, 7, and 10, in addition to the same treatment as group iv. Here, TAPB and DMTP were dissolved in normal saline containing Tween-80 (0.05 vol%), and Lip-1 were dissolved in normal saline containing PEG300 (50 vol%).

After 24 h of TAPB and DMTP co-injection, some mice were euthanised. Their tumour tissues were collected and rapidly sliced into thin sections in glutaraldehyde (2.5 vol%, 10 mL). The tumour sections were stored in glutaraldehyde (2.5 vol%, 1.5 mL) at 4°C. On the analysis day, the tumour sections were dehydrated in a graded ethanol series, embedded in an epoxy resin, and cut into 50–80 nm thick sections for bio-TEM examination.

The remaining mice were kept until a tumour size of 15 mm was reached in either dimension. At the treatment endpoint, all mice were euthanised, and their tumour tissues and the major organs were harvested and washed with PBS for further experiments.

A portion of the tumour tissues were homogenised and used for GSH, MDA, PGE2, IL6, TNFα, and IFNγ measurements using the GSH assay kit (Nanjing Jiancheng Bioengineering Institute, Cat# A006-2-1), MDA assay kit (Nanjing Jiancheng Bioengineering Institute, Cat# A003-2-2), PGE2 ELISA kit (Cayman, Cat# 514010), mouse IL6 ELISA kit (Proteintech, Cat# KE10007), mouse TNFα ELISA kit (Proteintech, Cat# KE10002), and mouse IFNγ ELISA kit (Proteintech, Cat# KE10094), respectively, according to the manufacturer's guidelines.

A portion of the tumour tissues and major organs were fixed in PFA (4 wt%), dehydrated, embedded in paraffin, and sectioned at a thickness of 3–4 µm according to standard protocols. The slices were stored at room temperature until used for H&E and IHC staining.

A portion of the spleens were used for immunophenotyping by flow cytometry.

32. Antitumour treatment for a unilateral B16F10 tumour model of C57BL/6N mice

C57BL/6N mice (6–7 weeks of age, female) were purchased from Vital River (Beijing, China) and housed in a filter-topped pathogen-free facility. The mice were provided with ^{60}Co-irradiated food and autoclaved water and kept in a room with a 12:12 h light–dark cycle, a temperature between 20–23°C, and 30–70% relative humidity.

B16F10 cells (approximately 10^6 cells) suspended in HBSS (50 µL) were subcutaneously injected into the flank of each C57BL/6N mouse to establish the unilateral B16F10 tumour model. When the tumour size reached 50 mm^3, the mice were randomly divided into 6 groups. Groups G1, G2, and G3 were intratumourally injected with normal saline (50 µL), TAPB (0.4 mM, 50 µL), and DMTP (0.6 mM, 50 µL) on Day 0, respectively. Group G4 was peritumourally injected with Lip-1 (5.0 mM, 50 µL) on Days 1, 4, 7, and 10. Group G5 was intratumourally injected with a mixture (50 µL) of TAPB (0.4 mM) and DMTP (0.6 mM) on Day 0. Group G6 was intratumourally injected with a mixture (50 µL) of TAPB (0.4 mM) and DMTP (0.6 mM) on Day 0 and peritumourally injected with Lip-1 (5.0 mM, 50 µL) on Days 1, 4, 7, and 10. Here, TAPB and DMTP were dissolved in normal saline containing Tween-80 (0.05 vol%), and Lip-1 were dissolved in normal saline containing PEG300 (50 vol%).

The mice were kept until a tumour size of 20 mm was reached in either dimension. At the treatment endpoint, serum samples were obtained from the submandibular vein and analysed for IL6, TNFα, and IFNγ levels using the mouse IL6 ELISA kit (Proteintech, Cat# KE10007), mouse TNFα ELISA kit (Proteintech, Cat# KE10002), and mouse IFNγ ELISA kit (Proteintech, Cat# KE10094), respectively, following the manufacturer's guidelines. After blood collection, all mice were euthanised, and their tumour tissues and the major organs were harvested and washed with PBS for further experiments.

A portion of the tumour tissues were homogenised and used for GSH, MDA, and PGE2 measurements using the GSH assay kit (Nanjing Jiancheng Bioengineering Institute, Cat# A006-2-1), MDA assay kit (Nanjing Jiancheng Bioengineering Institute, Cat# A003-2-2), and PGE2 ELISA kit (Cayman, Cat# 514010), respectively, according to the manufacturer's guidelines.

A portion of the tumour tissues and major organs were fixed in PFA (4 wt%), dehydrated, embedded in paraffin, and sectioned at a thickness of 3–4 µm according to standard protocols. The slices were stored at room temperature until used for H&E and IHC staining.

A portion of the spleens were used for immunophenotyping by flow cytometry.

33. Antitumour treatment for a bilateral 4T1 tumour model of BALB/c mice

For establishing a bilateral 4T1 tumour model of BALB/c mice (aged 4–5 weeks, female), 4T1 cells (approximately 5×10^6 cells) suspended in HBSS (50 µL) were subcutaneously injected into the right side of each mouse; two days later, the same number of cells were inoculated subcutaneously on the left side of the mice. When the right tumour size reached approximately 100 mm^3, the mice were randomly divided into 4 groups and the right tumour was treated on Days 0, 3, and 6. Group vi received an intratumoural injection of normal saline (50 µL) containing Tween-80 (0.025 vol%) and a peritumoural injection of normal saline (50 µL) containing SBE-β-CD (20 wt%). Group vii received an intratumoural injection of normal saline (50 µL) containing Tween-80 (0.025 vol%) and a peritumoural injection of R848 (2.5 mM, 50 µL). Group viii received an intratumoural injection of a mixture (50 µL) containing TAPB (0.2 mM) and DMTP (0.3 mM), as well as a peritumoural injection of normal saline (50 µL) containing SBE-β-CD (20 wt%). Group ix received an intratumoural injection of a mixture (50 µL) containing TAPB (0.2 mM) and DMTP (0.3 mM), as well as a peritumoural injection of R848 (2.5 mM, 50 µL) on Days 0, 3, and 6. Here, TAPB and DMTP were dissolved in normal saline containing Tween-80 (0.025 vol%), and R848 was dissolved in normal saline containing SBE-β-CD (20 wt%).

The mice were kept until a tumour size of 15 mm was reached in either dimension. At the treatment endpoint, plasma samples were obtained from the submandibular vein and analysed for IL6, IL10, TNFα, and IFNγ levels using the mouse IL6 ELISA kit (Proteintech, Cat# KE10007), mouse IL10 ELISA kit (Abcam, Cat# ab255729), mouse TNFα ELISA kit (Proteintech, Cat# KE10002), mouse IFNγ ELISA kit (Proteintech, Cat# KE10094), respectively, following the manufacturer's guidelines. After that, all mice were euthanised. Tumour tissues and major organs were harvested and washed with PBS. A portion of spleens and abscopal tumours were used for immunophenotyping by flow cytometry. The remaining tumour tissues and organs were fixed in PFA (4 wt%), dehydrated, embedded in paraffin, and sectioned at a thickness of 3–4 µm according to standard protocols. The slices were stored at room temperature until used for H&E and IHC staining.

34. Tumour vaccination for BALB/c mice and NU/NU nude mice

4T1 cells cultured in T225 flasks were treated with RPMI-1640 medium (30 mL) containing TAPB (50 μM) and DMTP (75 μM) for 24 h. The cells were washed once with PBS and harvested using a trypsin–EDTA solution. The cells (approximately 2×10^8 cells) were resuspended in PBS (0.7 mL) and then snap-frozen in liquid nitrogen to prepare whole-cell lysates. After returning to room temperature, the lysates were mixed with VitroGel hydrogel matrix (1.4 mL, TheWell, Cat# VHM01) and incubated for 10 min at room temperature to obtain the vaccine hydrogel. Control hydrogel was prepared from the same concentration of untreated 4T1 cells using similar methods.

Group x (NU/NU nude mice, n = 10) and group xii (BALB/c mice, n = 10) received a subcutaneous injection of the control hydrogel (50 μL) on their left sides once a week for a total of three injections. Group xi (NU/NU nude mice, n = 10) and group xiii (BALB/c mice, n = 10) received a subcutaneous injection of the vaccine hydrogel (50 μL) on their left sides once a week for a total of three injections. One week after the third injection, 4T1 cells (approximately 10^6 cells) suspended in HBSS (50 μL) were subcutaneously injected into the right side of each mouse. The mice were kept until a tumour size of 15 mm was reached in either dimension.

35. Immunophenotyping by flow cytometry

To prepare single cell suspensions of mouse spleens, the collected spleens were cut into small pieces in HBSS (1.0 mL) and then mixed with HBSS (1.0 mL) containing collagenase IV (2.0 mg/mL, Yeasen, Cat# 40510ES60) and DNase I (40 U/mL, Yeasen, Cat# 10607ES15), followed by incubation for 1 h at 37°C. The dissociated tissues were crushed with a syringe plunger, strained through a 40 μm cell strainer, and rinsed with HBSS. The cells were collected by centrifugation at 1000 rpm for 6 min at 4°C and resuspended in a RBC lysis buffer (4.0 mL, Invitrogen, Cat# 00-4333-57), followed by incubation for 10 min at 4°C. Finally, the cells were washed twice with cold PBS and resuspended in a flow cytometry staining buffer (Invitrogen, Cat# 00-4222-26) at a concentration of 2×10^7 cells/mL.

To prepare single cell suspensions of mouse tumours, the collected tumours were cut into small pieces in HBSS (1.0 mL) and then mixed with HBSS (1.0 mL) containing collagenase II (2.0 mg/mL, Yeasen, Cat# 40508ES60), hyaluronidase (2.0 mg/mL, Yeasen, Cat# 20426ES60), and DNase I (40 U/mL, Yeasen, Cat# 10607ES15), followed by incubation for 2.5 h at 37°C. The dissociated tissues were crushed with a syringe plunger, strained through a 70 μm cell strainer, and rinsed with HBSS. The cells were collected by centrifugation at 1000 rpm for 6 min at 4°C and resuspended in a RBC lysis buffer (4.0 mL, Invitrogen, Cat# 00-4333-57), followed by incubation for 10 min at 4°C. Finally, the cells were washed twice with cold PBS and resuspended in a flow cytometry staining buffer (Invitrogen, Cat# 00-4222-26) at a concentration of 2×10^7 cells/mL.

For flow cytometry staining of the cells obtained from BALB/c mice and NU/NU nude mice, the cells were blocked with anti-CD16/CD32 (100 μL, 1:200 dilution, Invitrogen, Cat# 16-0161-85) for 1 h at 4°C. The cells were incubated with primary antibodies diluted in flow cytometry staining buffer (100 μL, Invitrogen, Cat# 00-4222-26) for 2 h at 4°C. The primary antibodies used here were PE-Cy7-conjugated anti-CD45 (1:80 dilution, BioLegend, Cat# 103114), FITC-conjugated anti-CD11b (1:100 dilution, Invitrogen, Cat# 11-0112-85), PerCP-Cy5.5-conjugated anti-F4/80 (1:40 dilution, Invitrogen, Cat# 45-4801-82), PE-conjugated anti-CD86 (1:160 dilution, Invitrogen, Cat# 12-0862-82), Alexa Fluor 700-conjugated anti-CD11c (1:80 dilution, Invitrogen, Cat# 56-0114-82), APC-conjugated anti-CD80 (1:333 dilution, Invitrogen, Cat# 17-0801-81), FITC-conjugated anti-CD3 (1:200 dilution, Invitrogen, Cat# 11-0032-82), eFluor 450-conjugated anti-CD4 (1:80 dilution, Invitrogen, Cat# 48-0041-82), PerCP-Cy5.5-conjugated anti-CD8a (1:80 dilution, Invitrogen, Cat# 45-0081-82), PE-conjugated anti-CD44 (1:160 dilution, Invitrogen, Cat# 12-0441-81), and APC-conjugated anti-CD62L (1:333 dilution, Invitrogen, Cat# 17-0621-81). The cells were then washed with flow cytometry staining buffer (1.0 mL, Invitrogen, Cat# 00-4222-26) and stained using the live/dead fixable aqua dead cell stain kit (Invitrogen, Cat# L34966) following the manufacturer's instructions. The cells were fixed in an intracellular fixation buffer (200 μL, 1:1 dilution, Invitrogen, Cat# 00-8222-49) for 1 h at 4°C. The cells were washed and permeabilized using a permeabilization buffer (200 μL, 1:9 dilution, Invitrogen, Cat# 00-8333-56) for 1 h at 4°C. The cells

were incubated with APC-conjugated anti-CD206 (1:80 dilution, Invitrogen, Cat# 17-2061-82) diluted in a permeabilization buffer (100 µL, 1:9 dilution, Invitrogen, Cat# 00-8333-56) for 2 h at 4°C. Finally, the cells were washed with flow cytometry staining buffer (1.0 mL, Invitrogen, Cat# 00-4222-26) and analysed using a flow cytometer equipped with 405, 488, 561, and 640 nm lasers. The dead cells labeled with the live/dead fixable aqua dead cell stain kit were detected in the BV510 channel and were excluded from the analysis. For each tube of sample, 3×10^5 – 1×10^6 events were collected.

For flow cytometry staining of the cells obtained from C57BL/6N mice, the cells were blocked with anti-CD16/CD32 (100 µL, 1:200 dilution, Invitrogen, Cat# 16-0161-85) for 2 h at 4°C. The cells were incubated with primary antibodies diluted in flow cytometry staining buffer (100 µL, Invitrogen, Cat# 00-4222-26) for 2 h at 4°C. The primary antibodies used here were PE-Cy7-conjugated anti-CD45 (1:80 dilution, BioLegend, Cat# 103114), FITC-conjugated anti-CD11b (1:100 dilution, Invitrogen, Cat# 11-0112-85), PerCP-Cy5.5-conjugated anti-F4/80 (1:40 dilution, Invitrogen, Cat# 45-4801-82), PE-conjugated anti-CD86 (1:160 dilution, Invitrogen, Cat# 12-0862-82), Alexa Fluor 700-conjugated anti-CD11c (1:80 dilution, Invitrogen, Cat# 56-0114-82), APC-conjugated anti-CD80 (1:333 dilution, Invitrogen, Cat# 17-0801-81), FITC-conjugated anti-CD3 (1:200 dilution, Invitrogen, Cat# 11-0032-82), eFluor 450-conjugated anti-CD4 (1:80 dilution, Invitrogen, Cat# 48-0041-82), and PerCP-Cy5.5-conjugated anti-CD8a (1:80 dilution, Invitrogen, Cat# 45-0081-82). The cells were stained using the live/dead fixable aqua dead cell stain kit (Invitrogen, Cat# L34966) following the manufacturer's instructions, and washed with flow cytometry staining buffer (1.0 mL, Invitrogen, Cat# 00-4222-26). The cells were resuspended in flow cytometry staining buffer (100 µL, Invitrogen, Cat# 00-4222-26), mixed with intracellular fixation buffer (100 µL, Invitrogen, Cat# 00-8222-49), and stored in the dark at 4°C for 1 h. The cells were washed and permeabilized using a permeabilization buffer (200 µL, 1:9 dilution, Invitrogen, Cat# 00-8333-56) for 1 h at 4°C. The cells were incubated with APC-conjugated anti-CD206 (1:80 dilution, Invitrogen, Cat# 17-2061-82) diluted in a permeabilization buffer (100 µL, 1:9 dilution, Invitrogen, Cat# 00-8333-56) for 2 h at 4°C. Finally, the cells were washed with flow cytometry staining buffer (1.0 mL, Invitrogen, Cat# 00-4222-26) and stored in the dark at 4°C until analysis by a flow cytometer equipped with 405, 488, 561, and 640 nm lasers. The dead cells labelled with the live/dead fixable aqua dead cell stain kit were detected in the BV510 channel and were excluded from the analysis. For each tube of sample, at least 3×10^5 events were collected.

36. H&E and IHC staining

For H&E staining, the slices were deparaffinised, rehydrated, and subsequently stained with an H&E stain kit (Nanjing Jiancheng Bioengineering Institute, Cat# D006-1-1) according to the manufacturer's guidelines.

For IHC staining of Ki67, CD4, and CD8, antigen retrieval of the deparaffinised and rehydrated slices was achieved by microwaving in citrate antigen retrieval buffer (pH 6.0, ZSGB-BIO, Cat# ZLI-9064). For IHC staining of GPX4 and PTGS2, antigen retrieval of the deparaffinised and rehydrated slices was achieved by microwaving in Tris–EDTA antigen retrieval buffer (pH 9.0, ZSGB-BIO, Cat# ZLI-9079). Then, the slices were permeabilized with Triton X-100 (0.3 vol%) for 15 min, treated with H_2O_2 (0.3 wt%) for 10 min, and incubated with normal goat serum blocking solution (ZSGB-BIO, Cat# ZLI-9022) for 1 h. The slices were incubated with primary antibody overnight at 4°C. The bound antibodies were detected using a polymer-enhanced two-step immunohistochemistry detection system (ZSGB-BIO, Cat# PV-9001) and a diaminobenzidine kit (ZSGB-BIO, Cat# ZLI-9018) following the manufacturer's protocols. The antibodies used here were rabbit anti-Ki67 (1:200 dilution, Abcam, Cat# ab16667), rabbit anti-CD4 (1:400 dilution, Servicebio, Cat# GB15064-100), rabbit anti-CD8a (1:400 dilution, Servicebio, Cat# GB15068-100), rabbit anti-GPX4 (1:200 dilution, Abcam, Cat# ab125066), and rabbit anti-PTGS2 (1:400 dilution, Cell Signaling Technology, Cat# 12282S). Nuclei were counterstained with hematoxylin (Nanjing Jiancheng Bioengineering Institute, Cat# D006-1-1).

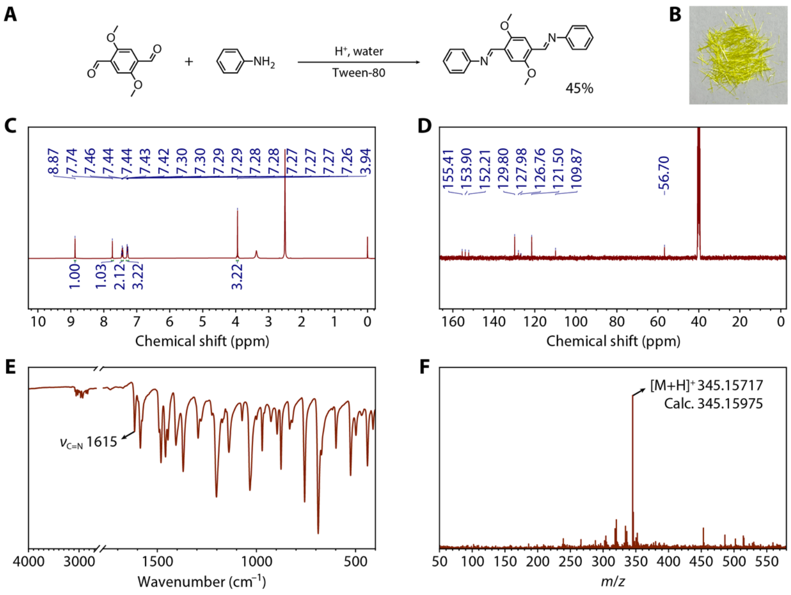

Figure S1. Synthesis and characterisation of (1*E*,1'*E*)-1,1'-(2,5-dimethoxy-1,4-phenylene)bis(*N*-phenylmethanimine).

(A) Synthetic reaction.

(B) Photograph of the single crystal.

(C) ^{1}H NMR spectrum.

(D) ^{13}C NMR spectrum.

(E) ATR–FTIR spectrum.

(F) HRMS.

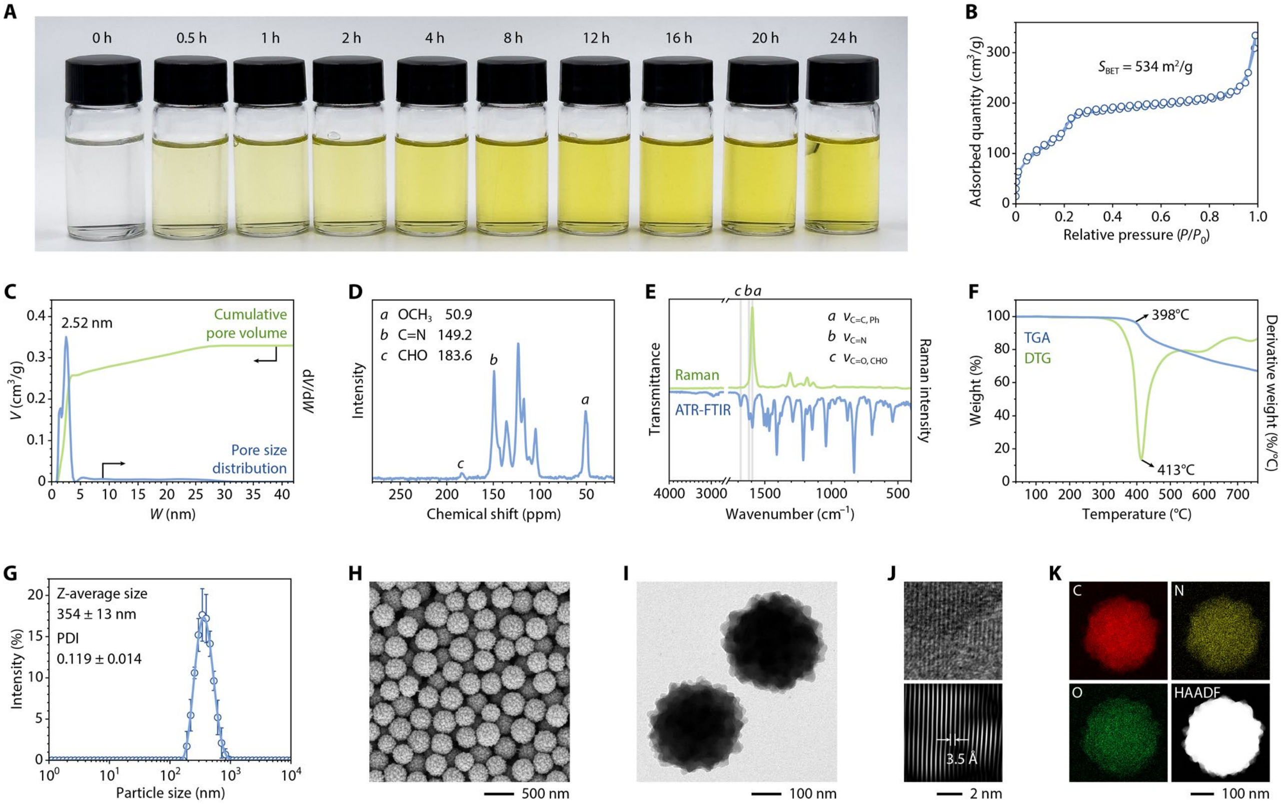

Figure S2. Characterisation of UMCOF1.

(A) Digital photographs of the reaction bottle charged with a phosphate buffer (10 mM, pH 5.0) containing TAPB (50 µM), DMTP (75 µM), and Tween-80 (0.0213 vol%) at 37°C for 0–24 h.

(B) N_2 adsorption–desorption isotherms of **UMCOF1** powder at 77 K.

(C) Pore size distribution and cumulative pore volume plots of **UMCOF1** powder.

(D) ^{13}C CP–MAS NMR spectrum of **UMCOF1** powder.

(E) ATR–FTIR and Raman spectra of **UMCOF1** powder.

(F) Thermogravimetric and corresponding differential thermogravimetric curves of the **UMCOF1** powder in N_2.

(G) Particle size distribution plot of **UMCOF1** measured by dynamic light scattering in PBS. Data are expressed as mean ± SD; n = 3 independent experiments.

(H) SEM image of **UMCOF1** nanoparticles.

(I) TEM image of **UMCOF1** nanoparticles.

(J) Lattice-resolution TEM image of **UMCOF1** nanoparticles and its bandpass-filtered images with enhanced contrast.

(K) Elemental mapping of the **UMCOF1** nanoparticles.

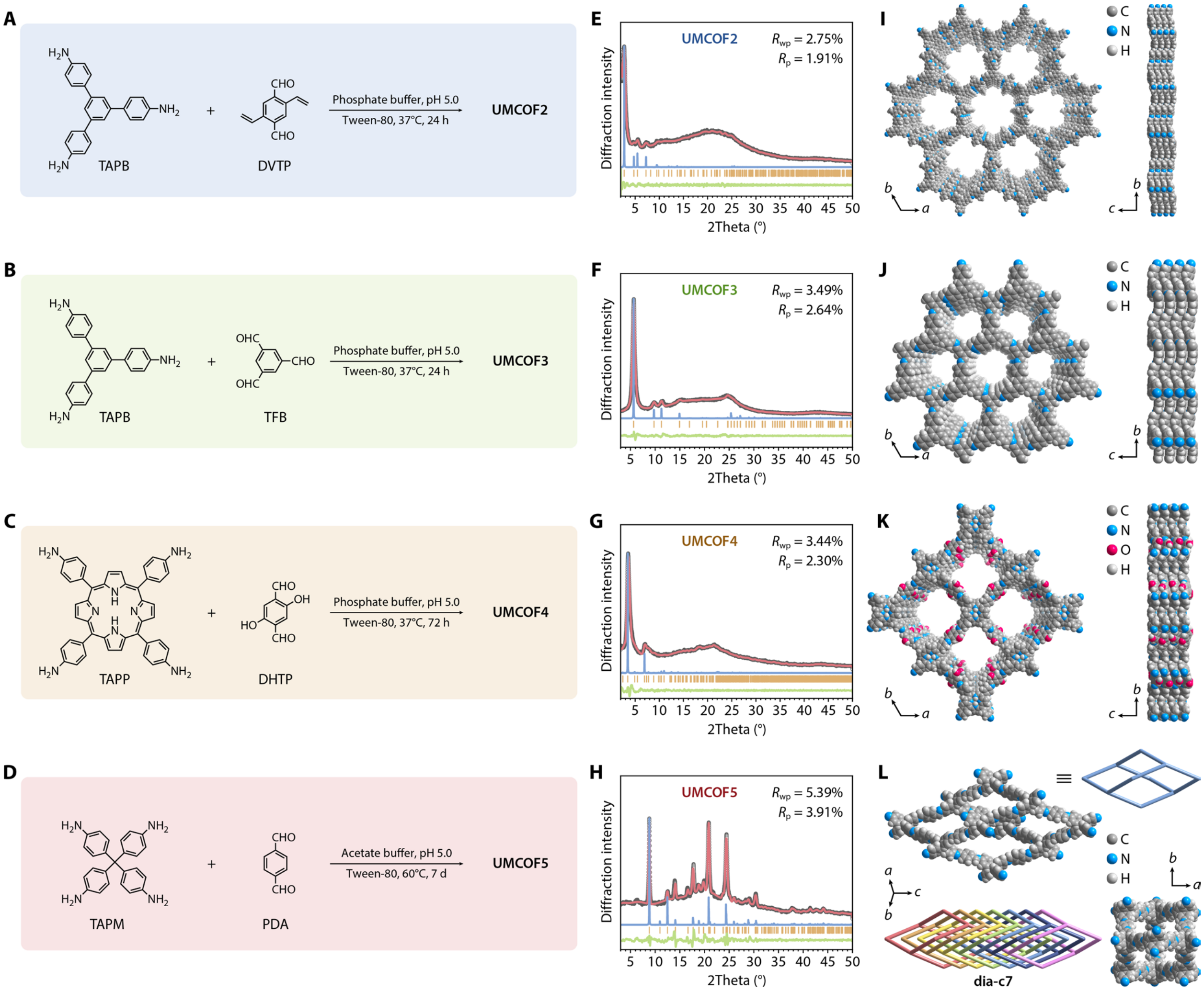

Figure S3. Synthesis and structure of UMCOF2–5.

(A–D) Synthetic schemes for **UMCOF2** (A), **UMCOF3** (B), **UMCOF4** (C), and **UMCOF5** (D).

(E–H) Experimental (grey dots), Pawley-refined (red), and simulated (blue) PXRD patterns; difference plot (green); and the Bragg positions (brown) of **UMCOF2** (E), **UMCOF3** (F), **UMCOF4** (G), and **UMCOF5** (H).

(I–L) Proposed framework structures of **UMCOF2** (I), **UMCOF3** (J), **UMCOF4** (K), and **UMCOF5** (L).

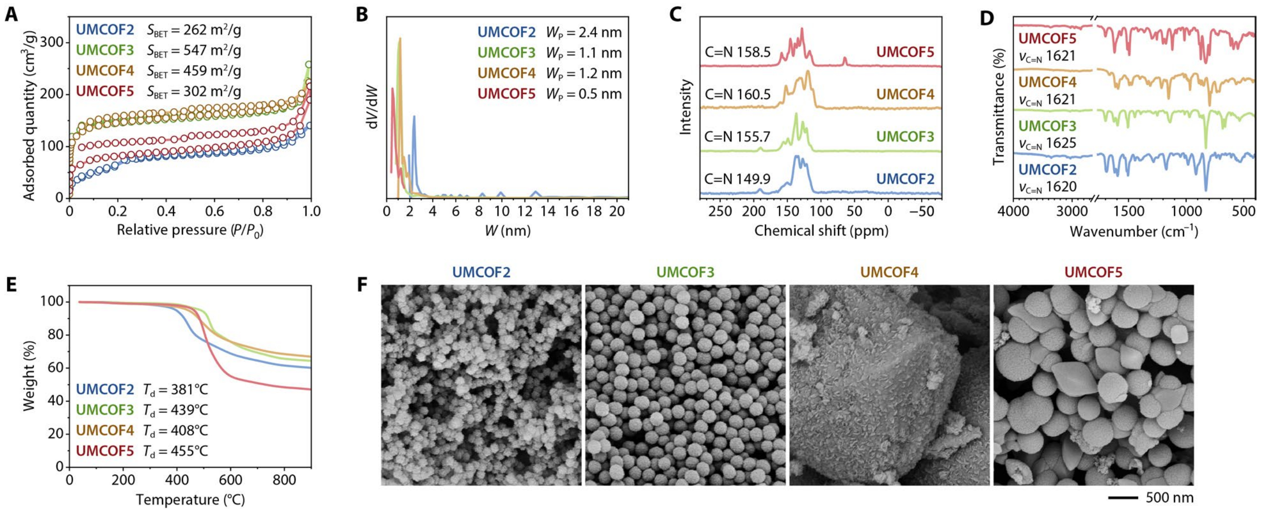

Figure S4. Characterisation of the UMCOF2–5 powders.

(A) N_2 adsorption–desorption isotherms at 77 K.

(B) Pore size distribution plots.

(C) ^{13}C CP–MAS NMR spectra.

(D) ATR–FTIR spectra.

(E) TGA curves measured under N_2 flow.

(F) SEM images.

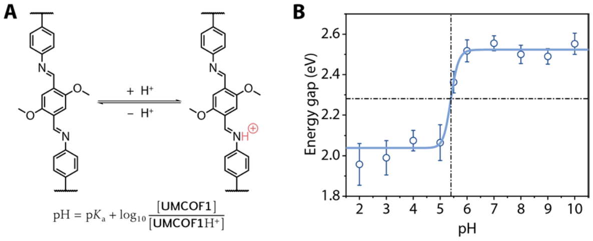

Figure S5. pK_a of the protonated UMCOF1.

(A) Imine protonation of the **UMCOF1** linkages.

(B) Calculation of the apparent pK_a of the protonated **UMCOF1** using the Henderson–Hasselbalch equation, where [**UMCOF1**]/[**UMCOF1**H$^+$] is calculated by determining the energy band gap of the **UMCOF1** powder soaked in buffer solutions with different pH values for 30 min. Data are expressed as mean ± SD; n = 4 independent experiments.

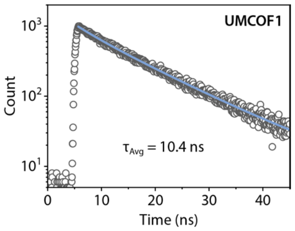

Figure S6. Time-resolved photoluminescence spectra of UMCOF1 with an excitation wavelength of 405 nm and an emission wavelength of 528 nm.

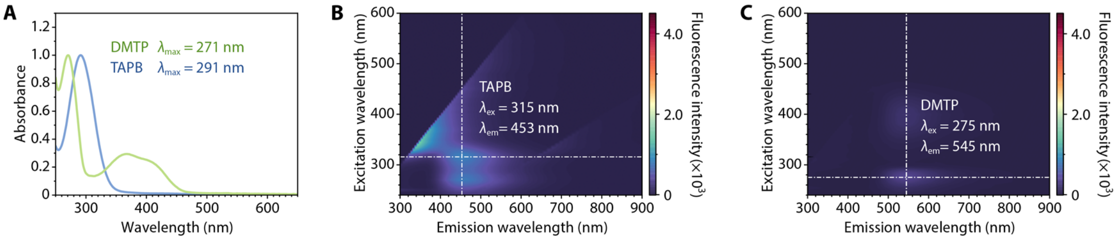

Figure S7. Spectroscopic properties of TAPB and DMTP dissolved in Tween-80 (0.0213 vol%).

(A) UV–vis spectra of TAPB and DMTP.

(B) Excitation–emission matrix of TAPB (50 μM).

(C) Excitation–emission matrix of DMTP (75 μM).

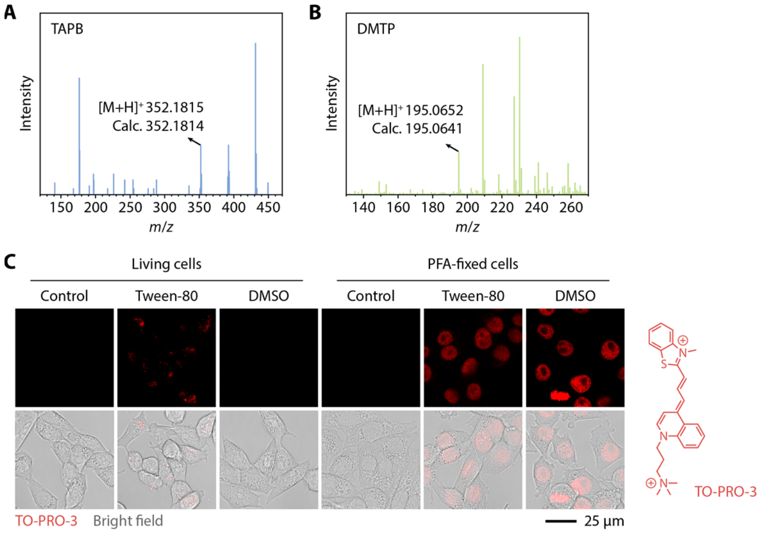

Figure S8. Cellular uptake of organic monomers.

(A) HRMS (ESI source) of the lysed 4T1 cells treated with TAPB (50 μM) for 24 h.

(B) HRMS (EI source) of the lysed 4T1 cells treated with DMTP (75 μM) for 24 h.

(C) Confocal laser scanning fluorescence images of TO-PRO-3 (1.0 μM, 30 min) staining for evaluating membrane permeability toward living 4T1 cells and PFA-fixed 4T1 cells in the absence or presence of Tween-80 (0.02 vol%).

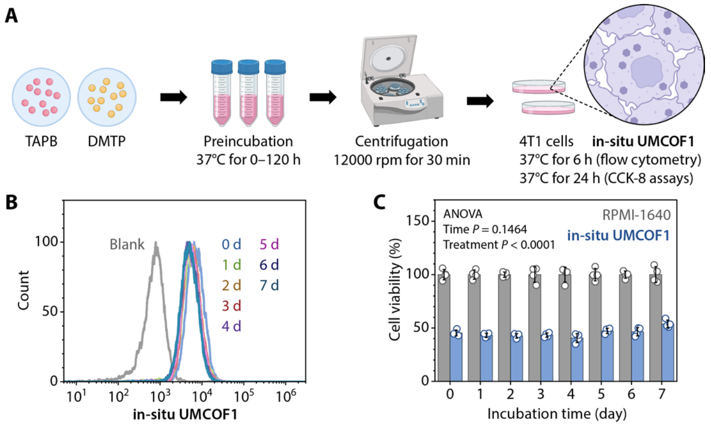

Figure S9. Premixed organic monomers in RPMI-1640 medium for intracellular in-situ UMCOF1 synthesis.

(A) Schematic of the experimental design. Schematic created using BioRender.com.

(B) Flow cytometric analysis of 4T1 cells treated with premixed TAPB (50 μM) and DMTP (75 μM) in RPMI-1640 medium for 6 h.

(C) CCK-8 cell viability assays of 4T1 cells treated with premixed TAPB (50 μM) and DMTP (75 μM) in RPMI-1640 medium for 24 h. Data are expressed as mean ± SD (n = 4 technical replicates) and compared by two-way ANOVA.

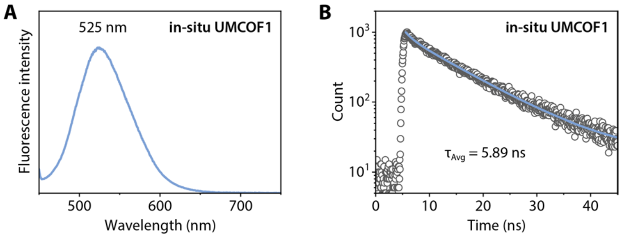

Figure S10. Spectroscopic properties of in-situ UMCOF1 isolated from the 4T1 cells treated with TAPB (50 μM) and DMTP (75 μM) for 24 h.

(A) Emission spectrum of **in-situ UMCOF1** with an excitation wavelength of 405 nm.

(B) Time-resolved photoluminescence spectra of **in-situ UMCOF1** with an excitation wavelength of 405 nm as well as emission wavelengths of 525 nm.

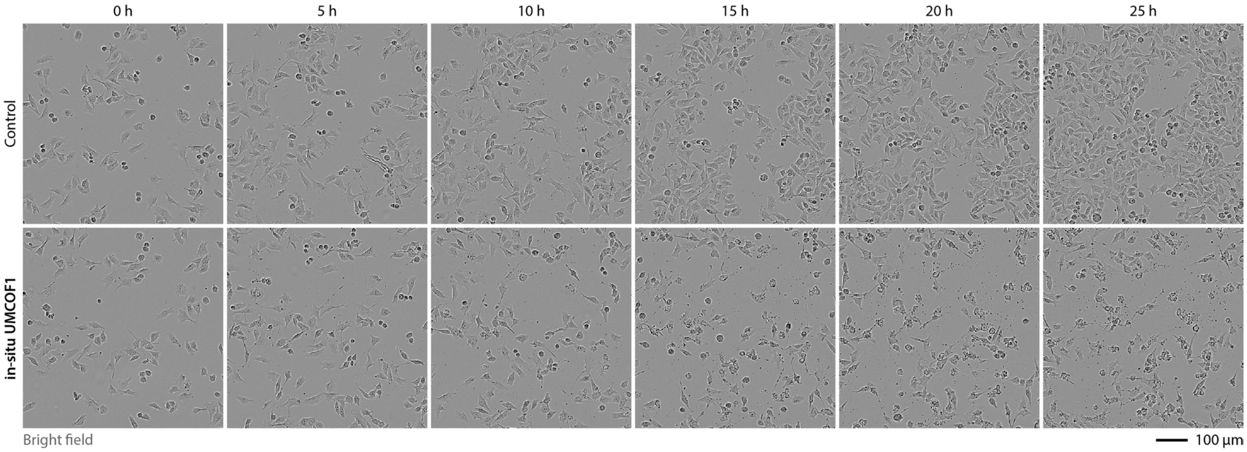

Figure S11. Real-time live-cell imaging for monitoring cellular growth of 4T1 cells co-treated with TAPB (50 µM) and DMTP (75 µM).

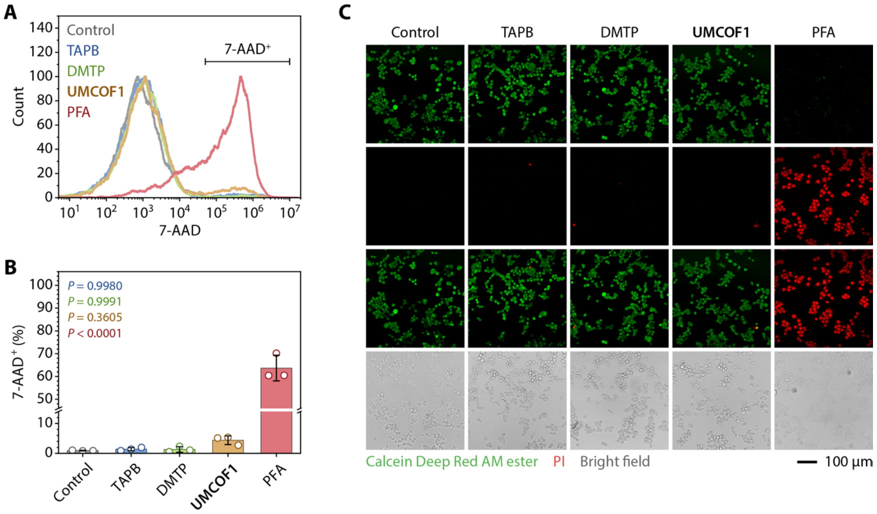

Figure S12. Cytotoxicity of TAPB, DMTP, and pre-synthesised UMCOF1 nanoparticles.

(A, B) Representative flow cytometric analysis (A) and 7-AAD$^+$ dead cell percentage (B) of 4T1 cells treated with TAPB (50 µM, 24 h), DMTP (75 µM, 24 h), pre-synthesised **UMCOF1** (200 µg/mL, 24 h), or PFA (4 wt%, 10 min). Data are expressed as mean ± SD, $n = 3$ biological replicates. Statistical significance was calculated using one-way ANOVA followed by Dunnett's *post hoc* tests.

(C) Representative confocal laser scanning fluorescence micrographs of 4T1 cells treated with treated with TAPB (50 µM, 24 h), DMTP (75 µM, 24 h), pre-synthesised **UMCOF1** (200 µg/mL, 24 h), or PFA (4 wt%, 24 h). Calcein Deep Red AM ester$^+$ PI$^-$ cells are considered living cells.

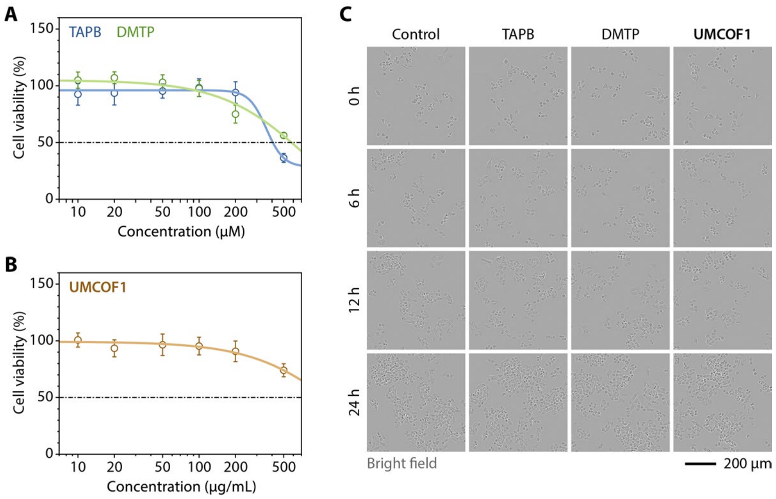

Figure S13. Changes in cell proliferation caused by TAPB, DMTP, and pre-synthesised UMCOF1 nanoparticles.

(A, B) CCK-8 viability assays of 4T1 cells treated with TAPB (0–500 μM), DMTP (0–500 μM), or pre-synthesised **UMCOF1** (0–0.5 mg/mL) for 24 h. Data are expressed as mean ± SD, $n = 4$ technical replicates.

(C) Real-time live-cell imaging for monitoring cellular growth of 4T1 cells treated with TAPB (50 μM), DMTP (75 μM), or pre-synthesised **UMCOF1** (200 μg/mL).

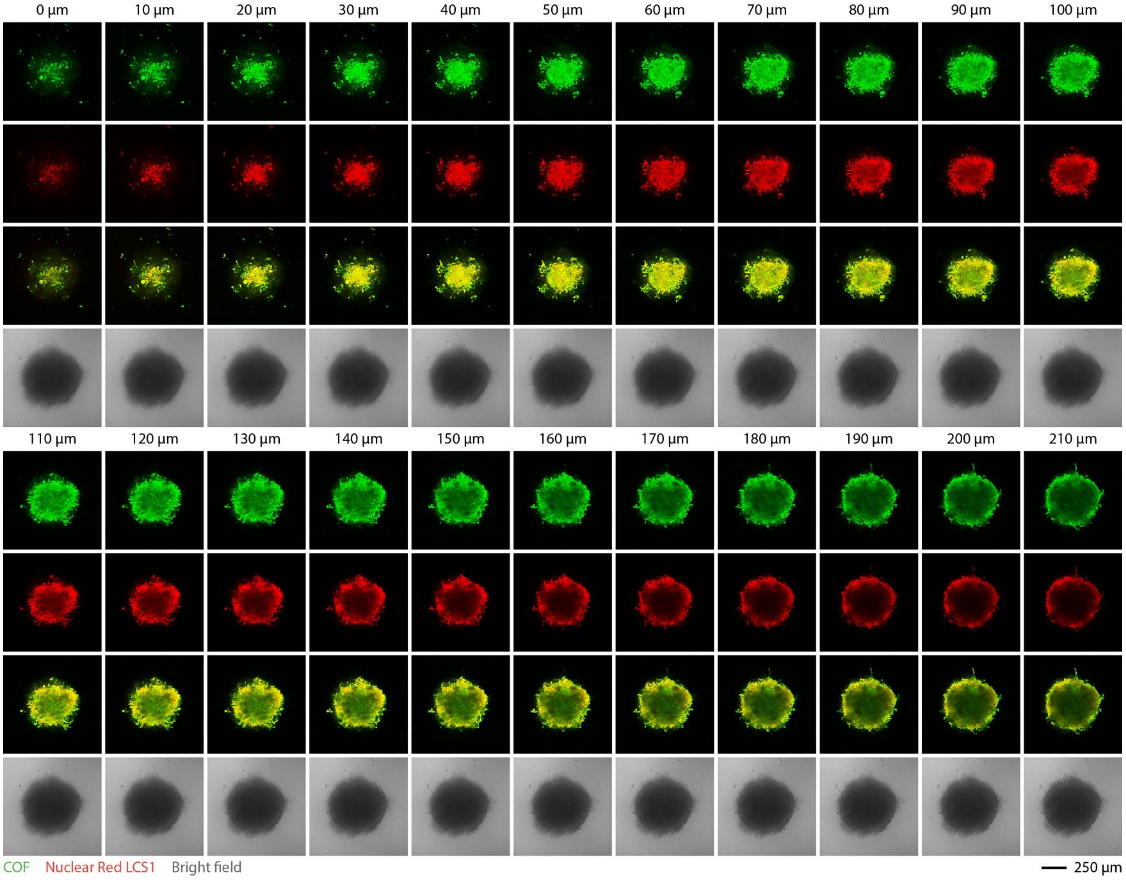

Figure S14. Representative confocal laser scanning tomographic fluorescence micrographs of a 4T1 multicellular tumour spheroid co-treated with TAPB (20 µM), DMTP (30 µM), and Nuclear Red LCS1 (1.0 µM) for 4 h.

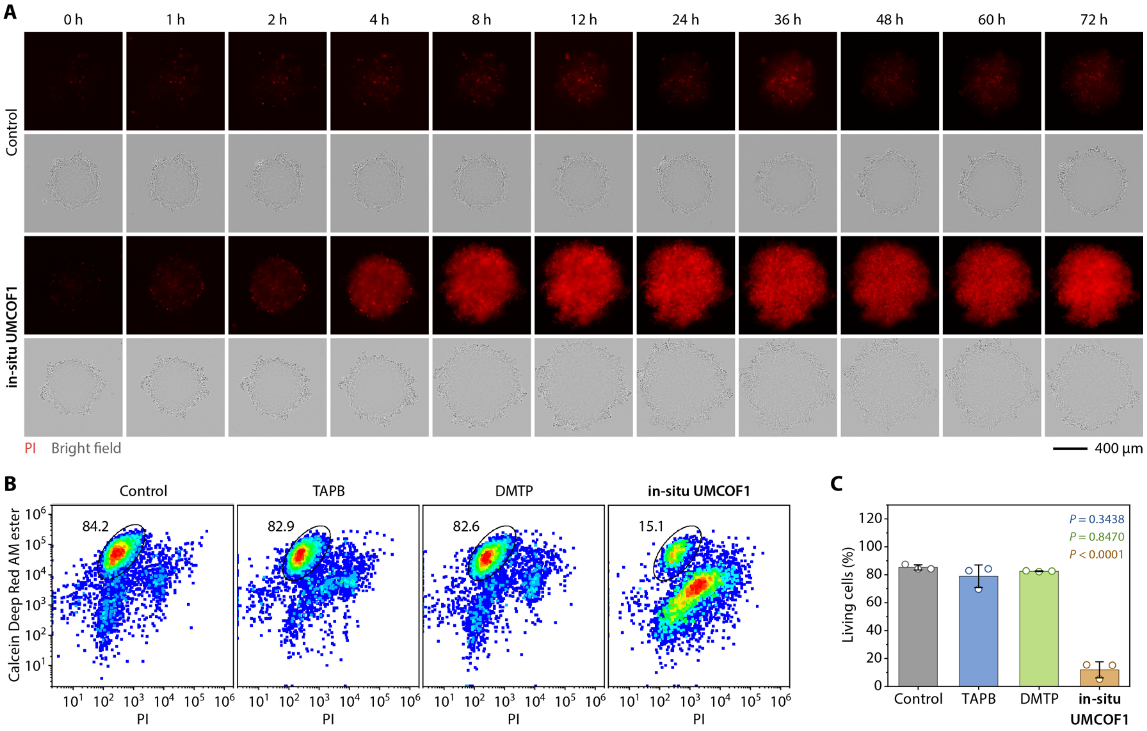

Figure S15. Cell death in 4T1 multicellular tumour spheroids due to intracellular synthesis of in-situ UMCOF1.

(A) Real-time live-cell imaging for monitoring cellular growth of a 4T1 multicellular tumour spheroid co-treated with TAPB (100 μM), DMTP (150 μM), and PI (5.0 μM) for 0–72 h. PI-positive cells are considered dead.

(B, C) Flow cytometry (B) performed on 4T1 multicellular tumour spheroids treated with TAPB (100 μM) and DMTP (150 μM) for 24 h using Calcein Deep Red AM ester and PI staining. Calcein Deep Red AM ester$^+$ PI$^-$ cells are considered living cells (C). Data are expressed as mean ± SD, n = 3 biological replicates. Statistical significance was calculated using one-way ANOVA followed by Dunnett's multiple comparison tests.

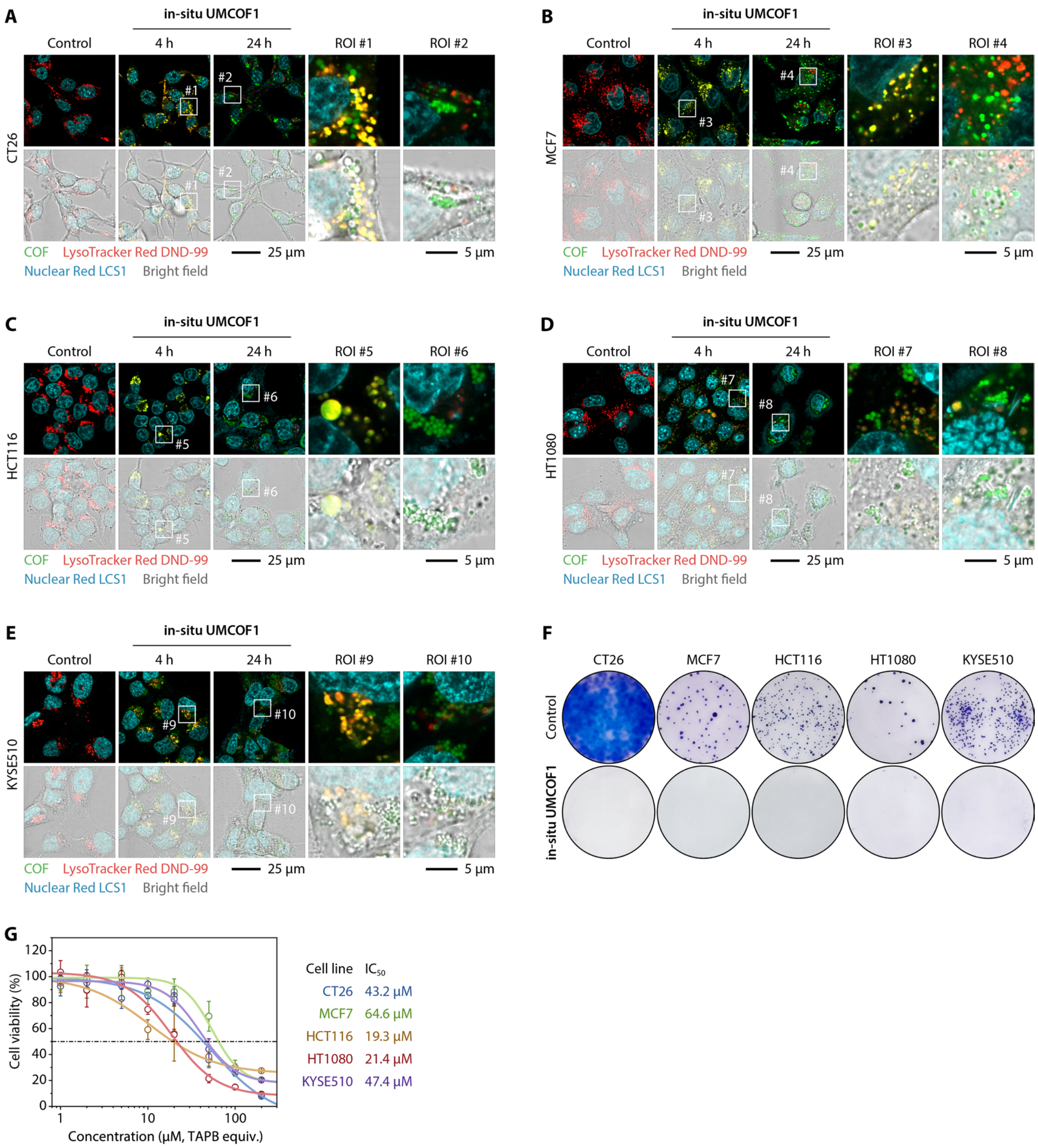

Figure S16. Synthesis of in-situ UMCOF1 in CT26, MCF7, HCT116, HT1080, and KYSE510 cells.

(A–E) Representative confocal laser scanning fluorescence micrographs of CT26 (A), MCF7 (B), HCT116 (C), HT1080 (D), and KYSE510 (E) cells treated with TAPB (20 µM for HCT116 and HT1080 cells; 50 µM for CT26, MCF7, and KYSE510 cells) and DMTP (30 µM for HCT116 and HT1080 cells; 75 µM for CT26, MCF7, and KYSE510 cells).

(F) Clonogenic assays of CT26, MCF7, HCT116, HT1080, and KYSE510 cells treated with TAPB (100 µM) and DMTP (150 µM) for 24 h.

(G) CCK-8 viability assays of CT26, MCF7, HCT116, HT1080, and KYSE510 cells treated with TAPB (0–200 µM) and DMTP (0–300 µM) for 24 h. Data are expressed as mean ± SD, $n = 4$ technical replicates. The fitted curves were based on the logistic model.

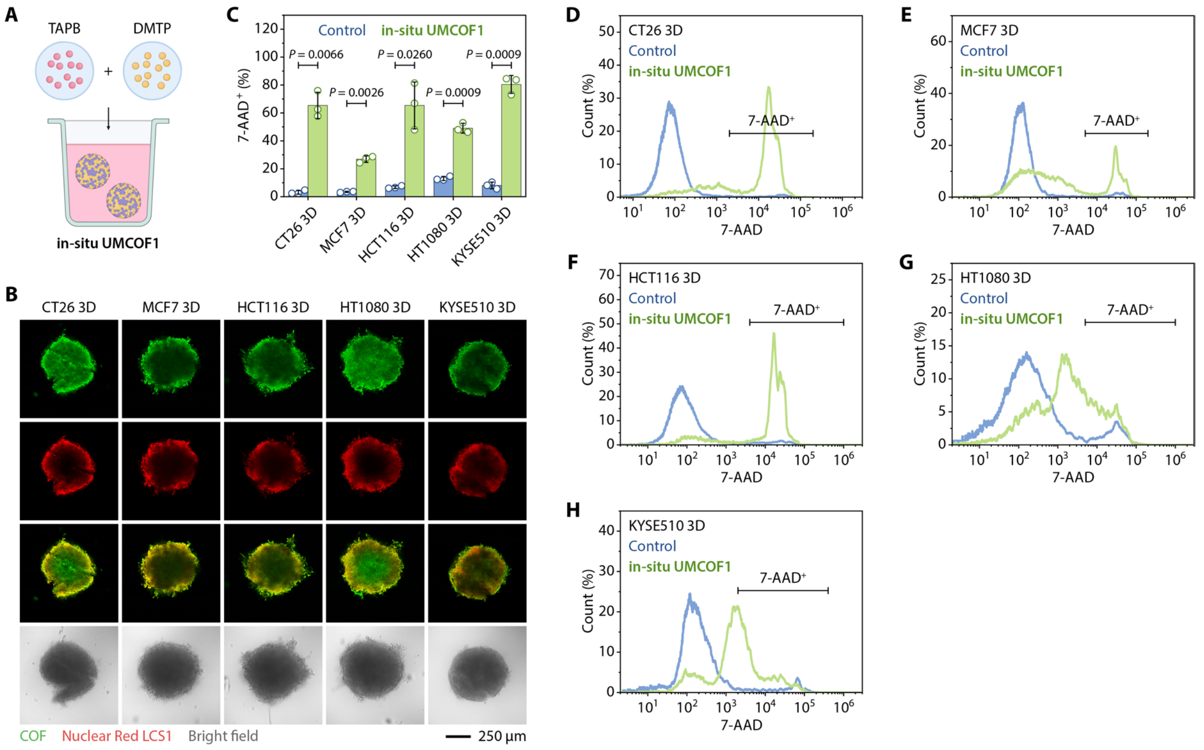

Figure S17. Synthesis of in-situ UMCOF1 in CT26, MCF7, HCT116, HT1080, and KYSE510 multicellular tumour spheroids.

(A) Schematic diagram of **in-situ UMCOF1** synthesis and imaging in multicellular tumour spheroids. Schematic created using BioRender.com.

(B) Representative confocal laser scanning fluorescence micrographs at the largest diameter of multicellular tumour spheroids co-treated with TAPB (20 µM), DMTP (30 µM), and Nuclear Red LCS1 (1.0 µM) for 6 h.

(C–H) Flow cytometry performed on CT26 (D), MCF7 (E), HCT116 (F), HT1080 (G), and KYSE510 (H) multicellular tumour spheroids treated with TAPB (100 µM) and DMTP (150 µM) for 24 h using 7-AAD (5.0 µM) staining. 7-AAD-positive cells are considered dead cells (C). Data are expressed as mean ± SD, n = 3 biological replicates. Statistical significance was calculated using two-tailed unpaired t test with Welch’s correction.

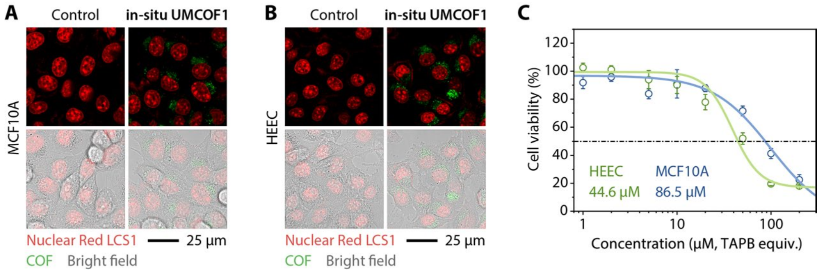

Figure S18. Synthesis of in-situ UMCOF1 inside MCF10A and HEEC immortalised normal cells.

(A) Representative confocal laser scanning fluorescence micrographs of MCF10A cells treated with TAPB (100 µM) and DMTP (150 µM) for 6 h.

(B) Representative confocal laser scanning fluorescence micrographs of HEEC cells treated with TAPB (50 µM) and DMTP (75 µM) for 6 h.

(C) CCK-8 viability assays of MCF10A and HEEC cells treated with TAPB (0–200 µM) and DMTP (0–300 µM) for 24 h. Data are expressed as mean ± SD, $n = 4$ technical replicates. The fitted curves were based on the logistic model.

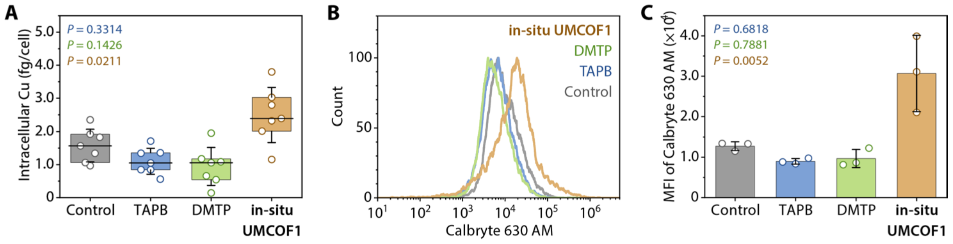

Figure S19. Intracellular Cu^{2+} and Ca^{2+} contents of 4T1 cells.

(A) ICP–MS for detecting Cu contents in 4T1 cells after treatment with TAPB (50 μM) and DMTP (75 μM) for 24 h. The middle line in the boxplot indicates the median, the whiskers represent SD, and the lower and upper hinges correspond to the first and third quartiles, respectively. *n* = 7 biological replicates.

(B, C) Representative flow cytometric analysis (B) and the quantification (C) of Calbryte 630 AM staining for detecting Ca^{2+} levels in 4T1 cells after treatment with TAPB (50 μM) and DMTP (75 μM) for 24 h. Data are expressed as mean ± SD, *n* = 3 biological replicates.

Statistical significance was calculated using one-way ANOVA followed by Dunnett's multiple comparison tests (A, C).

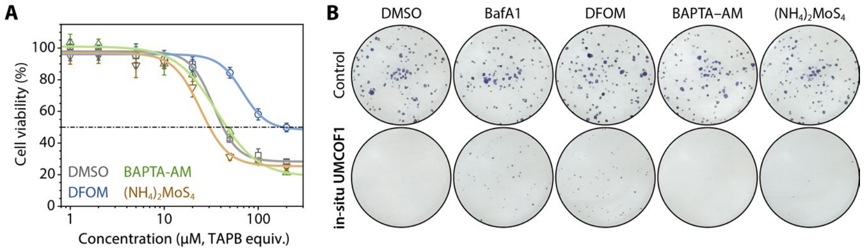

Figure S20. Metal ion-related cell death of 4T1 cells.

(A) Cell death rescue experiments of 4T1 cells after treatment with TAPB (50 μM) and DMTP (75 μM) in the absence or presence of DMSO (0.1 vol%), BAPTA-AM (1.0 μM), DFOM (100 μM), and $(NH_4)_2MoS_4$ (10 μM). Data are expressed as mean ± SD, $n = 4$ technical replicates. The fitted curves were based on the logistic model.

(B) Clonogenic assays of 4T1 cells treated with TAPB (50 μM) and DMTP (75 μM) in the absence or presence of DMSO (0.1 vol%), BafA1 (50 nM), DFOM (100 μM), and $(NH_4)_2MoS_4$ (10 μM) for 24 h.

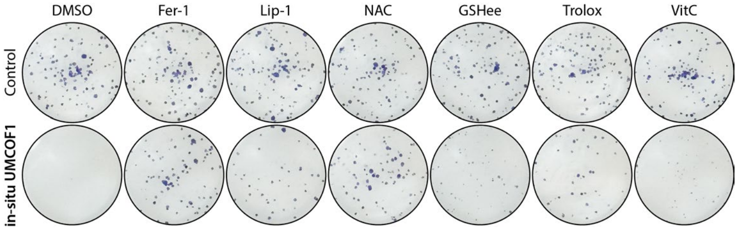

Figure S21. Clonogenic assays of 4T1 cells treated with TAPB (50 μM) and DMTP (75 μM) in the absence or presence of DMSO (0.1 vol%), Fer-1 (1.0 μM), Lip-1 (0.5 μM), NAC (5.0 mM), GSHee (2.0 mM), Trolox (100 μM), VitC (100 μM) for 24 h.

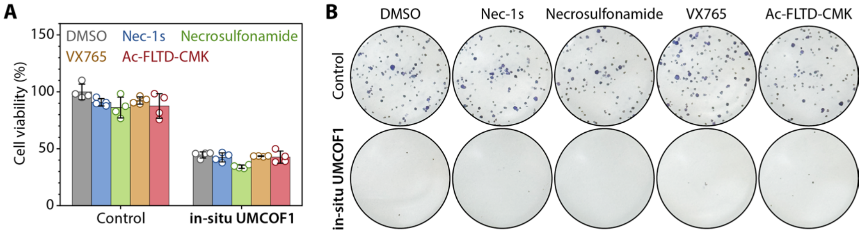

Figure S22. Necroptosis- and pyroptosis-independent cell death of 4T1 cells.

(A) Cell death rescue experiments of 4T1 cells after treatment with TAPB (50 μM) and DMTP (75 μM) in the absence or presence of DMSO (0.1 vol%), Nec-1s (5.0 μM), necrosulfonamide (1.0 μM), VX765 (20 μM), and Ac-FLTD-CMK (10 μM). Data are expressed as mean ± SD, *n* = 4 technical replicates.

(B) Clonogenic assays of 4T1 cells treated with TAPB (50 μM) and DMTP (75 μM) in the absence or presence of DMSO (0.1 vol%), Nec-1s (5.0 μM), necrosulfonamide (1.0 μM), VX765 (20 μM), and Ac-FLTD-CMK (10 μM) for 24 h.

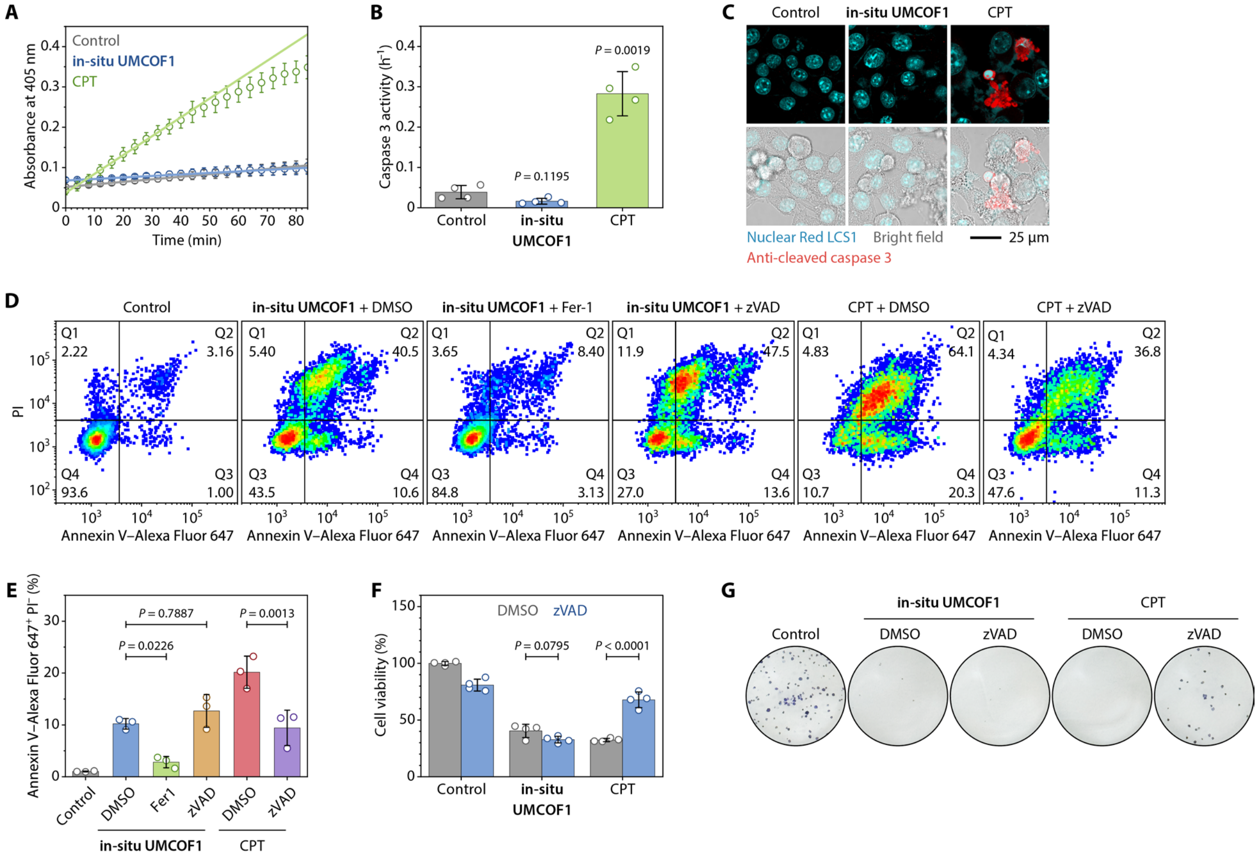

Figure S23. Apoptosis-independent cell death of the 4T1 cells.

(A, B) Caspase 3 activation (A) of the 4T1 cells treated with TAPB (50 μM) and DMTP (75 μM) for 24 h, reflected by the increased absorbance rate at 405 nm (B). *n* = 4 biological replicates.

(C) Immunofluorescence staining of cleaved caspase 3 in the 4T1 cells treated with TAPB (50 μM) and DMTP (75 μM) for 24 h.

(D, E) Representative flow cytometric analysis based on Annexin V–Alexa Fluor 647 and PI double staining (D), and the percentages of apoptotic cells (E) of the 4T1 cells treated with TAPB (50 μM) and DMTP (75 μM) in the absence or presence of DMSO (0.1 vol%), Fer-1 (1.0 μM), zVAD (25 μM) for 24 h. *n* = 3 biological replicates.

(F) Cell death rescue experiments of the 4T1 cells after treatment with TAPB (50 μM) and DMTP (75 μM) in the absence or presence of DMSO (0.1 vol%) and zVAD (25 μM). *n* = 4 technical replicates.

(G) Clonogenic assays of the 4T1 cells treated with TAPB (50 μM) and DMTP (75 μM) in the absence or presence of DMSO (0.1 vol%) and zVAD (25 μM) for 24 h.

Cells treated with CPT (2.0 μM) for 24 h were used as the positive control for apoptosis. Data are expressed as mean ± SD (A, B, E, F). Statistical significance was calculated using Welch's ANOVA followed by Dunnett's T3 multiple comparison test (B), one-way ANOVA followed by Tukey's multiple comparison test (E), and two-way ANOVA followed by Šídák's multiple comparison test (F).

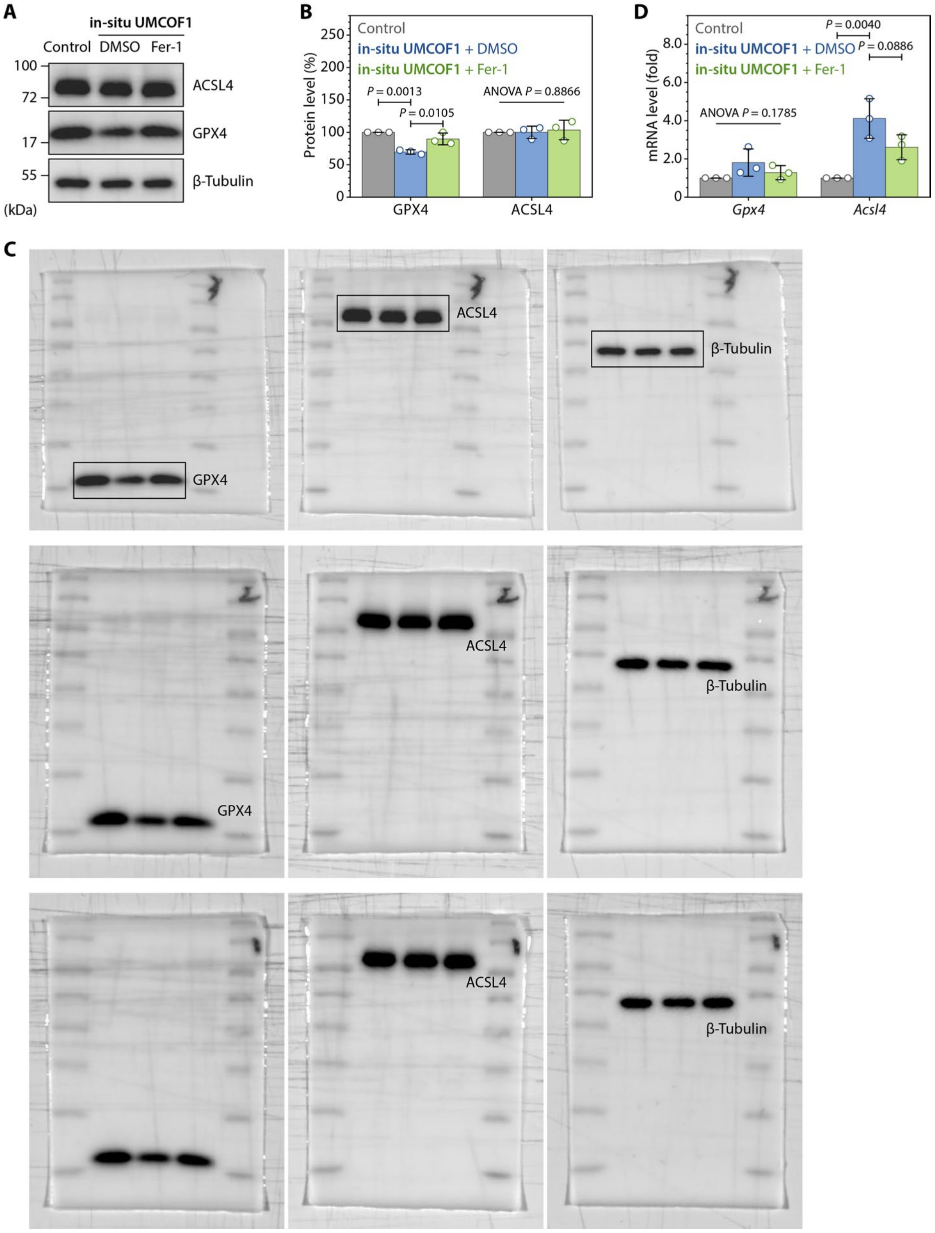

Figure S24. Ferroptosis-related protein and mRNA levels of 4T1 cells after treatment with TAPB (50 μM) and DMTP (75 μM) for 24 h.

(A–C) Representative Western blots of GPX4 and ACSL4 (A) and statistical data (B) of the treated 4T1 cells in the absence or presence of DMSO (0.1 vol%) and Fer-1 (1.0 μM). *n* = 3 biological replicates using cells from different passages (C).

(D) *Gpx4* and *Acsl4* mRNA levels as measured using RT-qPCR in the treated 4T1 cells in the absence or presence of DMSO (0.1 vol%) and Fer-1 (1.0 μM). *n* = 3 biological replicates using cells from different passages.

Data are expressed as mean ± SD (B, D). Statistical significance was calculated using one-way ANOVA followed by Tukey's multiple comparison tests (B, D).

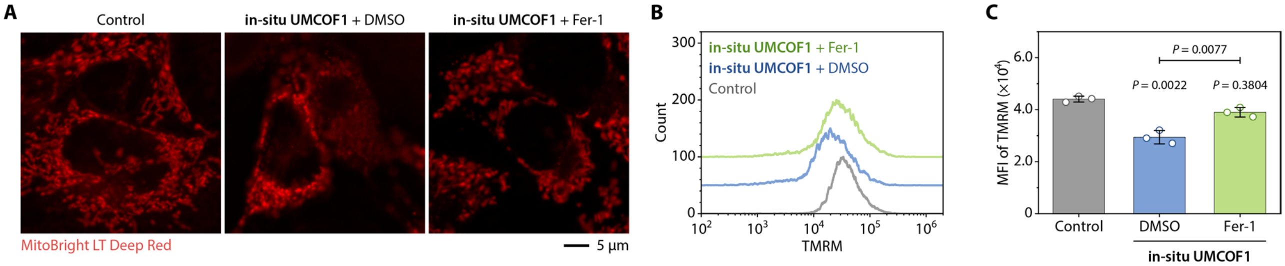

Figure S25. Mitochondrial damage associated with ferroptosis of 4T1 cells after treatment with TAPB (50 μM) and DMTP (75 μM) for 24 h.

(A) Confocal laser scanning fluorescence micrographs of MitoBright LT Deep Red-staining mitochondria in the treated 4T1 cells in the absence or presence of DMSO (0.1 vol%) and Fer-1 (1.0 μM).

(B, C) Representative flow cytometric analysis (B) and the quantification (C) of TMRM staining for revealing mitochondrial membrane potential changes in the treated 4T1 cells in the absence or presence of DMSO (0.1 vol%) and Fer-1 (1.0 μM). Data are expressed as mean ± SD. $n = 3$ biological replicates. Statistical significance was calculated using one-way ANOVA followed by Tukey's multiple comparison tests

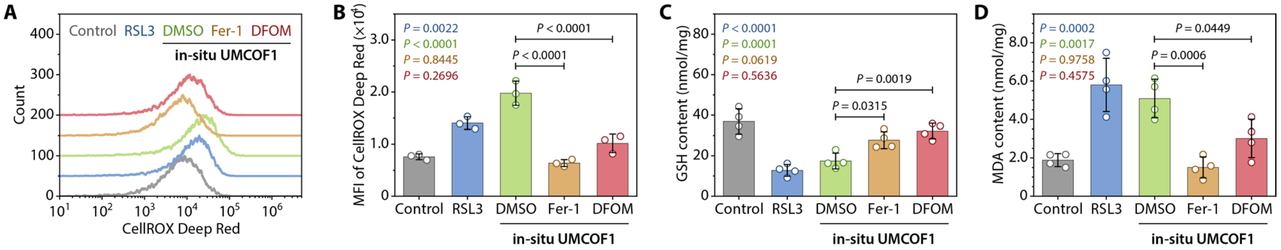

Figure S26. Oxidative stress detection of 4T1 cells.

(A, B) Representative flow cytometric analysis (A) and the data quantification (B) of total ROS using the CellROX Deep Red fluorescence probe in 4T1 cells after treatment with TAPB (50 μM) and DMTP (75 μM) in the absence or presence of DMSO (0.1 vol%), Fer-1 (1.0 μM), and DFOM (100 μM) for 24 h. *n* = 3 biological replicates.

(C, D) GSH (C) and MDA (D) levels of 4T1 cells after treatment with TAPB (50 μM) and DMTP (75 μM) in the absence or presence of DMSO (0.1 vol%), Fer-1 (1.0 μM), and DFOM (100 μM) for 24 h. *n* = 4 biological replicates.

Cells treated with RSL3 (0.5 μM, 24 h) were used as the positive control of ferroptosis. Data are expressed as mean ± SD (B, C, D). Statistical significance was calculated using one-way ANOVA followed by Tukey's multiple comparison tests (B, C, D).

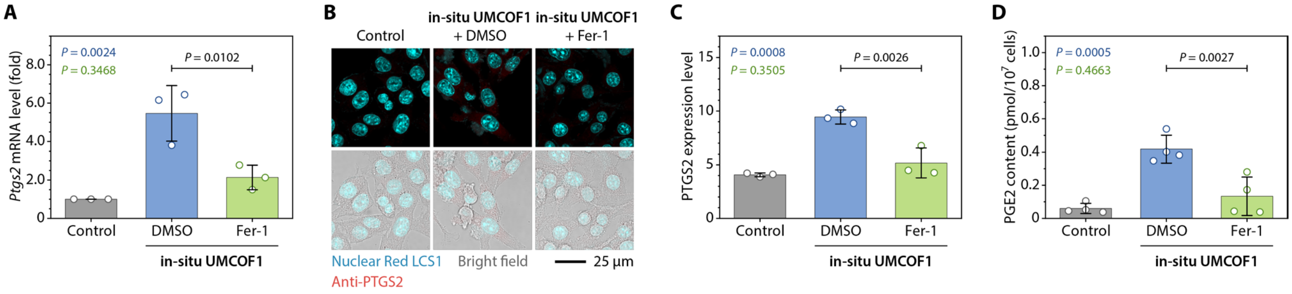

Figure S27. PTGS2 expression and PGE2 contents of 4T1 cells.

(A) *Ptgs2* mRNA levels measured using RT-qPCR in 4T1 cells treated with TAPB (50 μM) and DMTP (75 μM) in the absence or presence of DMSO (0.1 vol%) and Fer-1 (1.0 μM). *n* = 3 biological replicates using cells from different passages.

(B, C) Immunofluorescence staining (B) and cellular MFI (C) of PTGS2 in 4T1 cells treated with TAPB (50 μM) and DMTP (75 μM) in the absence or presence of DMSO (0.1 vol%) and Fer-1 (1.0 μM) for 24 h. *n* = 3 biological replicates.

(D) PGE2 contents of 4T1 cells after treatment with TAPB (50 μM) and DMTP (75 μM) in the absence or presence of DMSO (0.1 vol%) and Fer-1 (1.0 μM) for 24 h. *n* = 4 biological replicates.

Data are expressed as mean ± SD (A, C, D). Statistical significance was calculated using one-way ANOVA followed by Tukey's multiple comparison tests (A, C, D).

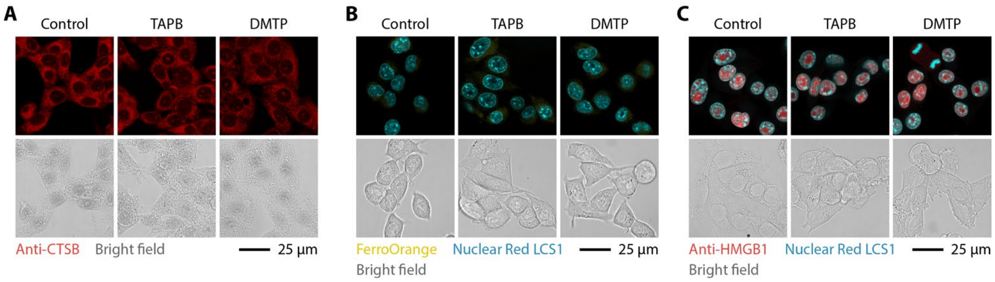

Figure S28. CTSB, Fe^{2+}, and HMGB1 levels of 4T1 cells treated with TAPB (50 μM) or DMTP (75 μM) for 24 h.

(A) CTSB immunofluorescence staining of the methanol-fixed 4T1 cells.

(B) Detection of intracellular Fe(II) with the FerroOrange fluorescence probe.

(C) HMGB1 immunofluorescence staining of the PFA-fixed 4T1 cells.

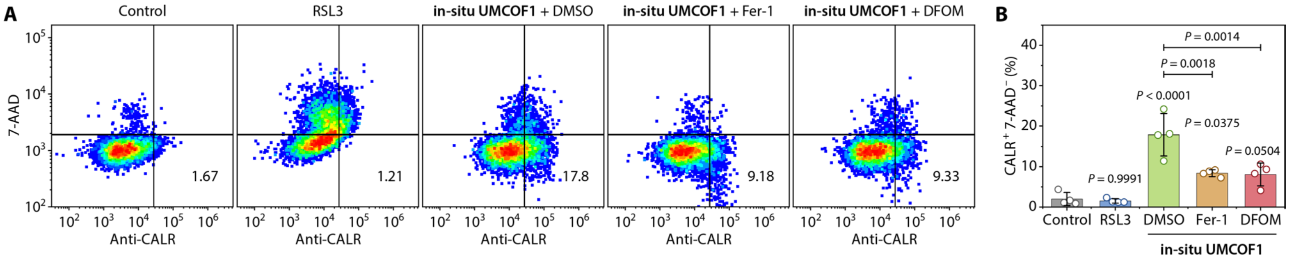

Figure S29. CALR membrane expression of the 4T1 cells treated with TAPB (50 μM) and DMTP (75 μM) in the absence or presence of DMSO (0.1 vol%), Fer-1 (1.0 μM), DFOM (100 μM) for 24 h.

(A) Representative flow cytometric analysis based on CALR immunofluorescence staining.

(B) The percentages of the cells with CALR membrane expression.

The 4T1 cells treated with RSL3 (0.5 μM, 24 h) were used as the positive control of ferroptosis. Data are expressed as mean ± SD. *n* = 4 biological replicates. Statistical significance was calculated using one-way ANOVA followed by Tukey's multiple comparison tests.

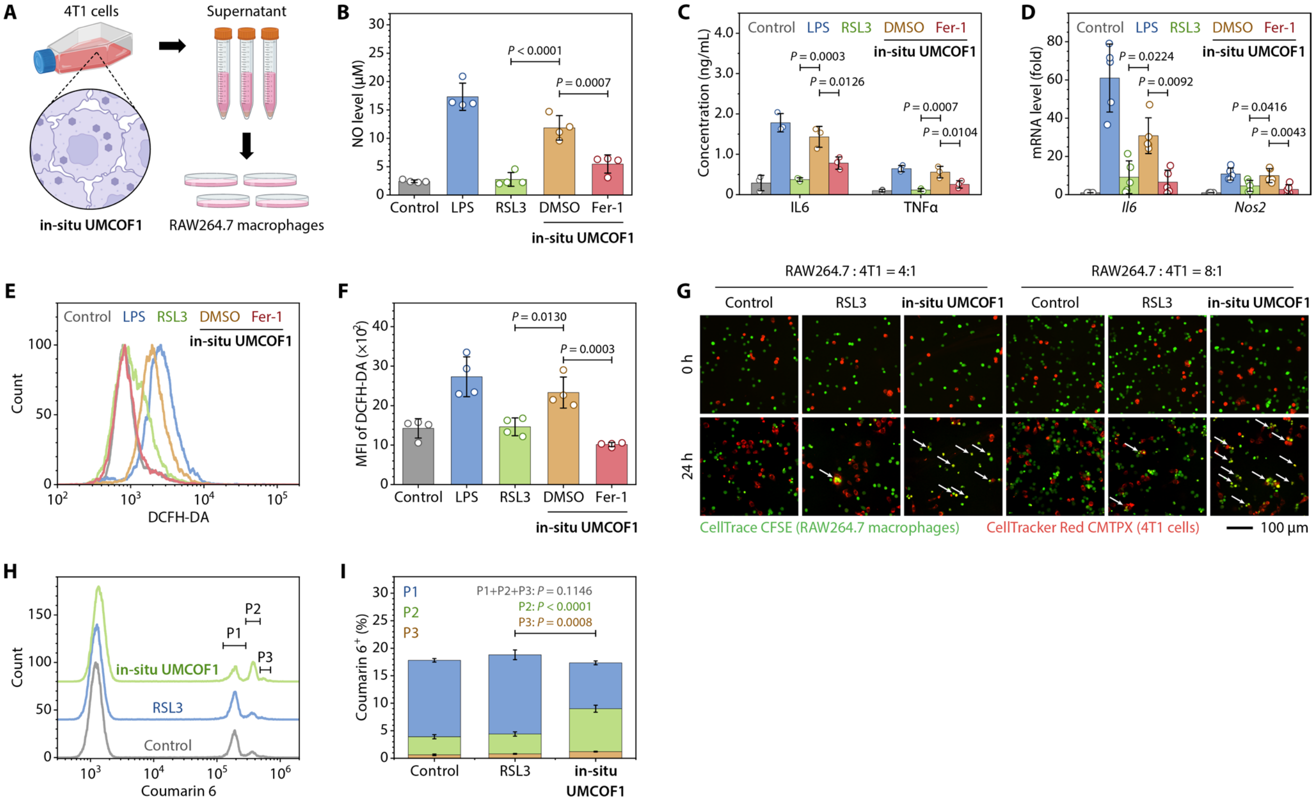

Figure S30. Ferroptotic 4T1 cells for promoting classical activation of RAW264.7 macrophages.

(A) Experimental design for RAW264.7 classical activation induced by the culture supernatant of the 4T1 cells after the treatment with TAPB (50 μM) and DMTP (75 μM).

(B) NO concentration in the culture supernatant of the differently treated RAW264.7 macrophages quantified by the Griess reaction. $n = 4$ biological replicates.

(C) Concentrations of inflammatory cytokines released by the differently treated RAW264.7 macrophages. $n = 3$ biological replicates.

(D) *Il6* and *Nos2* mRNA levels measured by RT-qPCR in the differently treated RAW264.7 macrophages. $n = 5$ biological replicates.

(E, F) Representative flow cytometric analysis (E) and the corresponding quantification (F) of DCFH-DA staining for detecting total ROS in the differently treated RAW264.7 macrophages. $n = 4$ biological replicates.

(G) Intercellular interactions between the 4T1 cells (red) and the differently treated RAW264.7 macrophages (green) at co-culture densities of 4:1 and 8:1.

(H, I) Representative flow cytometric analysis (H) and quantification (I) revealing the phagocytosis of fluorescently labelled polystyrene microspheres by differently treated RAW264.7 macrophages. $n = 4$ biological replicates.

The 4T1 cells treated with RSL3 (0.5 μM, 24 h) were used as the positive control of ferroptosis. RAW264.7 macrophages treated with LPS (100 ng/mL, 24 h) were used as positive controls for classical activation. Data are expressed as mean ± SD (B, C, D, F, I). Statistical significance was calculated using one-way ANOVA followed by Tukey's multiple comparison test (B, C, D, F, I). Schematics created using BioRender.com (A).

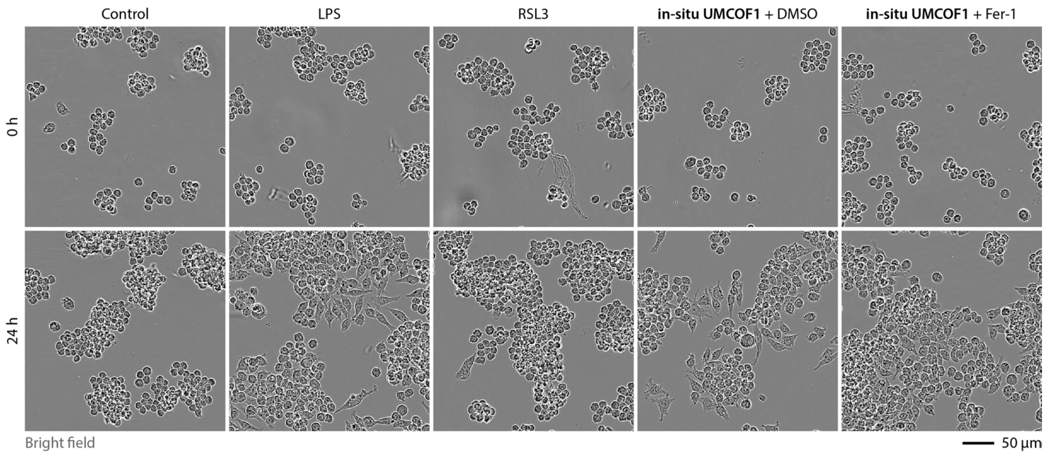

Figure S31. Morphological changes of RAW264.7 macrophages after different treatments.

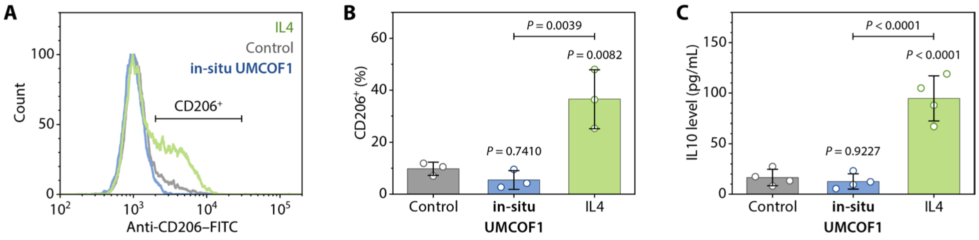

Figure S32. Alternative activation of RAW264.7 macrophages after different treatments.

(A) Representative flow cytometric analysis of CD206 expression.

(B) The percentages of CD206-positive macrophages. $n = 3$ biological replicates.

(C) Released IL10 levels. $n = 4$ biological replicates.

Data are expressed as mean ± SD (B, C). Statistical significance was calculated using one-way ANOVA followed by Tukey's multiple comparison tests (B, C).

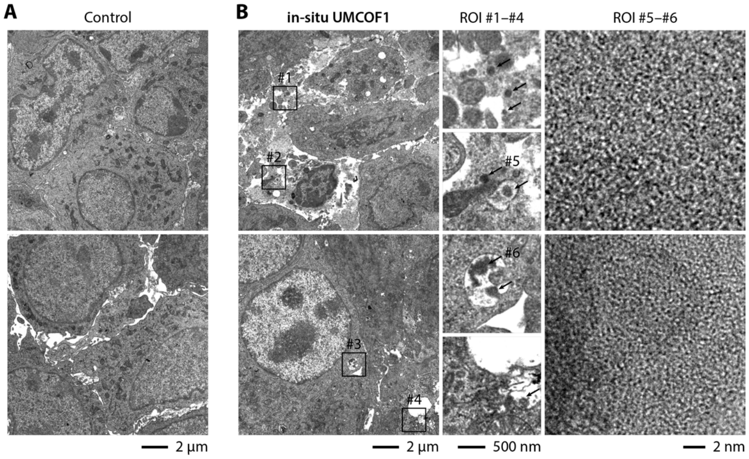

Figure S33. TEM-imaging of in-situ UMCOF1 in 4T1 tumours.

(A) Bio-TEM images of tumours excised from mice with intratumour injection of Tween-80 (0.05 vol%).

(B) Bio-TEM images and local high-resolution images of tumours excised from mice with intratumour injection of TAPB (0.4 mM) and DMTP (0.6 mM). Black arrows indicate **in-situ UMCOF1** nanoparticles.

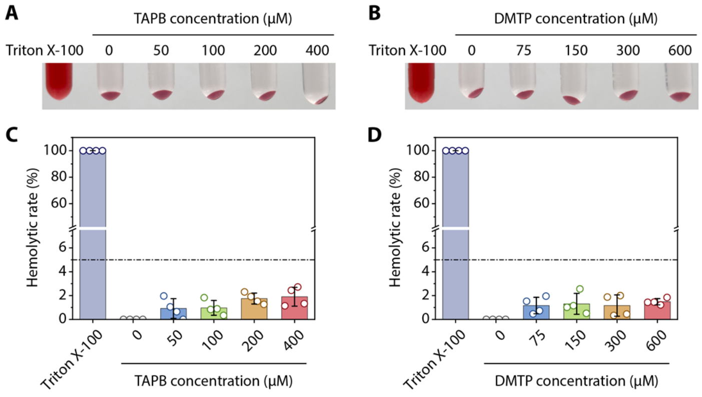

Figure S34. Hemolysis tests.

(A–D) Representative photographs (A, B) and Hemolytic rates (C, D) from hemolysis tests of red blood cells incubated with TAPB (A, C) and DMTP (B, D) at various concentrations for 1 h at 37°C. Triton X-100 (0.25 vol%) was used as a positive control (hemolytic rate, 100%) and PBS was used as a negative control (hemolytic rate, 0%). Data are expressed as mean ± SD, $n = 4$ biological replicates.

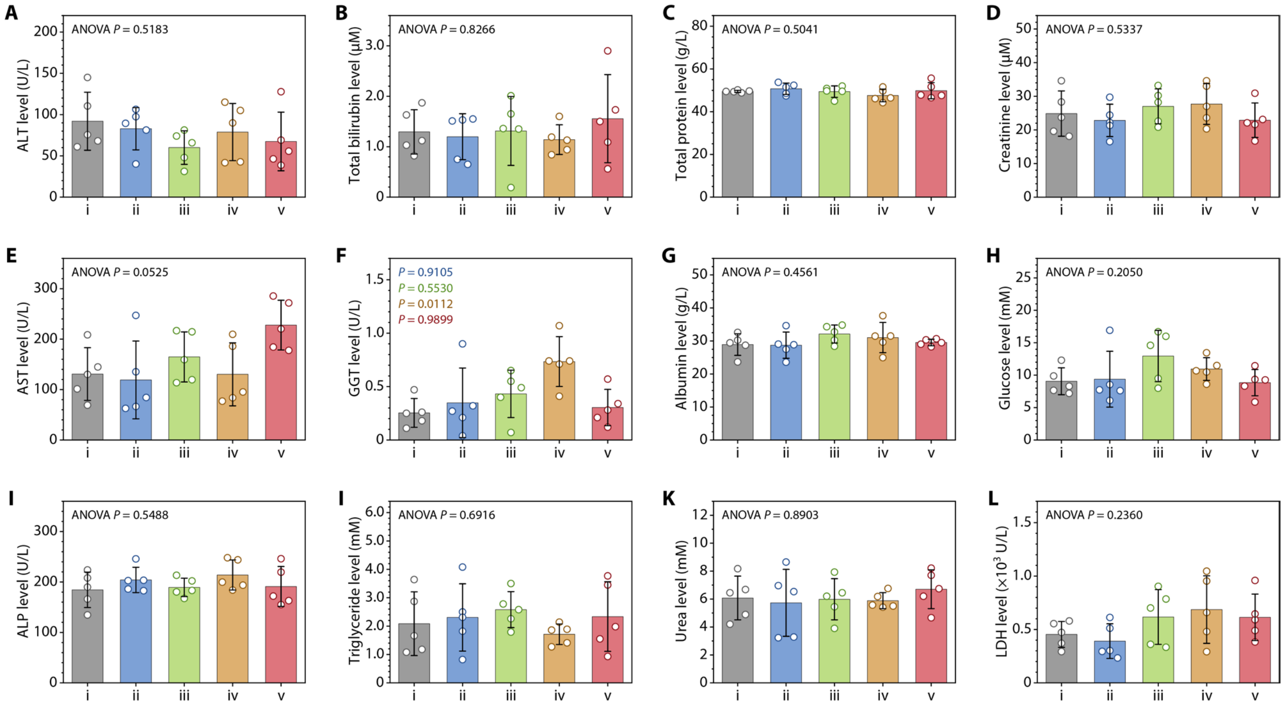

Figure S35. Blood biochemical analysis of BALB/c mice with unilateral 4T1 tumours at Day 15.

(A) Alanine transaminase (ALT).

(B) Total bilirubin.

(C) Total protein.

(D) Creatinine.

(E) Aspartate aminotransferase (AST).

(F) γ-Glutamyl Transferase (GGT).

(G) Albumin.

(H) Glucose.

(I) Alkaline phosphatase (ALP).

(J) riglyceride.

(K) Urea.

(L) LDH.

Data are expressed as mean ± SD, n = 5 mice per group. Statistical significance was determined using one-way ANOVA (A–L) followed by Dunnett's multiple comparison test (F).

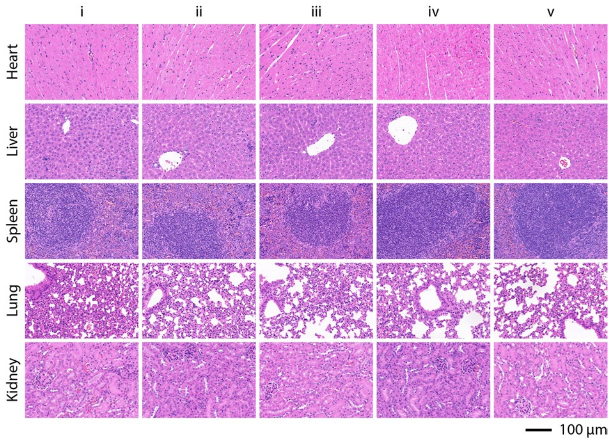

Figure S36. H&E histological analysis of major organs obtained from BALB/c mice with unilateral 4T1 tumours in different treatment groups at Day 15.

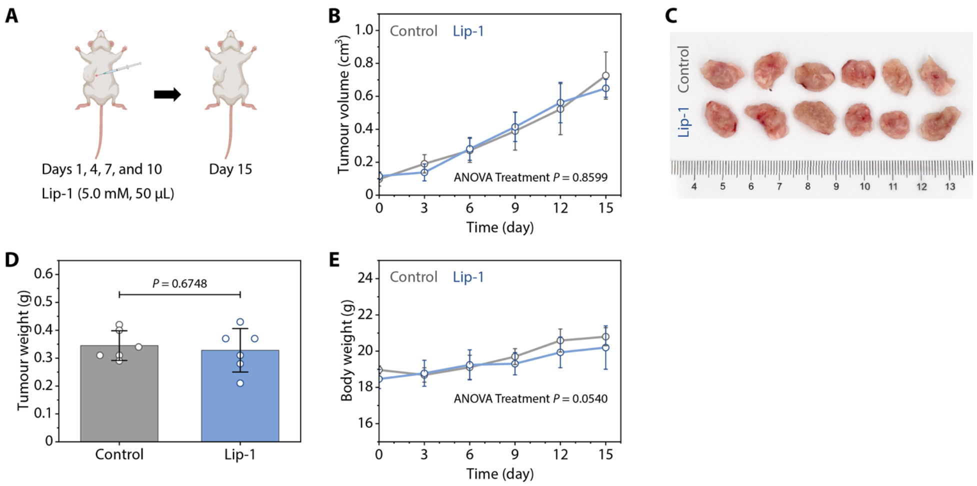

Figure S37. Tumour growth in 4T1 tumour-bearing BALB/c mice injected peritumourally with Lip-1.

(A) Therapeutic schedule.

(B) Tumour growth curves.

(C, D) Photographs (C) and weight (D) of tumours excised from mice at Day 15.

(E) Body weight changes.

Data are expressed as mean ± SD, n = 6 mice per group. Statistical significance was determined using two-way repeated measures ANOVA (B, E) and two-tailed unpaired t test (D). Schematic was created with BioRender.com (A).

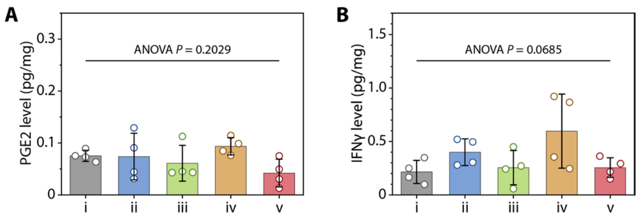

Figure S38. Intratumoural PGE2 and IFNγ levels of BALB/c mice with unilateral 4T1 tumours.

(A, B) PGE2 (A) and IFNγ (B) levels in tumours excised from BALB/c mice with unilateral tumours in different treatment groups at Day 15. Data are expressed as mean ± SD, $n = 4$ mice per group. Statistical significance was determined using one-way ANOVA.

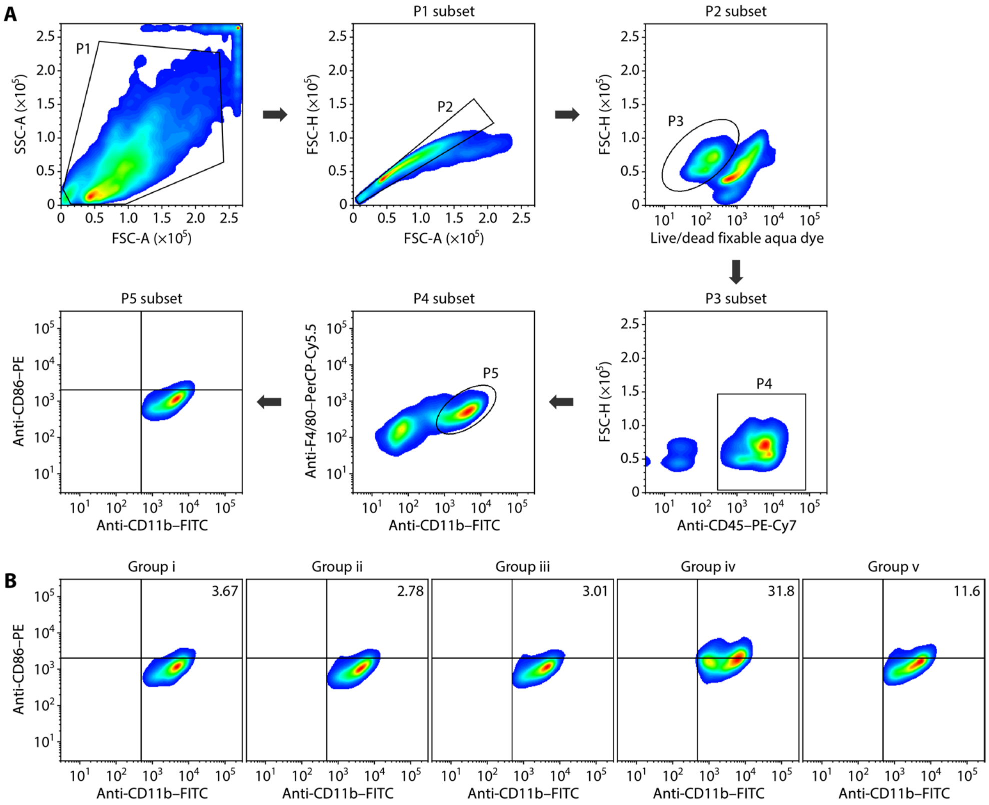

Figure S39. Flow cytometric analysis for classically activated macrophages in spleen of unilateral 4T1 tumour-bearing BALB/c mice (groups i–v).

(A) Gating strategy.

(B) Representative flow cytometric plots.

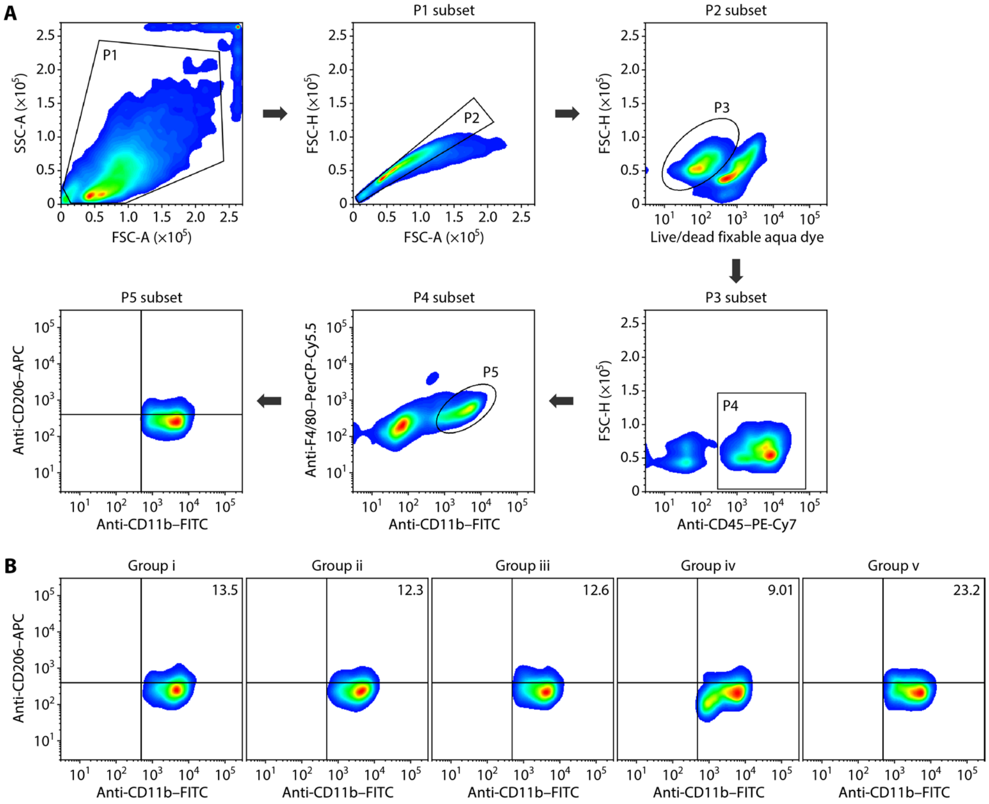

Figure S40. Flow cytometric analysis for alternatively activated macrophages in spleen of unilateral 4T1 tumour-bearing BALB/c mice (groups i–v).

(A) Gating strategy.

(B) Representative flow cytometric plots.

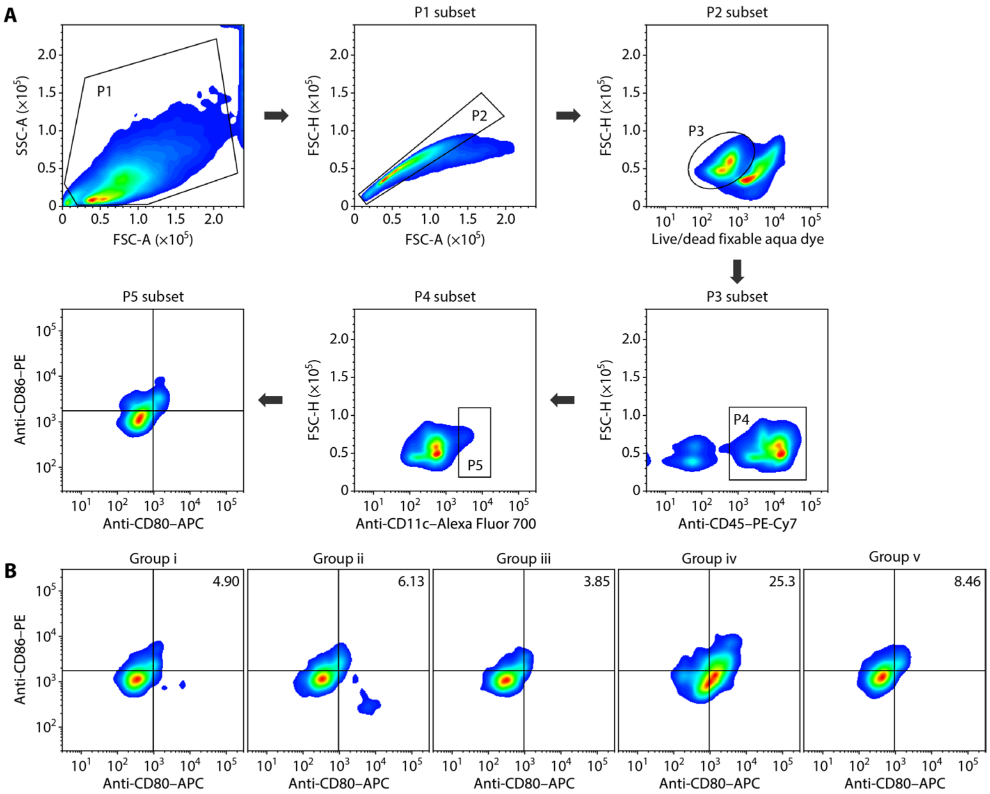

Figure S41. Flow cytometric analysis for maturated DCs in spleen of unilateral 4T1 tumour-bearing BALB/c mice (groups i–v).

(A) Gating strategy.

(B) Representative flow cytometric plots.

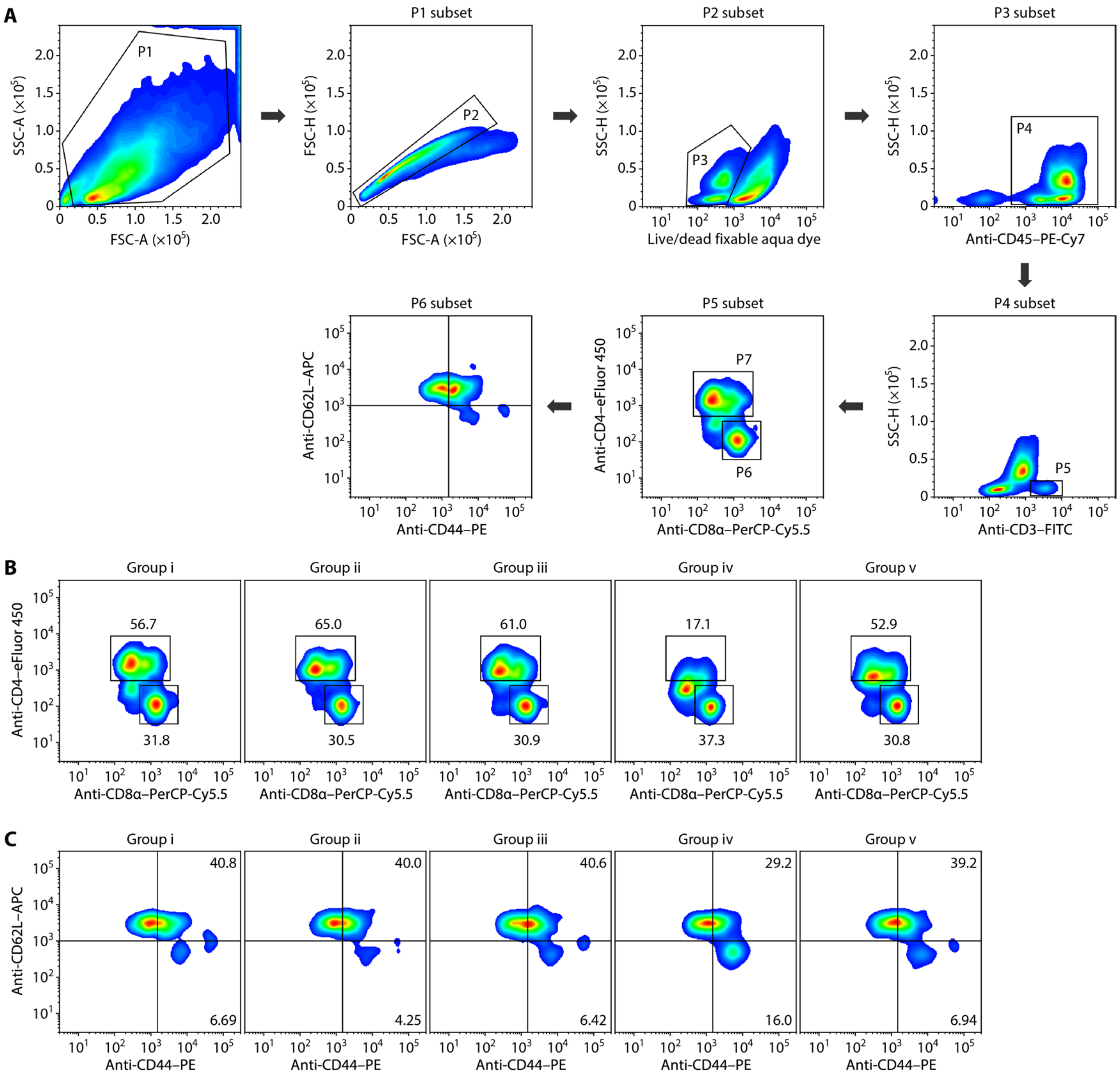

Figure S42. Flow cytometric analysis for T cells in spleen of unilateral 4T1 tumour-bearing BALB/c mice (groups i–v).

(A) Gating strategy.

(B) Representative flow cytometric plots for evaluating cytotoxic and helper T lymphocytes.

(C) Representative flow cytometric plots for evaluating memory T lymphocytes.

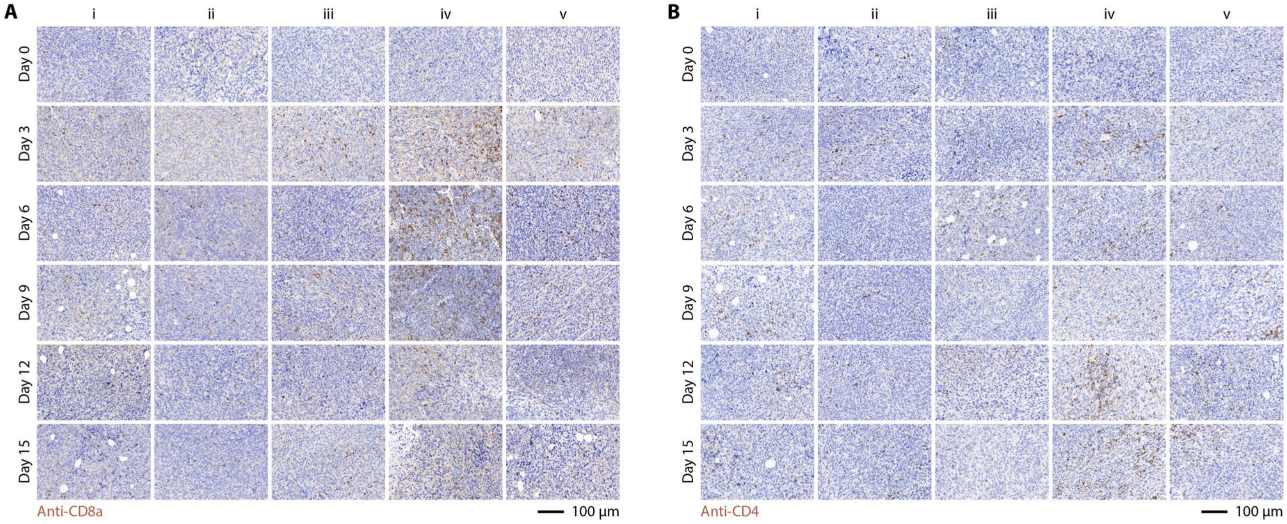

Figure S43. Time-dependent intratumour T-cell infiltration of unilateral 4T1 tumour-bearing BALB/c mice (groups i–v).

(A) Representative CD8a IHC staining of tumours obtained at Days 0, 3, 6, 9, 12, and 15.

(B) Representative CD4 IHC staining of tumours obtained at Days 0, 3, 6, 9, 12, and 15.

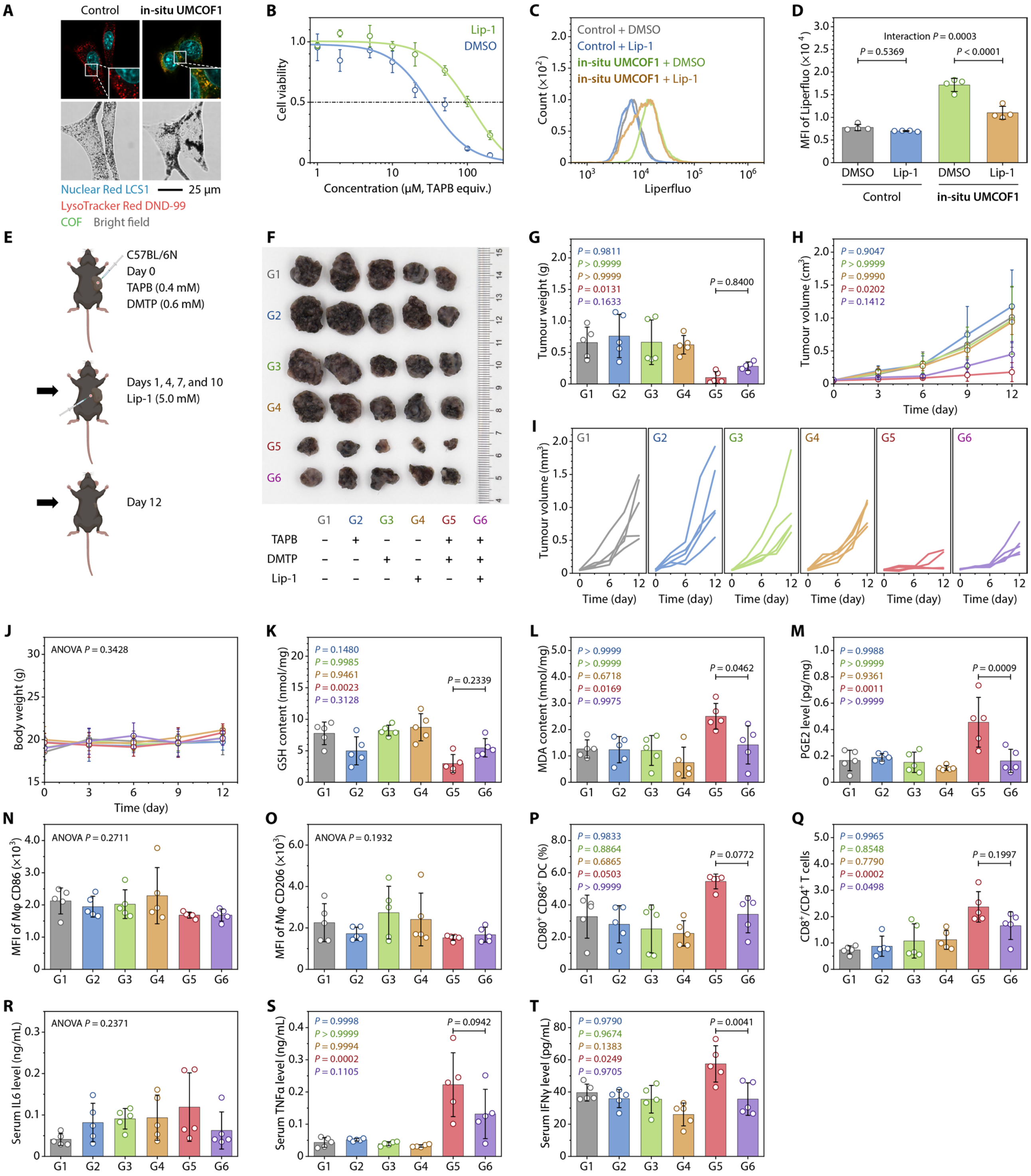

Figure S44. (See next page for caption)

Figure S44. Synthesis of in-situ UMCOF1 in B16F10 cells and B16F10 tumour of the C57BL/6N mice for immunotherapy.

(A) Representative confocal laser scanning fluorescence micrographs of the B16F10 cells treated with TAPB (20 μM) and DMTP (30 μM) for 6 h.

(B) CCK-8 viability assays of the B16F10 cells treated with TAPB (0–200 μM) and DMTP (0–300 μM) in the absence or presence of Lip-1 (0.5 μM) for 24 h.

(C, D) Representative flow cytometric analysis (C) and quantification (D) revealing lipid peroxidation in the B16F10 cells treated with TAPB (50 μM) and DMTP (75 μM) in the absence or presence of Lip-1 (0.5 μM) for 24 h.

(E) Therapeutic schedule involving a single intratumoural injection of a mixture (50 μL) of TAPB (0.4 mM) and DMTP (0.6 mM) as well as a peritumoural injection of Lip-1 (5.0 mM, 50 μL).

(F, G) Photographs (F) and weight (G) of tumours excised from mice in various groups at the treatment endpoint.

(H, I) Tumour volume changes (H) with different treatments (I).

(J) Body weight changes in mice subjected to different treatments.

(K–M) Intratumoural GSH (K), MDA (L), and PGE2 (M) levels of mice in various groups at the treatment endpoint.

(N, O) Quantification of CD86 (N) and CD206 (O) expressed on the surface of F4/80^{+} CD11b^{+} CD45^{+} spleen cells of mice from various groups at the treatment endpoint.

(P)Percentages of CD80^{+} CD86^{+} double-positive DCs in the CD11c^{+} CD45^{+} spleen cells of mice in various groups at the treatment endpoint.

(Q) Ratio of CD8^{+} T cells to CD4^{+} T cells in the spleen of mice in various groups at the treatment endpoint.

(R–S) IL6 (R), TNFα (S), and INFγ (T) levels in serum obtained from the submandibular vein of mice in different treatment groups at the treatment endpoint.

Data are expressed as mean ± SD (B, D, G, H, J–T); $n = 4$ technical replicates (B), 4 biological replicates (D), or 5 mice per group (E–T). The data were fitted using a logistic model (B). Statistical significance was calculated using two-way ANOVA followed by Šídák's multiple comparison test (D), one-way ANOVA followed by Tukey's multiple comparison test (G, K–T), and one-way repeated measures ANOVA followed by Dunnett's multiple comparison test (H, J). Schematic created using BioRender.com (E).

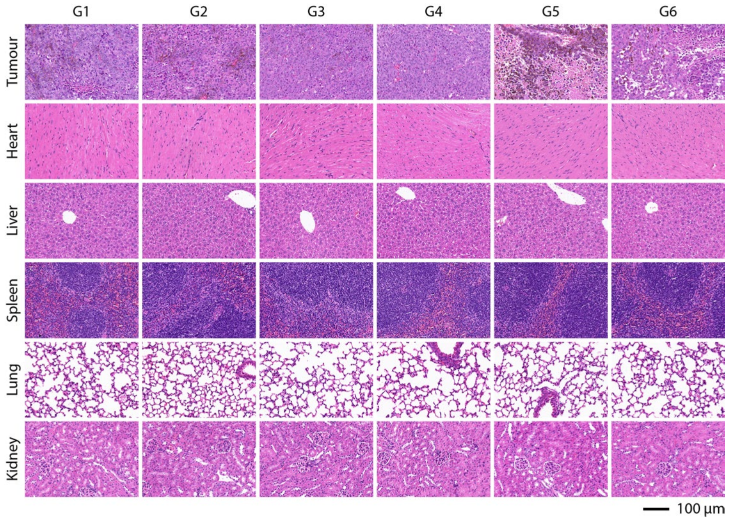

Figure S45. H&E staining in the tumour and the major organs excised from C57BL/6N mice with B16F10 tumours in different treatment groups at Day 12.

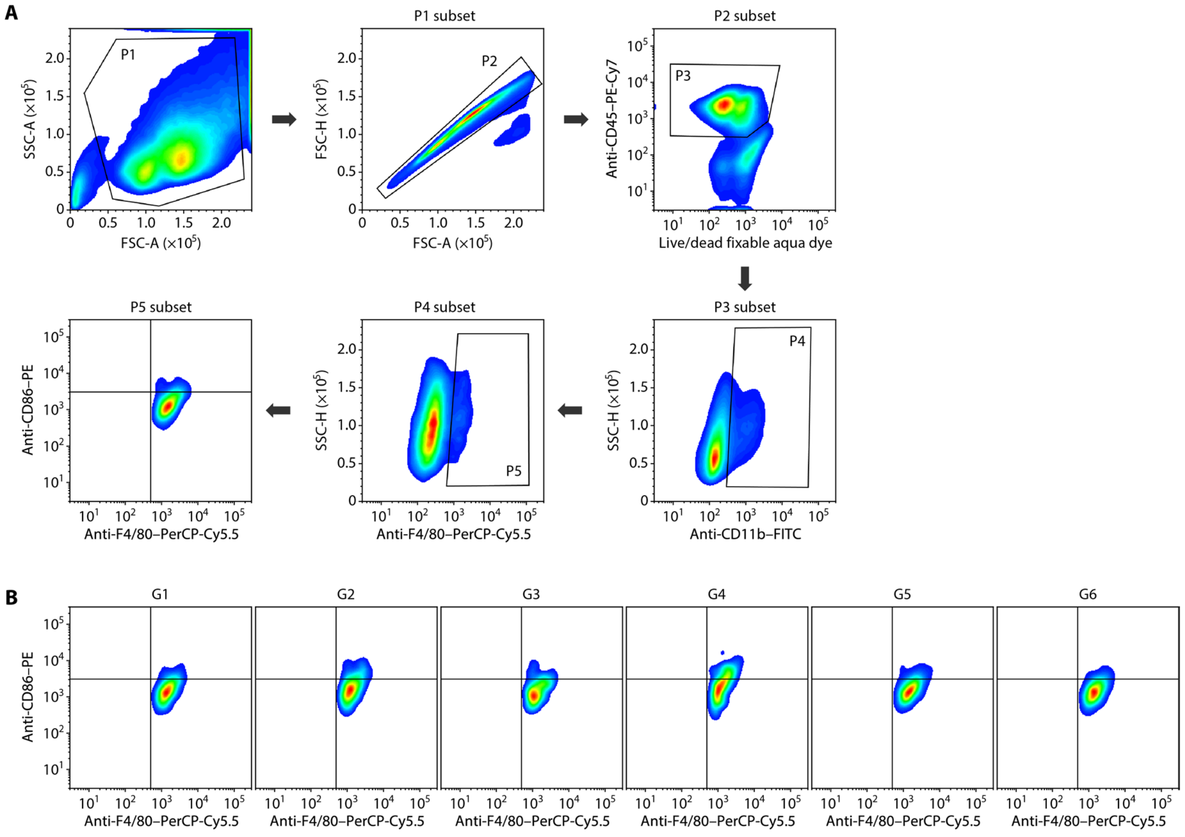

Figure S46. Flow cytometric analysis for classically activated macrophages in spleen of B16F10 tumour-bearing C57BL/6N mice.

(A) Gating strategy.

(B) Representative flow cytometric plots.

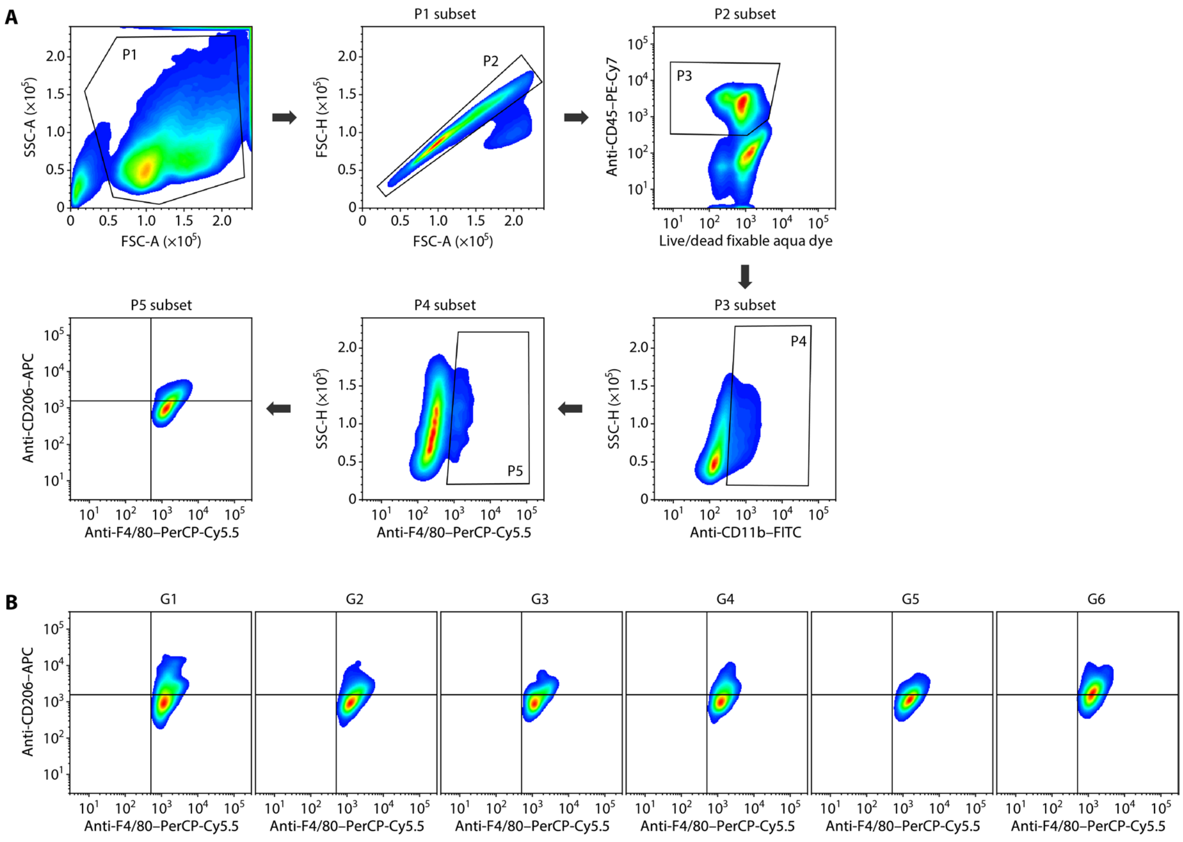

Figure S47. Flow cytometric analysis for alternatively activated macrophages in spleen of B16F10 tumour-bearing C57BL/6N mice.

(A) Gating strategy.

(B) Representative flow cytometric plots.

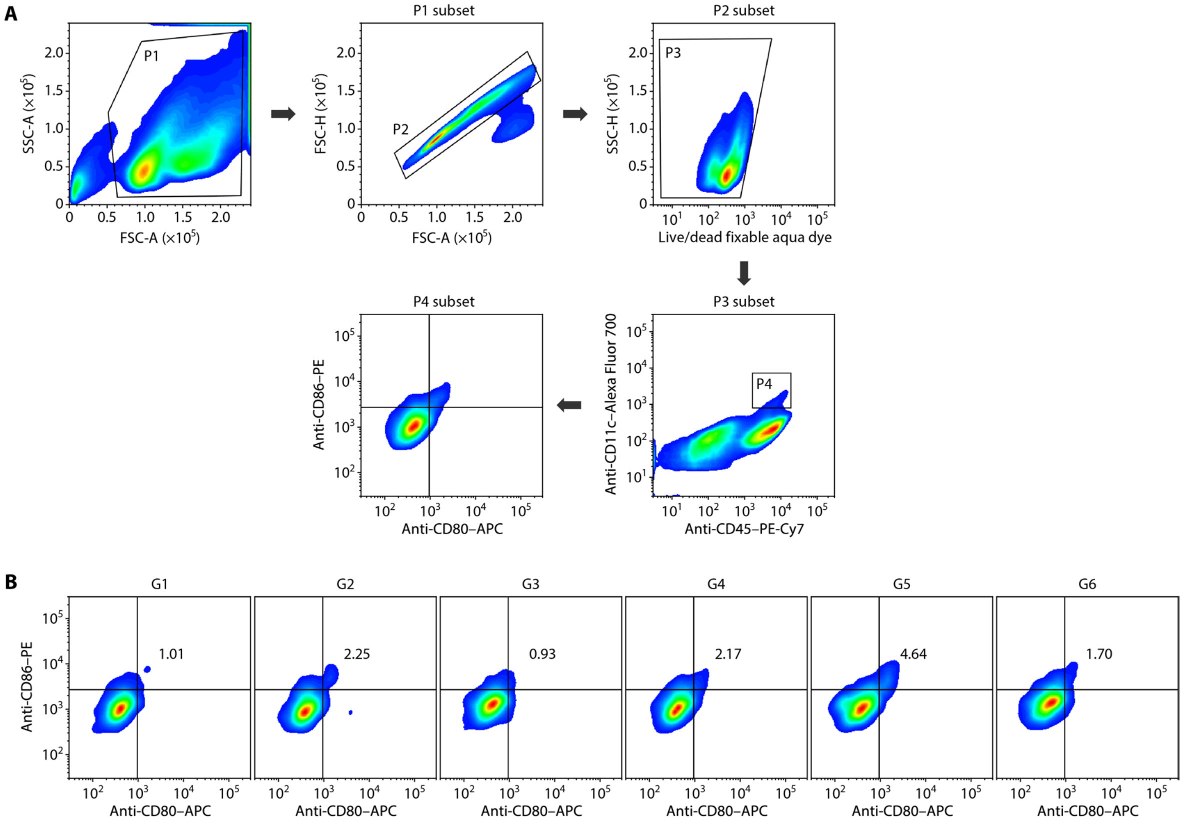

Figure S48. Flow cytometric analysis for maturated DCs in spleen of B16F10 tumour-bearing C57BL/6N mice.

(A) Gating strategy.

(B) Representative flow cytometric plots.

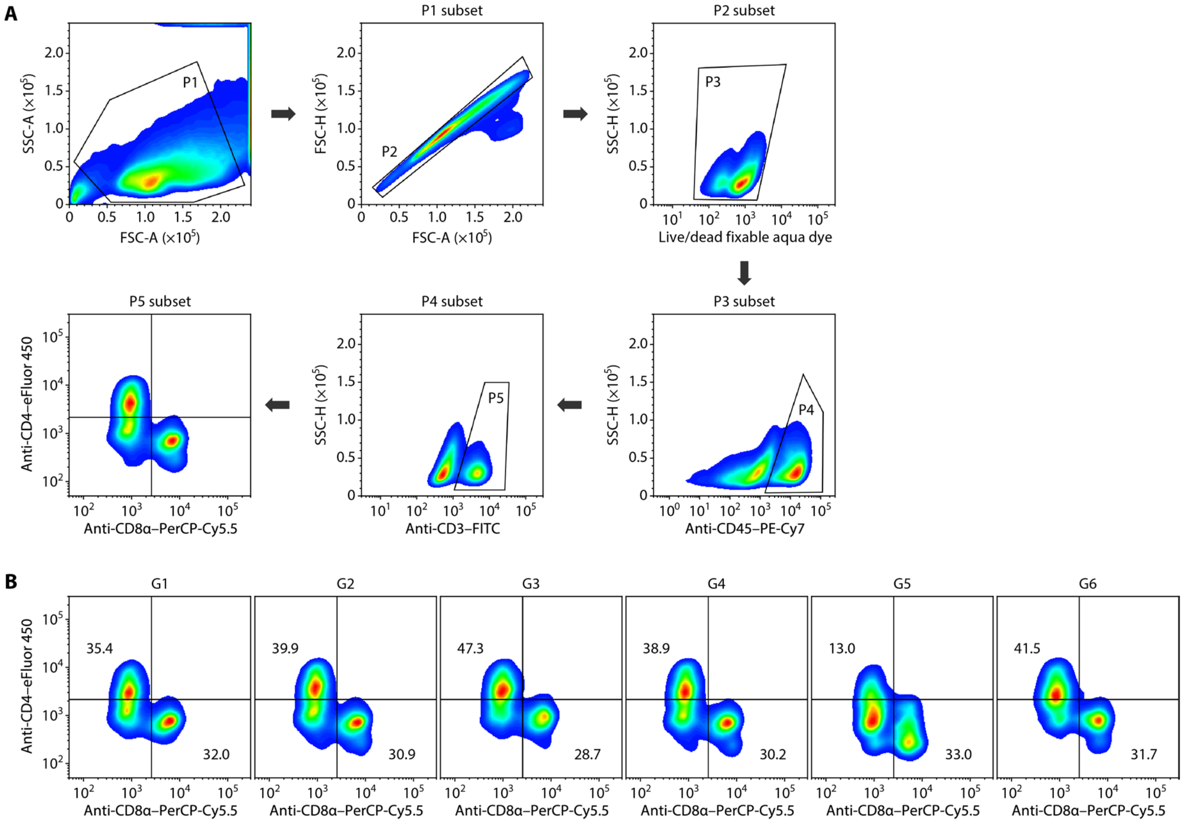

Figure S49. Flow cytometric analysis for T cells in spleen of B16F10 tumour-bearing C57BL/6N mice.

(A) Gating strategy.

(B) Representative flow cytometric plots for evaluating cytotoxic and helper T lymphocytes.

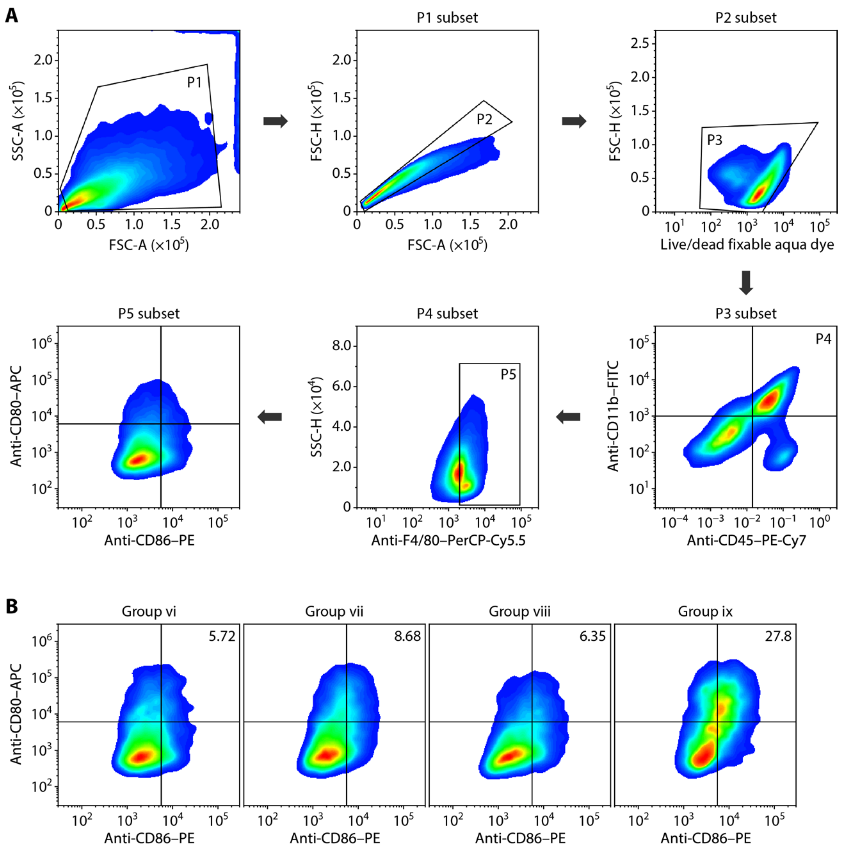

Figure S50. Flow cytometric analysis of classically activated macrophages in the abscopal tumours of bilateral 4T1 tumour-bearing BALB/c mice (groups vi–ix).

(A) Gating strategy.

(B) Representative flow cytometric plots.

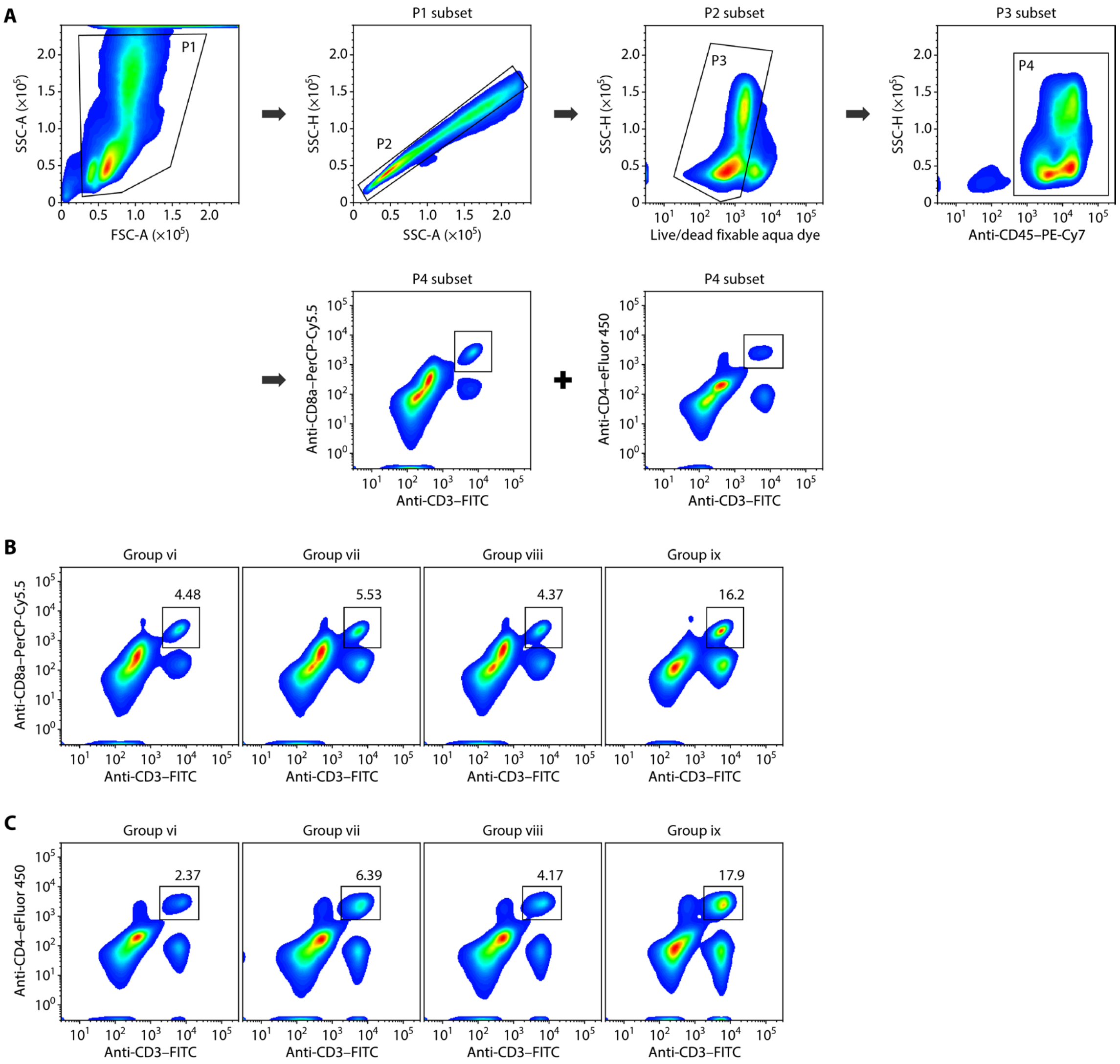

Figure S51. Flow cytometric analysis of T cells in spleen of bilateral 4T1 tumour-bearing BALB/c mice (groups vi–ix).

(A) Gating strategy.

(B, C) Representative flow cytometric plots for evaluating cytotoxic (B) and helper (C) T lymphocytes.

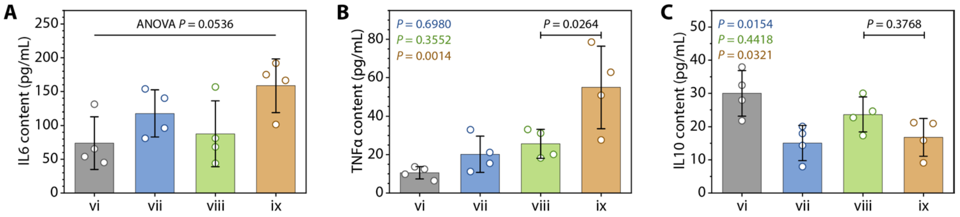

Figure S52. Plasma IL6, TNFα, and IL10 levels.

(A–C) IL6 (A), TNFα (B), and IL10 (C) levels in plasma obtained from the submandibular vein of mice with bilateral 4T1 tumours in different treatment groups at Day 21. Data are expressed as mean ± SD, *n* = 4 mice per group. Statistical significance was determined using one-way ANOVA followed by Tukey's multiple comparison tests.

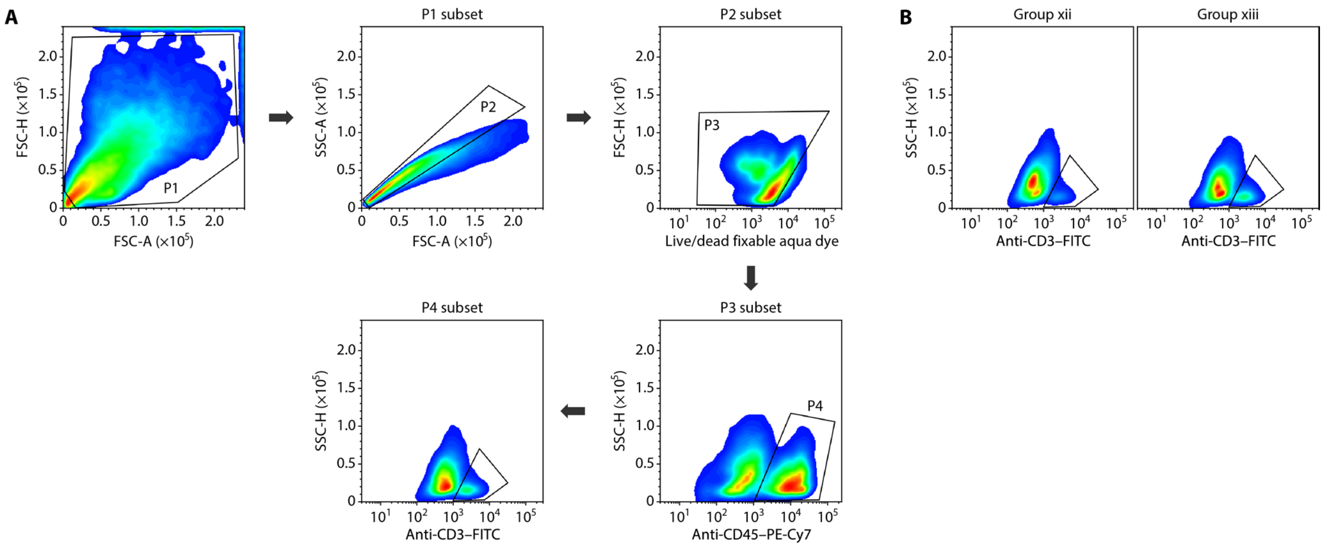

Figure S53. Flow cytometric analysis of T lymphocytes in 4T1 tumour of groups xii and xiii.

(A) Gating strategy.

(B) Representative flow cytometric plots.

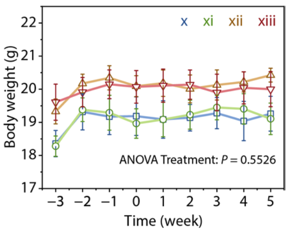

Figure S54. Body weight changes of BALB/c mice in groups x, xi, xii, and xiii.

Data are expressed as mean ± SD, n = 10 mice per group. Statistical significance was determined using three-way repeated measures ANOVA with the Geisser–Greenhouse correction.

Table S1. Crystal data and refinement parameters of (1*E*,1'*E*)-1,1'-(2,5-dimethoxy-1,4-phenylene)bis(*N*-phenylmethanimine).

Empirical formula	$C_{22}H_{20}N_2O_2$	Z	2
Formula weight	344.40	ρ_{calc}/(mg·mm^{-3})	1.278
Crystal system	Monoclinic	μ/mm^{-1}	0.658
Space group	$P2_1/c$	F(000)	364.0
a/Å	16.7985(15)	Collected reflections	3325
b/Å	4.3092(3)	Independent reflections	1666
c/Å	13.3094(12)	R_{int}	0.0294
α/°	90.00	Goodness-of-fit on F^2	1.037
β/°	111.737(11)	Final R indexes ($I \geq 2\sigma(I)$)	R_1 = 0.0462, wR_2 = 0.1321
γ/°	90.00	Final R indexes (all data)	R_1 = 0.0585, wR_2 = 0.1482
Volume/Å^3	894.94(13)	CCDC	2361348

Table S2. Atomic coordinates of UMCOF1.

Atom	*x*	*y*	*z*	Atom	*x*	*y*	*z*
C1	0.24393	−0.44575	−0.91017	C26	0.02330	−0.95611	−0.10750
C2	0.22039	−0.48867	−0.86593	C27	0.04319	−0.97974	−0.10454
C3	0.23801	−0.51078	−0.71108	C28	0.35360	−0.35626	−0.80975
C4	0.27949	−0.48926	−0.59129	C29	0.25986	−0.67129	−0.80048
C5	0.30269	−0.44624	−0.62322	C30	0.07348	−0.66205	−0.11381
C6	0.28541	−0.42356	−0.78358	H31	0.22939	−0.43047	−1.04934
N7	0.21205	−0.55446	−0.65333	H32	0.18839	−0.50493	−0.95880
C8	0.22496	−0.58077	−0.61250	H33	0.29354	−0.50513	−0.45359
C9	0.19525	−0.62483	−0.53405	H34	0.33351	−0.43105	−0.50352
C10	0.15464	−0.63692	−0.41882	H35	0.25751	−0.57086	−0.64355
C11	0.12569	−0.67871	−0.34017	H36	0.14590	−0.61342	−0.39053
C12	0.13808	−0.70850	−0.38024	H37	0.18744	−0.71991	−0.52376
C13	0.17870	−0.69641	−0.49546	H38	0.07583	−0.76248	−0.27074
C14	0.20764	−0.65463	−0.57411	H39	0.03979	−0.82820	−0.46070
C15	0.10837	−0.75256	−0.30179	H40	−0.00018	−0.90229	−0.41076
O16	0.24852	−0.64115	−0.69872	H41	0.10394	−0.90287	0.13506
O17	0.08481	−0.69218	−0.21556	H42	0.14494	−0.82840	0.04452
N18	0.12128	−0.77887	−0.26096	H43	0.07651	−0.96413	−0.10604
C19	0.09533	−0.82256	−0.20321	H44	0.36920	−0.37398	−0.80824
C20	0.05384	−0.84407	−0.32299	H45	0.23640	−0.69517	−0.98930
C21	0.03064	−0.88709	−0.29106	H46	0.26359	−0.68675	−0.55005
C22	0.04792	−0.90977	−0.13070	H47	0.29019	−0.65533	−0.94806
C23	0.08940	−0.88758	−0.00412	H48	0.04314	−0.67800	0.03378
C24	0.11294	−0.84467	−0.04836	H49	0.06974	−0.64658	−0.36423
C25	0.31003	−0.37722	−0.80679	H50	0.09693	−0.63816	0.07502

Table S3. Atomic coordinates of UMCOF2.

Atom	x	y	z	Atom	x	y	z
C1	0.23971	−0.44079	−0.59574	C26	0.02189	−0.95612	−0.10846
C2	0.21476	−0.48367	−0.56469	C27	0.04320	−0.97835	−0.10597
C3	0.22941	−0.50985	−0.67766	C28	0.35051	−0.35907	−0.77750
C4	0.26867	−0.49270	−0.84525	C29	0.26487	−0.65739	−0.39098
C5	0.29318	−0.44988	−0.88813	C30	0.05319	−0.67526	−0.49947
C6	0.27963	−0.42299	−0.75548	H31	0.22799	−0.42197	−0.47803
N7	0.20386	−0.55343	−0.61116	H32	0.18457	−0.49651	−0.43472
C8	0.21706	−0.57961	−0.58244	H33	0.28018	−0.51216	−0.95276
C9	0.18777	−0.62391	−0.50498	H34	0.32240	−0.43826	−1.03214
C10	0.14513	−0.63960	−0.55809	H35	0.24979	−0.56890	−0.60184
C11	0.11616	−0.68160	−0.49967	H36	0.13445	−0.61916	−0.65923
C12	0.13027	−0.70875	−0.38546	H37	0.18370	−0.71338	−0.23134
C13	0.17293	−0.69300	−0.33181	H38	0.06818	−0.76434	−0.30337
C14	0.20189	−0.65103	−0.39097	H39	0.26494	−0.60525	−0.18844
C15	0.10103	−0.75321	−0.31214	H40	0.05321	−0.72723	−0.70549
C16	0.24637	−0.63560	−0.31211	H41	0.03390	−0.83031	−0.46261
C17	0.07172	−0.69700	−0.57948	H42	−0.00383	−0.90446	−0.41458
N18	0.11476	−0.77857	−0.25207	H43	0.10125	−0.90087	0.14312
C19	0.08984	−0.82243	−0.19731	H44	0.14007	−0.82624	0.05877
C20	0.04868	−0.84520	−0.32260	H45	0.07654	−0.96166	−0.10785
C21	0.02676	−0.88827	−0.29227	H46	0.36379	−0.37891	−0.77539
C22	0.04503	−0.90977	−0.12939	H47	0.24837	−0.68743	−0.52459
C23	0.08610	−0.88640	0.00249	H48	0.29740	−0.64469	−0.32807
C24	0.10834	−0.84342	−0.03857	H49	0.02069	−0.68789	−0.56341
C25	0.30718	−0.37693	−0.77535	H50	0.06966	−0.64531	−0.36410

Table S4. Atomic coordinates of UMCOF3.

Atom	x	y	z	Atom	x	y	z
C1	0.08783	−2.94609	−0.20931	C11	0.38540	−2.36968	−0.74760
C2	0.03428	−2.91100	−0.21635	C12	0.26152	−2.51231	−0.66798
C3	0.07041	−2.81789	−0.27156	H13	0.15572	−2.90477	−0.21377
C4	0.15543	−2.76028	−0.17372	H14	0.19378	−2.77985	−0.01868
C5	0.19304	−2.67523	−0.27393	H15	0.25915	−2.63333	−0.20315
C6	0.14510	−2.64466	−0.45424	H16	0.02180	−2.67602	−0.66454
C7	0.05907	−2.69939	−0.52534	H17	−0.04215	−2.82656	−0.53920
C8	0.02237	−2.78569	−0.44512	H18	0.42545	−2.39826	−0.73071
N9	0.18253	−2.55828	−0.57024	H19	0.30337	−2.53886	−0.67521
C10	0.29717	−2.42176	−0.74058				

Table S5. Atomic coordinates of UMCOF4.

Atom	*x*	*y*	*z*	Atom	*x*	*y*	*z*
C1	0.38651	0.01761	0.13916	O29	0.20103	0.22859	–0.26551
C2	0.42145	0.03001	0.09521	O30	0.32041	0.28724	0.09180
C3	0.43132	0.06729	0.06391	C31	0.22459	0.26136	0.34096
C4	0.40132	0.09572	0.04519	C32	0.24469	0.23037	0.32180
C5	0.40449	0.13096	0.11784	N33	0.00127	0.44435	0.05998
C6	0.37786	0.15826	0.08866	C34	0.47944	0.11123	–0.03563
C7	0.34705	0.15043	–0.01140	C35	0.46855	0.07776	0.03263
C8	0.34277	0.11498	–0.07708	N36	0.49906	0.05749	0.06996
C9	0.36990	0.08815	–0.05139	H37	0.36211	0.03387	0.16594
N10	0.31891	0.17737	–0.04356	H38	0.42786	0.13758	0.19522
C11	0.32423	0.21292	–0.04764	H39	0.38102	0.18505	0.14706
C12	0.29229	0.23788	–0.07026	H40	0.31884	0.10847	–0.15232
C13	0.29172	0.27252	–0.00467	H41	0.36623	0.06134	–0.10666
C14	0.25840	0.29565	–0.02482	H42	0.35190	0.22415	–0.03408
C15	0.22832	0.28248	–0.10336	H43	0.25837	0.32299	0.03071
C16	0.19419	0.30498	–0.10790	H44	0.17439	0.30126	–0.20584
N17	0.18872	0.32985	0.00476	H45	0.18723	0.39171	0.17468
C18	0.15735	0.35392	0.01822	H46	0.13647	0.43602	0.19490
C19	0.16123	0.38591	0.11294	H47	0.06882	0.36639	–0.11186
C20	0.13208	0.41100	0.12609	H48	0.11889	0.32153	–0.12994
C21	0.09831	0.40483	0.04446	H49	0.13361	0.46610	–0.08905
C22	0.09427	0.37209	–0.04760	H50	0.03742	0.36284	0.14795
C23	0.12327	0.34656	–0.05927	H51	0.19714	0.26226	0.28647
C24	0.11094	0.48277	–0.04397	H52	0.46245	0.13447	–0.07025
C25	0.07768	0.47100	0.02332	H53	0.49948	1.03345	1.14085
C26	0.02022	0.38695	0.12922	H54	0.33970	0.73094	0.63205
C27	0.03117	0.42230	0.08247	H55	0.24383	0.82240	0.72908
C28	0.06814	0.43338	0.05126				

Table S6. Atomic coordinates of UMCOF5.

Atom	*x*	*y*	*z*	Atom	*x*	*y*	*z*
N1	0.68417	0.73304	0.63342	C11	0.73823	0.69789	0.35148
C2	0.55961	0.74749	0.01695	H12	0.65408	0.63610	0.81966
C3	0.64821	0.74108	0.77115	H13	0.57606	0.64020	1.02914
C4	0.63363	0.68376	0.85400	H14	0.62773	0.84741	0.74866
C5	0.58953	0.68657	0.97510	H15	0.70559	0.83206	0.58501
C6	0.61987	0.80234	0.81318	H16	0.74061	0.86803	0.33300
C7	0.70795	0.78043	0.55023	H17	0.55125	0.85201	0.95768
C8	0.74520	0.81646	0.29782	H18	0.72873	0.65687	0.42842
C9	0.57577	0.80518	0.93379	C19	0.50000	0.75000	0.12500
C10	0.73270	0.76452	0.39942				

Table S7. Crystal data of UMCOF1–5.

	UMCOF1	UMCOF2	UMCOF3	UMCOF4	UMCOF5
Formula	$C_{78}H_{60}N_6O_6$	$C_{84}H_{60}N_6$	$C_{33}H_{21}N_3$	$C_{60}H_{38}N_8O_4$	$C_{164}H_{112}N_{16}$
Crystal system	Trigonal	Trigonal	Trigonal	Tetragonal	Tetragonal
Space group	*P*3 (143)	*P*3 (143)	*P*3 (143)	*P*4*cc* (103)	$I4_1/a$ (88)
a/Å	37.2421	37.2765	18.1261	36.0313	20.0523
b/Å	37.2421	37.2765	18.1261	36.0313	20.0523
c/Å	3.5295	3.5739	3.5981	8.1285	8.8131
α/°	90	90	90	90	90
β/°	90	90	90	90	90
γ/°	120	120	120	90	90
